# Contribution of Noncovalent Recognition and Reactivity to the Optimization of Covalent Inhibitors: A Case Study on KRas^G12C^

**DOI:** 10.1021/acschembio.4c00217

**Published:** 2024-07-11

**Authors:** Nikolett Péczka, Ivan Ranđelović, Zoltán Orgován, Noémi Csorba, Attila Egyed, László Petri, Péter Ábrányi-Balogh, Márton Gadanecz, András Perczel, József Tóvári, Gitta Schlosser, Tamás Takács, Levente M. Mihalovits, György G. Ferenczy, László Buday, György M. Keserű

**Affiliations:** † Medicinal Chemistry Research Group and National Drug Discovery and Development Laboratory, HUN-REN Research Centre for Natural Sciences, Budapest 1117, Hungary; ‡ Department of Organic Chemistry and Technology, Budapest University of Technology and Economics, Budapest 1111, Hungary; § Department of Experimental Pharmacology and the National Tumor Biology Laboratory, National Institute of Oncology, Budapest 1122, Hungary; ∥ Protein Modeling Research Group, Laboratory of Structural Chemistry and Biology, ELTE Institute of Chemistry, Budapest 1117, Hungary; ⊥ Hevesy György PhD School of Chemistry, Eötvös Loránd University, Pázmány Péter sétány. 1/A, Budapest 1117, Hungary; # MTA-ELTE “Lendület”, Ion Mobility Mass Spectrometry Research Group, Budapest 1117, Hungary; ∇ HUN-REN Research Centre for Natural Sciences, Signal Transduction and Functional Genomics Research Group, Budapest 1117, Hungary; ○ Doctoral School of Biology, Institute of Biology, ELTE Eötvös Loránd University, Budapest 1117, Hungary

## Abstract

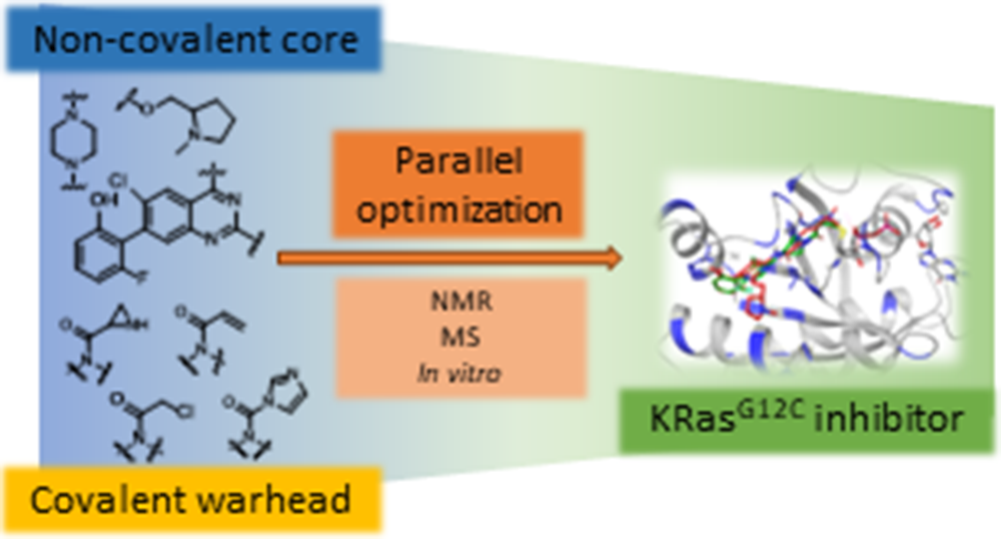

Covalent drugs might bear electrophiles to chemically modify their targets and have the potential to target previously undruggable proteins with high potency. Covalent binding of drug-size molecules includes a noncovalent recognition provided by secondary interactions and a chemical reaction leading to covalent complex formation. Optimization of their covalent mechanism of action should involve both types of interactions. Noncovalent and covalent binding steps can be characterized by an equilibrium dissociation constant (*K*_I_) and a reaction rate constant (*k*_inact_), respectively, and they are affected by both the warhead and the scaffold of the ligand. The relative contribution of these two steps was investigated on a prototypic drug target KRAS^G12C^, an oncogenic mutant of KRAS. We used a synthetically more accessible nonchiral core derived from ARS-1620 that was equipped with four different warheads and a previously described KRAS-specific basic side chain. Combining these structural changes, we have synthesized novel covalent KRAS^G12C^ inhibitors and tested their binding and biological effect on KRAS^G12C^ by various biophysical and biochemical assays. These data allowed us to dissect the effect of scaffold and warhead on the noncovalent and covalent binding event. Our results revealed that the atropisomeric core of ARS-1620 is not indispensable for KRAS^G12C^ inhibition, the basic side chain has little effect on either binding step, and warheads affect the covalent reactivity but not the noncovalent binding. This type of analysis helps identify structural determinants of efficient covalent inhibition and may find use in the design of covalent agents.

## Introduction

Modulation of protein functions by covalent modifications has become a popular approach in drug discovery. The selective labeling of catalytic residues prevents enzymes from performing their catalytic role and inhibits undesired reactions. Labeling of residues not directly involved in the catalytic machinery but affecting substrate binding may also lead to inhibition. The chemical modification of proteins has potential advantages over inhibition by noncovalent association. These advantages include high affinity, distinct pharmacodynamic profile and the ability to target proteins not accessible by noncovalent agents.^1^ Residues most often targeted are cysteines owing to the high nucleophilicity of their thiolate form. Moreover, cysteines occur with low frequency in the proteome and this reduces concerns about unspecific labeling. Other residues may also act as nucleophiles and an increasing number of covalent inhibitors, including FDA-approved drugs, targeting other than cysteine residues have been reported.^1−3^

The binding of covalent agents to a nucleophilic residue is often assumed to be a two-step process starting with the noncovalent association of the ligand and the protein. In this noncovalent complex, the warhead of the ligand is placed in proximity to the nucleophilic residue in a position that allows the subsequent chemical reaction. The chemical reaction may be both reversible and irreversible and the latter is more abundant among reported inhibition mechanisms. According to the two-step model of irreversible binding, both the low dissociation constant of the noncovalent complex and the high rate constant of the chemical reaction contribute to the efficiency of the covalent inhibition (eq 1).
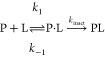
1

It is often assumed that the noncovalent complex formation is a fast equilibrium process with an equilibrium constant of *K*_I_ = *k*_–1_/*k*_1_. Then, *K*_I_ and *k*_inact_ characterize ligand affinity as they determine the pseudo-first-order rate constant of inactivation (*k*_obs_).^4^ Both *K*_I_ and *k*_inact_ can be experimentally measured and computed typically by molecular dynamics simulations. However, the separate accurate experimental determination of *K*_I_ and *k*_inact_ is not always feasible and then the *k*_inact_/*K*_I_ ratio readily obtained for small inhibitor concentrations can be used to characterize the affinity.^5^ It is also to be noted that the IC_50_ value widely used in characterizing the activity of noncovalent inhibitors becomes time-dependent when covalent inhibitors continuously decrease the amount of active enzyme in the system. In contrast, *K*_I_ and *k*_inact_ provide a time-independent measure of the efficiency of covalent inhibition.

The two-step model with fast equilibrium noncovalent complex formation allows the separation of the recognition process characterized by the *K*_I_ equilibrium constant from the chemical reaction characterized by the *k*_inact_ rate constant. Considering now various ligands binding to the same protein with varying affinity, one may assume that the ligand warhead is primarily responsible for the reactivity variations, while the scaffold of the ligand, and, in particular, its specific pharmacophore elements are primarily responsible for the variations in the noncovalent association. However, warheads also contribute to the recognition and scaffold variations, resulting in even modest changes in the geometry of the prereaction complex that may modify reaction barriers. In the present study, we examine how the systematic variation of the scaffold and warhead—via changing *K*_I_ and *k*_inact_—affects the efficiency of covalent inhibitors against a prototypic drug target KRAS^G12C^.

KRAS is a member of the small membrane-bound Ras proteins and acts as a molecular switch in a number of downstream signaling pathways.^6^ KRAS is activated by GTP binding and deactivated by GTP hydrolysis to form GDP. These processes are supported by guanine nucleotide exchange factors (GEFs) and GTPase activating proteins (GAPs). The N-terminal G domain involves three main functional regions: the phosphate-binding loop (P-loop), switch I and switch II regions. The two switch regions have different conformation in the active and inactive state, the GTP bound active state being more rigid, then the relaxed inactive GDP bound state.^7^ Oncogenic KRAS mutations occur mostly on Gly12 in the phosphate binding loop that interferes with GAP binding, thus blocking the GAP-catalyzed hydrolysis of GTP. The resulting hyperactivated proteins are responsible for the constant activation of downstream signaling pathways and hence the development of ∼30% of human cancer. The three most common mutations of G12 are to aspartic acid (G12D, 41%), valine (G12 V, 28%), and cysteine (G12C, 14%).^8^

The frequency of the KRAS mutations in human cancer makes the protein a desirable target.^9,10^ However, the natural substrate GTP is highly abundant in the human body. That, together with its picomolar affinity to Ras proteins, makes orthosteric inhibition challenging. Furthermore, KRAS has an essential role in homeostasis,^11^ so specific targeting of the oncogenic mutants might be advantageous. A feasible approach is targeting the allosteric binding site near the nucleotide-binding pocket and adjacent to the mutated Gly12 (the so-called switch II binding pocket). In KRAS^G12C^—one of the three most frequent mutations causing cancer—selective inhibition can be achieved by forming a covalent bond between the inhibitor and the nucleophilic Cys12.

**Figure 1:**
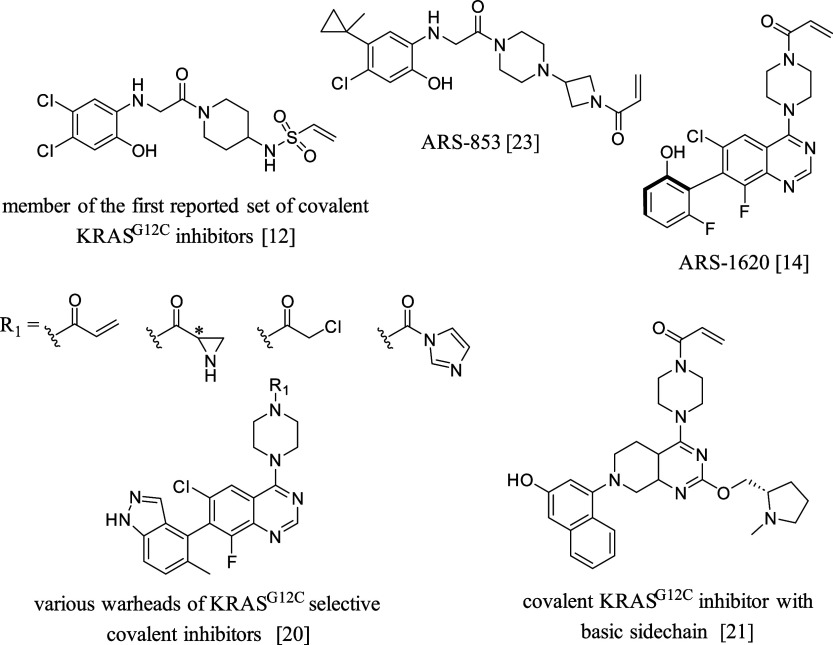
Selected KRAS^G12C^ inhibitors.

The first set of covalent inhibitors against KRAS^G12C^ was reported by Shokat and co-workers in 2013^12^ (Figure 1). These molecules opened the possibility of mutant selective inhibition, taking advantage of the nucleophilic nature of the non-native cysteine. Further optimization resulted in a set of in vivo active compounds with quinazoline core, e.g., ARS-1620^13,14^ (Figure 1). However, ARS-1620 contains axial chirality, and the separation of the atropisomers is challenging and requires special techniques of chromatography.^12^ Therefore, since then several further attempts have been made to develop irreversible and reversible covalent inhibitors targeting the catalytic or noncatalytic amino acids of KRAS.^15−17^

The majority of reported KRAS^G12C^ inhibitors, including those in clinical trials contain acrylamide warhead.^18,19^ In order to screen further potential warheads targeting Cys12, several electrophiles were tested against KRAS^G12C^ by McGregor and co-workers. Five warheads were found to have high affinity toward the mutant KRAS^G12C^ with no or slight binding to the wild-type protein, in particular, the acrylamide, two aziridine isomers, chloroacetamide, and acyl imidazole.^20^

In addition to these attempts, several efforts to optimize the noncovalent contacts were published. A structural study by Fell et al. identified a histidine (His95) and glutamine (Glu62) located at the switch II pocket that was suggested to interact with a basic side chain attached to the quinazoline core. In order to utilize these interactions, several basic groups were introduced to improve binding affinity. These substituents increased covalent protein modification in varying degrees and the best results were obtained by *N*-methyl-l-prolinol, which produced a 70 nM IC_50_ of the suppression of ERK phosphorylation in H358 cells^21^ (Figure 1).

**Figure 2:**
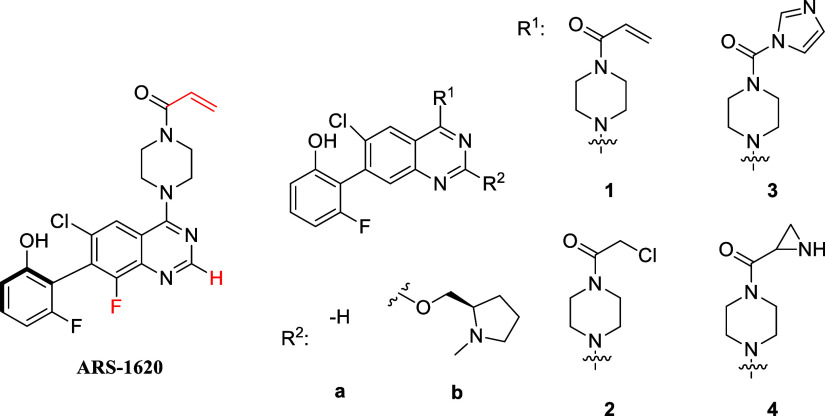
Designed ARS-analogues and ARS-1620 with modified atoms in red.

Based on these precedents we aimed to prepare molecules with a quinazoline scaffold but eliminate the axial chirality of ARS-1620 by the removal of the 8-F atom. Investigating the effect of the basic side chain combined with different warheads, we designed and synthesized a set of compounds equipped with four different warheads (Figure 2). Target engagement of the synthesized compounds was tested by HSQC NMR and LC-MS/MS. Their functional activity was assessed in a nucleotide exchange assay, and finally, their IC_50_ values were measured against four different cancer cell lines bearing KRAS^G12C^ and KRAS^WT^ in order to test their selectivity for the target. Our aim was to explore how the systematic structural variation of the warhead and the scaffold affects the noncovalent and covalent binding steps and the overall affinity of the compounds.

## Results

### Synthesis of ARS Analogues

**Scheme 1:**
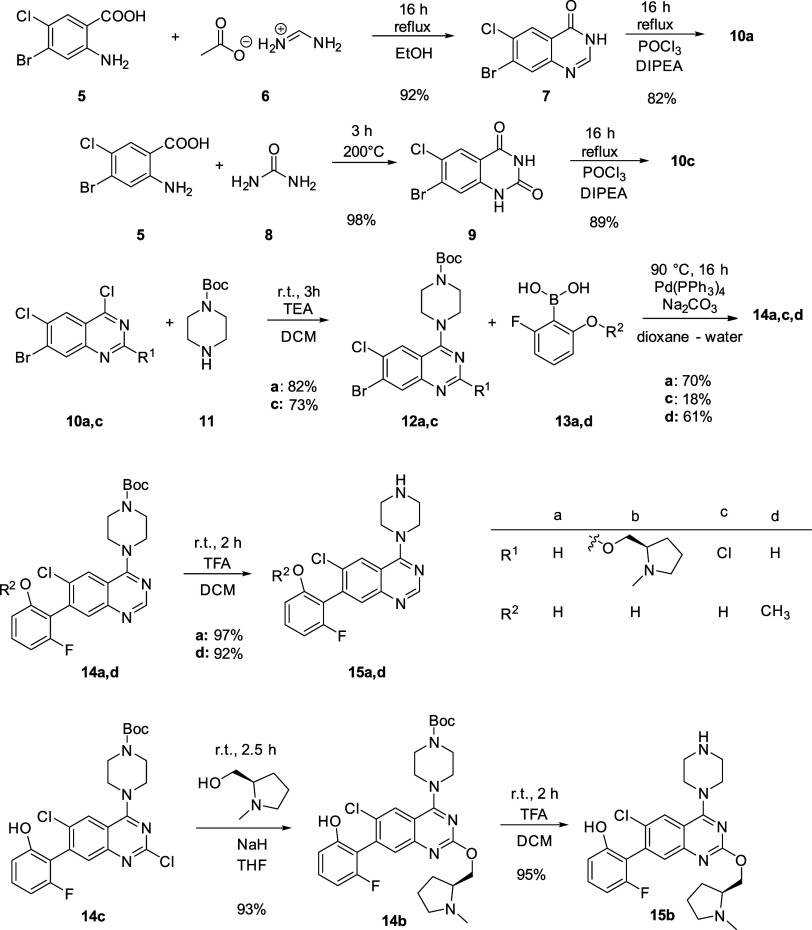
Synthesis of the Noncovalent Cores (**15a,b,d**) Used for Inhibitors **1**–**4a,b**

The synthesis of compounds **1a–4a** was started by the formation of the quinazoline ring via condensation, then refluxing in POCl_3_ resulted in molecules **10a**,**c**.^14^ After coupling with the Boc-protected piperazine through an S_N_Ar reaction, a Suzuki coupling was carried out. Then, the *N*-methyl-L-prolinol side chain was attached to **14c** using NaH, followed by the deprotection of the piperazine (Scheme 1).

**Scheme 2:**
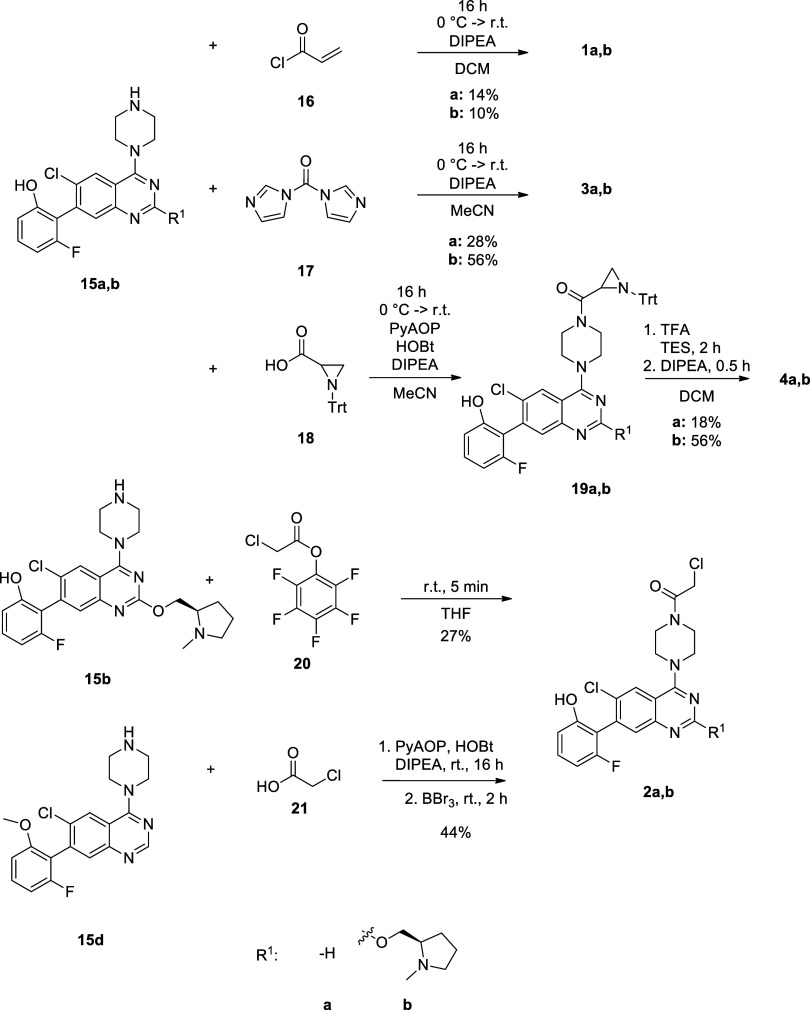
Warhead Coupling for Inhibitors **1**–**4a,b**

Next, the collection of warheads was introduced to the noncovalent cores that required specific synthetic routes in some cases (Scheme 2). Acrylamide and acyl imidazole were attached using acylating agents **16** and **17** simply in the presence of DIPEA as a base. For the reaction with carboxylic acid **18** additional coupling agents, PyAOP and HOBt were used, then the aziridine was deprotected by TFA at RT. In the case of **2a**, acylation was carried out using chloroacetic acid (**21**) with PyAOP and HOBt. In order to prevent the reaction with the phenolic OH, a methyl-protecting group was applied (**15d**), which was removed after the coupling of the warhead by BBr_3_. This approach, however, was not feasible for **2b**, as the demethylation also affected the methyl group on the prolinol side chain. Several other protecting groups (e.g., ^*t*^Bu-, THP-, trimethylsilyl-, ^*t*^butyl-dimethylsilyl-, benzyl-chloromethyl-ether) were tested with little success to avoid the dealkylation, so we chose an NH-selective acylation using pentafluorophenolate **20**. The reaction proceeded quickly and smoothly with total conversion forming only 10% side product.^14^ The final products (**1–4a** and **1–4b**) were purified via preparative HPLC, and their structure was confirmed by ^1^H and ^13^C NMR spectroscopy and mass spectrometry (see the Experimental Section).

### Confirmation of Covalent Binding and Identification of the Binding Site

Our first aim was to determine whether the compounds bind to the target protein, KRAS^G12C^. Therefore, **1–4a**,**b** were incubated with the protein in a hundred-fold excess, then HSQC NMR spectra were recorded (Figure S1). The assigned spectrum of the free protein^22^ was compared with the spectra of the treated KRAS^G12C^, and from the chemical shift perturbation (CSP), the affected amino acids could be determined (see the Supporting Information). The greatest CSPs were observed around the binding pocket (e.g., G10, A11, C12, G13, G60, E62, Y96), hence, in all cases, we presumed that the compounds bound to the switch II pocket around the targeted amino acid. The bound spectra were compared to the CSPs caused by ARS-853^23^ and ARS-1620 (see the Supporting Information) confirming that **1–4a**,**b** bound to the same ligand binding site of KRAS^G12C^ as these ARS molecules.

**Figure 3:**
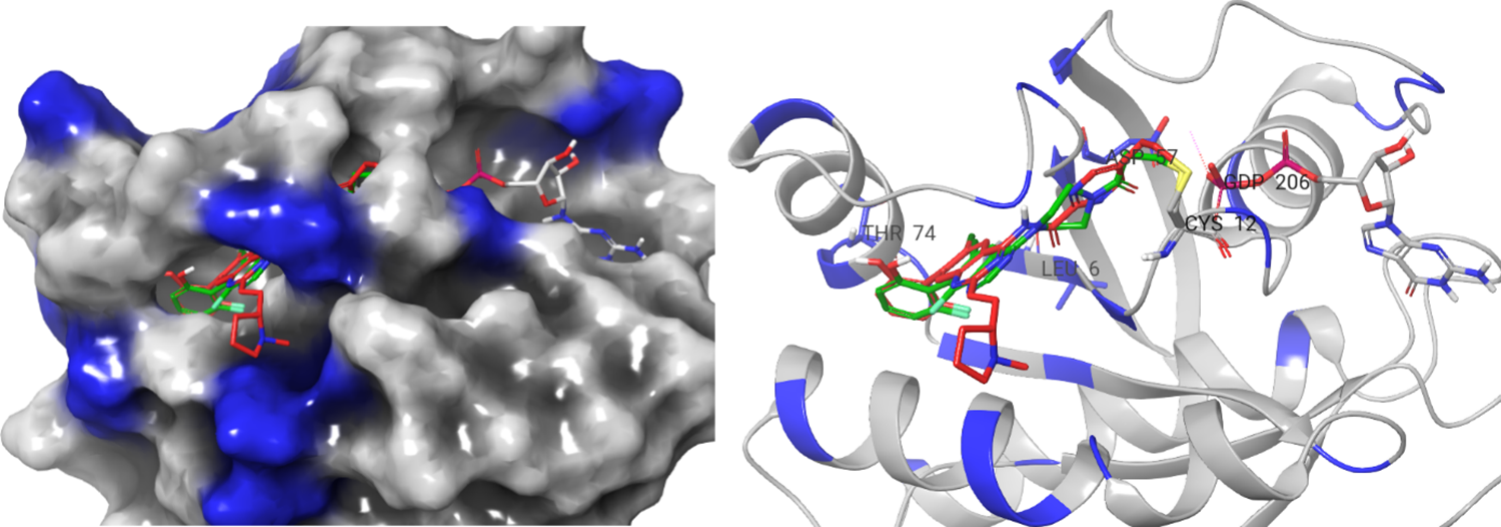
Modeling the binding mode of **1b** (red) into the KRAS^G12C^–ARS-1620 complex X-ray structure (PDB ID: 5 V9U) and ARS-1620 (green), the amino acids showed in blue are the contact residues observed in the HSQC-NMR spectra (the image was produced with Maestro^24^).

To unequivocally determine the labeling position and the covalent nature of the binding, intact MS measurements were performed as well as LC-MS/MS peptide mapping after enzymatic digestion by ProAlanase and trypsin (Table S1). Intact protein molecular mass data and fragmentation profiles of the modified peptides confirmed that the compounds bind to the target Cys12. The site of labeling together with the contact residues identified by HSQC NMR allowed us to model the binding mode of the compounds. Modeling was performed on the protein taken from the KRAS^G12C^–ARS1620 complex X-ray structure (PDB ID: 5V9U) using Schrödinger’s induced fit docking followed by covalent docking simulations. These experiments showed us that the core is positioned similarly to the atropisomeric ARS-1620 as exemplified by **1b** shown in Figure 3, and the basic side chain adopts a conformation and has the interactions shown in the X-ray structure of complexes with similar side chains (PDB: 6UT0 and 6N2K).

### Stability and Reactivity Characterization

The cysteine reactivity of the compounds was first assessed in a surrogate kinetic assay.^25,26^ Glutathione (GSH), a cysteine surrogate, was used in a large excess to ensure quasi-first-order reaction kinetics. Control experiments were performed without GSH to characterize the aqueous stability of the compounds and to correct the observed GSH reactivities with aqueous degradation (see details in the Experimental Section). The GSH reactivities are shown as half-lives in Table 1.

**Table 1:** GSH Reactivity (*t*_1/2_), Inhibition of Nucleotide Exchange (IC_50_), and Covalent Binding Characteristics (*k*_inact_, *K*_I_, and *k*_inact_/*K*_I_) of Compounds **1**–**4a,b** and ARS-1620

compound	*t*_1/2_ GSH (h)	IC_50_ (μM)^a^	*Κ*_Ι_ (μM)^a^	*k*_inact_ (0.001 s^–1^)^a^	*k*_inact_/*K*_I_ (M^–1^ s^–1^)
**1a**	19 ± 1	0.827 ± 0.137	34.8 ± 9.3	10.1 ± 1.0	290.2 ± 82.7
**1b**	44 ± 8	0.520 ± 0.108^b^	107.6 ± 10.7	31.7 ± 4.1	294.6 ± 48.1
**2a**	9 ± 4	0.305 ± 0.040	40.1 ± 11.7	19.2 ± 0.9	478.8 ± 141.5
**2b**	4 ± 0.1	0.642 ± 0.115	147.9 ± 10.3	14.9 ± 1.9	100.7 ± 14.6
**3a**	35 ± 11	67.4 ± 11.3	40.9 ± 16.7	0.4 ± 0.1	9.8 ± 4.7
**3b**	40 ± 18	243.2 ± 52.4	42.2 ± 9.9	0.13 ± 0.01	3.1 ± 0.7
**4a**	>72	198 ± 39.0	22.2 ± 1.8	0.091 ± 0.002	4.1 ± 0.3
**4b**	>72	84.4 ± 18.9	112.2 ± 30.1	1.7 ± 0.30	15.2 ± 4.9
**ARS-1620**	15 ± 2	0.136 ± 0.019	17 ± 3	26 ± 2	1529 ± 294

a Values are shown as mean ± SD and were determined as described in the Experimental Section. *n* = 3 for IC_50_ and *n* = 2 for *t*_1/2_, *k*_inact_ and *K*_I_ determinations.

b 176.6 ± 70.2 μM against KRAS^wt^.

The GSH reactivity of the chloroacetamides (**2a,b**) is the highest followed by comparable reactivities of acrylamides (**1a,b**) and imidazole carboxamide (**3a,b**). Aziridine carboxamides (**4a,b**) show lower reactivity. It is worth noting that the warheads of **3–4a,b** are only partially protonated at pH 7.4 and this may contribute to lower observed reactivities.^27,28^

### Inhibitory Activity in Nucleotide Exchange Assay

After confirming the formation of the covalent bond with Cys12, we tested the influence of the inhibitors on protein function using a nucleotide exchange assay simulating the exchange of GDP to GTP. In theory, the inhibitors bound to Cys12 at the allosteric site can block the activation of the protein. In the assay, the inactive MANT-GDP-bound protein was first incubated with the small molecules at 5, 25, and 125 μM concentrations, then GTP-analogue was added to see whether the inhibitors can prevent the protein from its activation in the presence of SOS1. In the experiments, we used the GTP analogue, GppNHp, which cannot be hydrolyzed by Ras. Compounds **1–2a,b** show submicromolar IC_50_, while **3–4a,b** are considerably less active having IC_50_ around 100 μM (Table 1). The IC_50_ of **1b** against KRAS^wt^ was also determined and found to be ∼200-fold higher than that against KRAS^G12C^.

### Cellular Efficacy and Selectivity

The cellular efficacy and selectivity of the molecules were assessed on KRAS^G12C^ (H1792, H358 lung cell lines, MiaPaca2 pancreas cell line) and KRAS^wt^ (LCLC-103H lung cell line) expressing cells. The cells were treated with reference ARS-1620 and the synthesized inhibitors in 3 replicates per concentration and 3 independent experiments. The cells were then incubated at 37 °C for 72 h and IC_50_ values were determined (Table 3). Compounds **2a** and **2b** showed 3.5–5.7-fold higher antitumor activity than ARS-1620 on H1792, while on other KRAS^G12C^ cell lines, ARS-1620 was the most potent. Compound **1b** revealed IC_50_ in the nanomolar range on MiaPaca2 cells, and moreover, it had better selectivity for KRAS^G12C^ mutation than ARS-1620 for this cell line. Additionally, compound **1b** showed higher selectivity than ARS-1620 for mutation on H358 cells as well. Considering the H1792 cell line, all ARS analogs were more selective for mutation than the control compound. The high selectivity of **1b** observed for all KRAS^G12C^ expressing cell lines versus the KRAS^wt^ expressing cell line (LCLC-103H) is in accordance with the selectivity observed in the nucleotide exchange assay (0.520 ± 0.108 KRAS^G12C^ vs 176.6 ± 70.2 μM KRAS^wt^).

### Labeling Efficiency by LC-MS/MS

*K*_I_ and *k*_inact_ determinations were based on concentration-dependent occupancy measured by MS. In these experiments, four concentrations (20, 40, 80, 160 μM) of the probes **1a–4b** were incubated with KRAS^G12C^ and sampled in certain time intervals until the saturation of the labeling. Single occupancy levels were plotted against time (Figure S2) to obtain *k*_obs_ (Table S2) for each inhibitor concentration (*c*) using a one-phase decay model. The *k*_obs_–*c* function exhibited nonlinearity for each system (Figure S3) and *K*_I_ and *k*_inact_ (Table 1) were obtained by fitting the  function. The *K*_I_ values show limited variation all being in the 10–100 μM range. In contrast, the *k*_inact_ values vary significantly between about 10^–4^ and 3 × 10^–2^ s^–1^.

### Computed Binding Free Energies

The noncovalent recognition of the ligand-protein binding was investigated by calculating the binding free energy differences using thermodynamic integration for compounds **1–4a,b**. (Table 2). Calculated ΔΔ*G*_bind_ values cover a limited range within 2.6 kcal/mol corresponding to less than 2 orders of magnitude K_I_ variation at 310 K. Δ*G*_bind_ for **1a** was set to 6.1 kcal/mol corresponding to the experimental *K*_I_ of 34.8 μM (Table 1) and Δ*G*_bind_ for other compounds was obtained by adding calculated ΔΔ*G*_bind_.

**Table 2:** Experimental and Calculated Binding Free Energies (Δ*G*_bind_) and Experimental Free Energy Barriers (Δ*G*^#^) of Compounds **1–4a,b** and ARS-1620

compound	experimental Δ*G*_bind_ (kcal/mol)^a^	calculated Δ*G*_bind_ (kcal/mol)^b^	experimental Δ*G*^#^(GSH) (kcal/mol)^c^	experimental Δ*G*^#^ (KRAS^G12C^)(kcal/mol)^d^
**1a**	–6.1 ± 0.3	–6.1	19.3 ± 0.0	20.1 ± 0.1
**1b**	–5.4 ± 0.2	–7.1 ± 0.6	19.8 ± 0.1	19.5 ± 0.1
**2a**	–6.0 ± 0.3	–6.6 ± 0.6	18.8 ± 0.2	19.8 ± 0.0
**2b**	–5.2 ± 0.3	–7.6 ± 0.1	18.4 ± 0.0	19.9 ± 0.1
**3a**	–6.0 ± 0.4	–5.0 ± 0.9	19.6 ± 0.2	22.0 ± 0.2
**3b**	–6.0 ± 0.1	–5.1 ± 0.8	19.7 ± 0.3	22.7 ± 0.0
**4a**	–6.3 ± 0.1	–5.6 ± 0.2	>20.1	22.9 ± 0.0
**4b**	–5.4 ± 0.2	–7.1 ± 0.1	>20.1	21.2 ± 0.1
**ARS-1620**	–6.5 ± 0.1	–7.2 ± 0.1	19.1 ± 0.1	19.6 ± 0.1

a Experimental Δ*G*_bind_ obtained as *RT* ln(*K*_I_) with *K*_I_ in M units.

b Relative binding free energies (ΔΔ*G*_bind_) were obtained in three simulations and Δ*G*_bind_(**1a**) was set to −6.1 kcal/mol corresponding to the experimental *K*_I_.

c Calculated as  with *R*, *T*, *k*_b_, *h*, *t*_1/2_, and *c*_GSH_^–^ being the universal gas constant, absolute temperature, Boltzmann constant, Planck constant, half-life, and GSH^–^ concentration, respectively. The GSH^–^ concentration is obtained as .

d Calculated as

## Discussion

The effect of scaffold variation on the recognition can be analyzed by pairwise comparing Δ*G*_bind_ values of compounds with (**b** series) and without (**a** series) the *N*-methyl-l-prolinol substituent (Table 2). The binding free energy differences are close to 1 kcal/mol for all compound pairs suggesting highly similar binding modes and interactions. This is in line with the similar binding modes observed in the experimental complex structures of analogues with (PDB codes 6UT0 and 6N2K) and without linker-connected basic substituents (PDB codes 6OIM and 6B0Y). The similar affinity of the compounds with and without the basic substituent is somewhat surprising as its significant beneficial effect on the suppression of ERK phosphorylation in an H358 KRAS^G12C^-driven cell line was reported.^21^ Analyzing the interactions in the X-ray structure of ARS analogues containing a basic substituent similarly to the **b** series of our compounds (PDB codes 6UT0 and 6N2K), the substituent is directed toward the receptor surface and forms a salt-bridge with Glu62 of a surface loop (Figure S4a). This loop adopts varying conformation in complex structures (cf. 6UT0, 6OIM, and 5 V9U), suggesting flexibility that acts against fixing by ligand-protein interactions (Figure S4b). These observations together with our experimental and computational results support that the *N*-methyl-l-prolinol moiety does not contribute importantly to the binding free energy of ARS analogues. However, it significantly affects the physicochemical properties of the compounds and may contribute to beneficial cellular and in vivo activity.^27^

Considering the effect of warhead on the noncovalent binding, the Δ*G*_bind_ values were compared within the group of **1a**, **2a**, **3a**, and **4a** compounds on the one hand and **1b**, **2b**, **3b**, and **4b** on the other. The warhead dependence is modest both within the **a** and **b** series showing that warhead variation does not have a significant effect on the noncovalent binding probably because their contribution is less important than that of the scaffold. Although KRAS^G12C^ inhibitors were developed along the electrophile-first approach whereby a covalent ligand was searched from the outset,^12^ scaffolds developed^19^ to interact with the protein and present the warhead to Cys12 are primarily responsible to the ligand-protein recognition. Nevertheless, it is worth noting that the noncovalent binding affinity of KRAS^G12C^ inhibitors is the modest, being in the 10 μM or higher range corresponding to approximately −6 kcal/mol or less negative binding free energy. This moderate affinity, primarily provided by the scaffold is sufficient for the ligands to bind in a position that assures a selective chemical reaction with Cys12. This is also illustrated by the affinity of **1b** toward KRAS^wt^. The measured IC_50_ of 176.6 ± 70.2 μM characterizes the noncovalent affinity as no cysteine is available for the chemical reaction between the bound ligand and the protein. The important difference between the IC_50_ of wildtype and G12C mutant (176.6 ± 70.2 vs 0.520 ± 0.108 μM) demonstrates the critical role of the warhead and the covalent binding in providing selectivity.

Calculated binding free energy differences are also small, although somewhat higher than the experiment-derived differences (Table 2; 2.6 vs 1.1 kcal/mol maximal differences). Taking into account the precision of alchemical free energy predictions^29,30^ and the tendency of force field-based simulations to overestimate the stability of the extra salt bridge present in the **b** series (*N*-methyl-l-prolinol-Glu62),^31,32^ the calculated binding free energy differences are also in accordance with similar molecular recognitions irrespective of the warhead and scaffold variations.

The rate of chemical bond formation, *k*_inact_, in the second step of covalent inhibitor binding (eq 1) is associated with the rate-limiting step of the chemical reaction. However, the assignment of *k*_inact_ to a single rate-limiting step for the KRAS^G12C^ inhibitors is problematic for multiple reasons. Here, we investigate compounds with various warheads and distinct reaction mechanisms. In addition, although cysteine deprotonation was identified as the first step in cysteine labeling reactions in other proteins,^33−35^ there is no obvious proton acceptor near the Cys12 thiol and there are conflicting estimates for the p*K*_a_ of Cys12 based on pH-dependent reactivity and NMR measurement.^36,37^ Nevertheless, the experimental pseudo-first-order rate constant of inactivation (*k*_obs_)^4^ can be well fitted with the parameters of the two-step model to obtain *k*_inact_ and *K*_I_. Since *k*_inact_ exhibits warhead dependence (see below), the reactivity of the warhead clearly affects *k*_inact_, however, other steps, like cysteine deprotonation or C_α_ protonation for the **1a,b** Michael acceptors (as proposed for the acrylamide warhead of ARS-853^38^) may also influence *k*_inact_. It is worth noting that although reaction rate constants of covalent ligand binding can be calculated by QM/MM umbrella sampling simulations,^39,40^ this was not attempted for the investigated systems owing to the lack of unambiguous assignment of a rate constant to *k*_inact_.

Scaffold variation may affect reactivity either by modulating the reactivity of the warhead or by affecting the binding mode and thus the presentation of the warhead to the reactive residue. A comparison of the compounds with and without the *N*-methyl-l-prolinol substituent, the **a–b** pairs, quantifies the effect of the skeleton variation on the reactivity. The increased rigidity of the binding pose might hinder the warhead from reaching the Cys residue and might affect the prereaction, the transition state, and the postreaction geometries. If such an effect was present, then it was potentially warhead geometry and reaction-mechanism-dependent. However, the binding mode does not importantly change with the introduction of the *N*-methyl-l-prolinol group (see above), and the flexible *O*-ethyl chain acts again constraining the geometry of the bound ligands. Moreover, the position of the *N*-methyl-l-prolinol group in the molecules excludes an important electronic effect on the reactivity. Indeed, we observed pairwise similarity of the calculated reaction barriers with differences within 1 kcal/mol except for **4a,b** where the difference amounts to 1.7 kcal/mol (Table 2).

The effect of warhead on the reactivity of the compounds is significant as it is shown by the important variation of the reaction barriers both within the **1a–4a** and **1b–4b** series of compounds. In fact, as the scaffold variation does not have an important effect on reactivity as it is discussed above, the **a** and **b** series can be treated together. Based on the magnitude of reaction barriers compounds **1a,b** and **2a,b** with acrylamide and chloroacetamide warhead, respectively, form one group, while compounds **3a,b** and **4a,b** with imidazole carboxamide and aziridine carboxamide warheads, respectively, form another group. Compounds within a group have similar barriers while the first group (**1a,b** and **2a,b**) have lower barriers than do the second group (**3a,b** and **4a,b**). It is worth noting that chloroacetamides show higher reactivity toward GSH than do acrylamides (Table 1), nevertheless, their *k*_inact_ values are similar. Although chloroacetamides are most often more active than acrylamides,^26,41,42^ the target and scaffold dependence of their relative reactivity has been previously observed (cf. Table 3 in ref (26) and data set 3 in the Supporting Information of ref (41)). The similar *k*_inact_ values may also indicate that both warheads are reactive enough and the complete chemical reaction in KRAS^G12C^ labeling includes a warhead independent rate limiting step, like e.g., the Cys12 thiol deprotonation. The lower *k*_inact_ of compounds **3a,b** and **4a,b** is attributed to the lower reactivity of their warheads at the pH of the reaction mixture (imidazole carboxamide and aziridine carboxamide) compared to compounds **1a,b** and **2a,b** (acrylamide and chloroacetamide). The reactivity of imidazole and aziridine containing warheads depends on the protonation state and increases with decreasing pH. The p*K*_a_ of the conjugated acids of free imidazole and aziridine is 7.0 and 8.0,^43^ respectively, and the warheads of **3a,b** and **4a,b** are expected to be partially protonated close to neutral pH, and this reduces their reactivity. The lower reactivity compared to **1a,b** and **2a,b** suggests that the rate-limiting step includes the warhead and the similar *k*_inact_ of **3a,b** and **4a,b** suggests higher reactivity of **3a,b** compared to **4a,b** as a lower concentration of the active protonated form is present in the former.

**Table 3:** Anti-Proliferative Activity and Selectivity Factors of Compounds **1**–**4a,b**

	1a	1b	2a	2b	3a	3b	4a	4b	ARS-1620
	IC_50_ [μM]								
LCLC-103H^a^	34.0	82.0	7.5	4.2	47.1	41.6	78.9	91.8	20.24
H1792^b^	16.2	30.1	2.9	1.8	17.9	17.7	22.6	30.8	10.19
MiaPaca2^b^	1.8	0.53	3.1	3.2	20.4	8.8	19.8	48.2	0.14
H358^b^	2.3	1.1	2.7	1.8	27.4	9.9	27.1	90.0	0.31
	selectivity factors toward KRAS^G12C^ in comparison to KRAS^wt^								
H1792^b^	2.1	2.7	2.6	2.3	2.6	2.4	3.5	3.0	1.9
MiaPaca2^b^	18.9	154.7	2.4	1.3	2.3	4.7	4.0	1.9	140.6
H358^b^	14.8	74.5	2.8	2.3	1.7	4.2	2.9	1.0	66.3

a KRAS^wt^ expressing cell.

b KRAS^G12C^ expressing cell.

Our compounds were also compared to fully optimized inhibitors against KRAS^G12C^ that reached clinical development.^44^ As we have not found published *k*_inact_ and *K*_I_ for these compounds except for Sotorasib (*k*_inact_ = 0.035 s^–1^, *K*_I_ = 12.2 μM),^45^ we collected clinically tested ARS-1620 (*k*_inact_/*K*_I_ = 1100 M^–1^ s^–1^)^36^ analogues with available *k*_inact_/*K*_I_: Divarasib (*k*_inact_/*K*_I_ = 710,000 M^–1^ s^–1^),^46^ Adagrasib (*k*_inact_/*K*_I_ = 35,000 M^–1^ s^–1^),^47^ D3S-001 (*k*_inact_/*K*_I_ = 158,000 M^–1^ s^–1^)^48^ Sotorasib (*k*_inact_/*K*_I_ = 2870 M^–1^ s^–1^).^45^ Data show that there is a large improvement of *k*_inact_/*K*_I_ between Sotorasib (2870), a first-in-class FDA-approved drug, and D3S-001 (1,580,000), a compound in phase I clinical trial having the largest *k*_inact_/*K*_I_ among the analogs. We suggest that a decrease in K_I_ is primarily responsible for the high *k*_inact_/*K*_I_ while there is no significant variation expected in *k*_inact_. We recall that the *K*_I_ values are higher than 10 μM for our compounds, ARS-1620^36^ and Sotorasib,^45^ while it was reported that the variation of the scaffold has the potency to significantly improve the noncovalent recognition.^49^ Appropriate substituents on the central fused rings and on the connected piperazine and aryl rings can lock torsion angles in the bioactive conformation while small substituents adjacent to the amide nitrogen of the piperazine can displace an unfavorable water molecule to increase noncovalent affinity. By contrast, *k*_inact_ is in the 10^–2^ s^–1^ range for compounds **1–2a,b** and this is similar to the highest value for a wide range of kinases,^50^ including BTK,^51^ JAK3,^52^ EGFR,^53^ and FGFR.^54,53^ These observations suggest that optimization of covalent ligands should focus on noncovalent scaffold optimization while keeping the reactivity in the required range.

The relative insensitivity of the noncovalent binding to both the scaffold and warhead variations and the significant effect of warhead on the reactivity are reflected in the IC_50_ values of both the nucleotide exchange assay (Table 1) and the cell proliferation assay (Table 3). Compound pairs in the **a** and **b** series exhibit similar IC_50_s, while **1a,b** and **2a,b** have lower IC_50_s compared to **3a,b** and **4a,b**. These data show that inhibition efficiency characterized by the *K*_I_ and *k*_inact_ values reproduce the tendency in activity variations in both in vitro assays. The observed KRAS^G12C^ versus KRAS^WT^ selectivity is cell-line-dependent and this is in accordance with the substantial variability of KRAS dependency among KRAS G12C mutant cell lines.^55^ Compound **1b** with acrylamide warhead and *N*-methyl-l-prolinol substituent exhibits outstanding selectivity comparable with that of ARS-1620. Compound **1a** without *N*-methylprolinol substituent exhibits lower, but still significant selectivity, while other compounds show modest selectivity (Table 3). Interestingly, all investigated compounds were slightly more selective against the H1792 mutant cell line than ARS-1620.

## Conclusions

The relative contribution of noncovalent recognition and reactivity of covalent inhibitors was investigated on KRAS^G12C^ with ARS analog compounds having four different warheads and two skeleton variations. NMR and MS/MS measurements showed that all synthesized compounds bind to Cys12 via a covalent bond, and it proves that the atropisomeric core is not indispensable for KRAS^G12C^ inhibition. Although the covalent bond formation reactions follow different mechanisms with unrevealed details, the commonly applied two-step covalent inhibition model was suitable for deriving parameters appropriate for interpreting variations in inhibition efficiency. We found that the investigated skeleton variation of ARS analogs does not have an important effect either on the noncovalent or the covalent binding step. This is at variance with the previously reported cell-based assay results and their interpretation; however, our consistent data from MS-based *K*_I_ and *k*_inact_ determination together with isolated enzyme and cell-based assays support the present findings. Computed noncovalent binding free energies are also in accordance with similar noncovalent affinities for all compounds. Investigated warhead variations do not have a significant effect on noncovalent binding but they affect the covalent binding step; compounds with acrylamide and chloroacetamide warheads exhibit more efficient covalent inhibition owing to higher reactivity than do compounds with aziridine carboxamide and imidazole carboxamide warheads. These differences are reflected in the biochemical assays where more reactive compounds exhibit significantly higher functional efficacy both in an isolated protein and a cell-based assay. Our results suggest that analyzing the contribution of noncovalent and covalent interactions might help focus the optimization efforts of covalent inhibitors to identify covalent agents with improved efficacy.

## Experimental Section

### Procedures

NMR measurements were performed on a System 500 NMR spectrometer (Varian, Palo Alto, CA, USA) or a Varian System 300 spectrometer. ^1^H- and ^13^C NMR spectra were measured at RT (25 °C) in an appropriate solvent. ^1^H and ^13^C chemical shifts are expressed in parts per million (δ) referenced to TMS or residual solvent signals. All chemicals and solvents were used as purchased. HPLC-MS measurements were performed using an LC-MS-2020 device (Shimadzu, Kyoto, Japan) equipped with a Reprospher 100 C18 (5 μm, 100 × 3 mm) column and positive–negative double ion source (DUIS±) with a quadrupole mass spectrometer in a range of 50–1000 *m*/*z*. Samples were eluted with gradient elution using eluent A (0.1% formic acid in water) and eluent B (0.1% formic acid in acetonitrile). The flow rate was set to 1.5 mL/min. The initial condition was 0% B eluent, followed by a linear gradient to 100% B eluent by 2 min, from 2 to 3.75 min 100% B eluent was retained, and from 3.75 to 4.5 min back to the initial condition and retained to 5 min. The column temperature was kept at 30 °C and the injection volume was 1 μL. High-resolution mass spectrometric measurements were performed using a Q-TOF Premier mass spectrometer (Waters, Milford, MA, USA) in positive electrospray ionization mode.

### Syntheses

#### 1-(4-(6-Chloro-7-(2-fluoro-6-hydroxyphenyl)quinazolin-4-yl)piperazin-1-yl)prop-2-en-1-one (**1a**)

**15a** (75 mg, 0.209 mmol) was dissolved in DCM, TEA (146 μL) was added, and the mixture was cooled to −50 °C. After the addition of acryloyl chloride (**16**) (51 μL, 0.63 mmol), the mixture was stirred for 1h at −40 °C. The mixture was diluted with DCM and washed with water. The organic phase was then dried over Na_2_SO_4_ and the solvent was evaporated. The product was then dissolved in THF:water 1:1 and LiOH (14.1 mg) was added followed by 1 h stirring at r.t. The pH was then adjusted to 7. EtOAc was added and the organic phase was washed with brine, then dried over Na_2_SO_4_. After removing the solvent, the crude product was purified with prep-HPLC, resulting in 4.7 mg (14%) product.

^1^H NMR (500 MHz, CD_3_OD) δ (ppm) 8.65 (s, 1H), 8.20 (s, 1H), 7.79 (s, 1H), 7.29 (dd, *J* = 15.0, 8.3 Hz, 1H), 6.86–6.76 (m, 2H), 6.72 (t, *J* = 8.7 Hz, 1H), 6.28 (dd, *J* = 16.8, 1.8 Hz, 1H), 5.81 (dd, *J* = 10.6, 1.8 Hz, 1H), 4.00 (s, 4H), 3.92 (d, *J* = 5.3 Hz, 4H).

^13^C NMR (500 MHz, CD_3_OD) δ (ppm) 170.4, 167.2, 163.4, 160.1, 157.7, 153.3, 141.5, 136.1 134.8, 134.1, 131.4, 131.3, 128.8,, 120.2, 114.9, 109.6, 109.5, 52.9, 52.6, 48.9.

Exact mass: calc.: 413.118, found: 413.1193.

#### 1-(4-(6-Chloro-7-(2-fluoro-6-hydroxyphenyl)-2-((1-methylpyrrolidin-2-yl)methoxy)quinazolin-4-yl)piperazin-1-yl)prop-2-en-1-one (**1b**)

The solution of **15b** (0.05 g, 0.106 mmol) in MeCN (2 mL) was cooled to 0 °C, and then, acryoyl chloride (**16**) (0.01 mL, 0.117 mmol) and DIPEA (0.037 mL, 0.212 mmol) were added. The reaction mixture was stirred overnight at RT. Distilled water was added and then extracted with EtOAc. The combined organic layers were dried over Na_2_SO_4_ and concentrated under a vacuum. The crude product was purified via prep-HPLC chromatography to afford the product as a white solid (5.7 mg, 10%).

^1^H NMR (500 MHz, CD_3_OD) δ (ppm) 1.75 (m, 1H), 1.83 (m, 2H), 2.12 (m, 1H), 2.37 (m, 1H), 2.53 (s, 3H), 2.79 (m, 1H), 3.10 (m, 1H), 3.92 (m, 4H), 3.98 (m, 4H), 4.42 (m, 1H), 4.48 (m, 1H), 5.81 (m, 1H), 6.23 (m, 1H), 6.69 (m, 1H), 6.75–6.85 (m, 2H), 7.26 (m, 1H), 7.57 (s, 1H), 8.11 (s, 1H).

^13^C NMR (500 MHz, CD_3_OD) δ (ppm) 174.07, 166.44, 165.51, 160.85, 159.40, 156.11, 156.06, 151.45, 137.80, 129.73, 127.61, 127.35, 127.28, 125.12, 113.53, 110.95, 110.93, 105.70, 67.48, 64.46, 56.84, 39.92, 26.21, 21.96, 19.53.

Exact mass: calc: 526.2015, found: 526.2035

#### (4-(6-Chloro-7-(2-fluoro-6-hydroxyphenyl)quinazolin-4-yl)piperazin-1-yl)(1*H*-imidazol-1-yl)methanone (**3a**)

**15a** (75 mg, 0.209 mmol) was dissolved in acetonitrile (5 mL) and cooled at 0 °C, and then, DIPEA (54 mg, 75 μL) and carbonyl diimidazole (**17**) (41 mg) were added. After 24 h stirring at r.t., water was added. The resulting precipitate was filtered and purified with prep-HPLC, yielding the product as a white solid (26 mg, 28%).

^1^H NMR (500 MHz, CD_3_OD) δ (ppm).

8.66 (s, 1H), 8.20 (s, 1H), 8.14 (s, 1H), 7.80 (s, 1H), 7.52 (d, *J* = 1.9 Hz, 1H), 7.29 (td, *J* = 8.3, 6.6 Hz, 1H), 7.12 (s, 1H), 6.78 (d, *J* = 8.3 Hz, 1H), 6.74–6.69 (m, 1H), 4.09–4.04 (m, 5H), 3.91–3.86 (m, 5H).

^13^C NMR (500 MHz, CD_3_OD) δ (ppm).

164.64, 162.80, 160.85, 157.58, 157.52, 155.18, 152.35, 150.75, 139.03, 133.71, 132.34, 131.61, 129.63, 126.16, 119.86, 117.65, 112.39, 106.96, 49.85, 46.89.

Exact mass: calc.: 451.1091, found: 451.1122.

#### (4-(6-Chloro-7-(2-fluoro-6-hydroxyphenyl)-2-((1-methylpyrrolidin-2-yl)methoxy)quinazolin-4-yl)piperazin-1-yl)(1*H*-imidazol-1-yl)methanone (**3b**)

The solution of **15b** (0.05 g, 0.106 mmol) in MeCN (2 mL) was cooled to 0 °C, then carbonyl diimidazole (**17**) (0.017 g, 0.106 mmol) and DIPEA (0.037 mL, 0.212 mmol) were added. The reaction mixture was stirred overnight at RT. Distilled water was added and then extracted with EtOAc. The combined organic layers were dried over Na_2_SO_4_ and concentrated under a vacuum. The crude product was purified via prep-HPLC chromatography to afford the product as a white solid (33.6 mg, 56%).

^1^H NMR (500 MHz, CD_3_OD) δ (ppm) 8.14 (s, 1H), 7.62 (s, 1H), 7.52 (s, 1H), 7.28 (td, *J* = 8.3, 6.6 Hz, 1H), 7.12 (s, 1H), 6.77 (d, *J* = 8.3 Hz, 1H), 6.70 (t, *J* = 8.7 Hz, 1H), 4.80–4.76 (m, 1H), 4.66–4.59 (m, 1H), 4.12–4.01 (m, 4H), 3.94–3.87 (m, 4H), 3.76–3.69 (m, 1H), 3.66–3.59 (m, 1H), 3.17–3.09 (m, 1H), 2.99 (s, 3H), 2.41–2.32 (m, 1H), 2.19–1.99 (m, 3H).

^13^C NMR (500 MHz, CD_3_OD) δ (ppm) δ 165.6, 161.3, 160.9, 159., 156.10, 156.0, 151.5, 151.0, 137.9, 130.1, 130.0, 129.8, 129.7, 128.3, 125.0, 113.8, 113.7, 113.5, 110.9, 105.7, 105.5, 67.4, 64.6, 56.9, 45.4, 40.0, 26.2, 22.0.

Exact mass: calc: 566.2082, found: 566.2099.

#### 2-Chloro-1-{4-[6-chloro-7-(2-fluoro-6-hydroxyphenyl)-quinazolin-4-il]piperazine-1-yl}ethane-1-one (**2a**)

To a solution of **15d** (R = Me) (300 mg, 0.805 mmol), PyAOP (500 mg, 0.966 mmol) and HOBt.H_2_O (120 mg, 0.889 mmol) were dissolved in dry acetonitrile (15 mL), followed by the addition of DIPEA (312 mg, 0.241 mmol, 420 μL). Chloroacetic acid (**20**) was added to the mixture dropwise (500 mg, 0.966 mmol), and then the reaction mixture was stirred at r.t. for 17 h. The solvent was diluted with EtOAc and washed with water. The organic phase was dried over Na_2_SO_4_, and then the solvent was evaporated under reduced pressure. The crude was purified via prep-HPLC yield: 307 mg (85%) white solid. The product (153 mg, 0.340 mmol) was dissolved in dry DCM (8 mL) under argon, and then, it was cooled to 0 °C. Boron tribromide in 1 mol/dm^3^ solution was added dropwise (2.1 mL, 2.1 mmol). The reaction was stirred at r.t. for 20 h and then it was poured into ice-cold water. The mixture was extracted with EtOAc, and the organic phase was washed with brine, dried over Na_2_SO_4_, and evaporated. The crude was purified by prep-HPLC, yielding 34.7 mg (44%) pure product.

^1^H NMR (500 MHz, CD_3_OD) δ (ppm) 8.65 (s, 1H), 8.19 (s, 1H), 7.78 (s, 1H), 7.32–7.26 (m, 1H), 6.78 (d, *J* = 8.3 Hz, 1H), 6.71 (t, *J* = 9.0 Hz, 1H), 4.34 (s, 2H), 4.04–4.00 (m, 2H), 3.99–3.95 (m, 2H), 3.87–3.82 (m, 4H).

^13^C NMR (500 MHz, CD_3_OD) δ (ppm) δ 170.8, 166.6, 163.3, 153.8, 149.3, 137.6, 132.2, 131.0, 130.2, 130.1, 124.8, 111.0,110.9,z 105.7, 105.6, 48.8, 48.6, 45.2, 41.7, 40.5.

Exact mass: calc.: 433.0639, found: 433.0665.

#### 2-Chloro-1-(4-(6-chloro-7-(2-fluoro-6-hydroxyphenyl)-2-((1-methylpyrrolidin-2-yl)methoxy)quinazolin-4-yl)piperazin-1-yl)ethan-1-one (**2b**)

To a solution of **15b** (0.05 g, 0.106 mmol) in THF (2 mL) pentafluorophenyl chloroacetate (**21**) (0.083 g, 0.312 mmol) was added. The reaction mixture was stirred for 5 min at RT. Distilled water was added and then extracted with EtOAc. The combined organic layers were dried over Na_2_SO_4_ and concentrated under a vacuum. The crude product was purified via prep-HPLC chromatography to afford the product as a white solid (15.4 mg, 27%).

^1^H NMR (500 MHz, CD_3_OD) δ (ppm) 8.19 (s, 1H), 7.63 (s, 1H), 7.29 (q, *J* = 7.9 Hz, 1H), 6.78 (d, *J* = 8.2 Hz, 1H), 6.71 (t, *J* = 8.7 Hz, 1H), 4.77–4.70 (m, 1H), 4.36 (s, 2H), 4.19–4.05 (m, 4H), 3.98 (s, 1H), 3.88 (s, 4H), 3.75 (s, 1H), 3.10 (s, 3H), 3.03–3.00 (m, 1H), 2.67–2.65 (m, 1H), 2.48–2.39 (m, 1H), 2.24–2.03 (m, 3H).

^13^C NMR (500 MHz, CD_3_OD) δ (ppm) 166.7, 165.2, 161.3, 159.4, 156.1, 156.0, 138.3, 130.2, 130.2, 125.5, 113.1, 111.0, 105.7, 105.5, 64.9, 57.0, 48.9, 48.4, 47.7, 47.5, 44.9, 41.5, 40.6, 26.2, 25.5, 22.1.

Exact mass: calc: 548.1631, found: 548.1640.

#### Aziridin-2-yl(4-(6-chloro-7-(2-fluoro-6-hydroxyphenyl)quinazolin-4-yl)piperazin-1-yl)methanone (**4a**)

**19a** (31 mg, 0.046 mmol) was dissolved in DCM (4 mL) and cooled to 0 °C. Triethylsilane (30 μL 0.19 mmol) and 2,2,2-trifluoroacetic acid (30 μL 0.37 mmol) were added, followed by 2 h stirring at r.t. DIPEA (80.6 μL, 0.46 mmol) was added and the mixture was stirred for 10 min, which was followed by addition of EtOAc and extraction with brine. The organic phase was dried over Na_2_SO_4_ and then evaporated. The mixture was purified with prep-HPLC, yielding 19.3 mg (18%) pure product.

^1^H NMR (500 MHz, CD_3_OD) δ (ppm) 8.66 (s, 1H), 8.21 (s, 1H), 7.79 (s, 1H), 7.32–7.26 (m, 1H), 6.78 (d, *J* = 8.3 Hz, 1H), 6.72 (t, *J* = 8.8 Hz, 1H), 4.10–3.95 (m, 7H), 3.90–3.85 (m, 2H), 1.89 (d, *J* = 12.6 Hz, 2H).

^13^C NMR (500 MHz, CD_3_OD) δ (ppm) 164.89, 163.45, 162.77, 160.83, 157.55, 154.87, 150.09, 139.24, 133.91, 131.88, 131.67, 126.20, 117.54, 112.38, 107.06 (J = 22.3 Hz), 51.26, 50.13, 49.34, 49.17, 48.83, 48.66, 48.49, 46.36, 40.91, 37.15, 30.87.Exact Mass: Calc.: 426.1138, Found: 426.1165

#### Aziridin-2-yl(4-(6-chloro-7-(2-fluoro-6-hydroxyphenyl)-2-((1-methylpyrrolidin-2-yl)methoxy)quinazolin-4-yl)piperazin-1-yl)methanone (**4b**)

The solution of **19b** (5 mg, 0.106 mmol) in DCM (2 mL) was cooled to 0 °C, then trifluoroacetic acid (65 mg, 0.847 mmol) and triethylsilane (67 μL, 0.424 mmol) were added. The reaction mixture was stirred for 2 h at RT. DIPEA (0,18 mL, 1.059 mmol) was added and then stirred for 15 min. The reaction mixture was washed with distilled water. The organic layer was dried over Na_2_SO_4_ and concentrated under a vacuum. The crude product was purified via prep-HPLC chromatography to afford the product as a white solid (33.6 mg, 56%).

^1^H NMR (500 MHz, CD_3_OD) δ (ppm) 8.43 (s, 1H), 8.17–8.12 (m, 1H), 7.61 (s, 1H), 7.28 (q, *J* = 7.8 Hz, 1H), 6.77 (d, *J* = 8.3 Hz, 1H), 6.71 (t, *J* = 8.8 Hz, 1H), 4.69–4.57 (m, 2H), 4.14–4.07 (m, 1H), 4.06–3.94 (m, 4H), 3.91–3.77 (m, 4H), 3.72–3.65 (m, 1H), 3.25–3.07 (m, 2H), 3.03 (s, 3H), 2.44–2.33 (m, 1H), 2.23–2.14 (m, 1H), 2.13–2.03 (m, 2H), 1.96–1.81 (m, 2H).

^13^C NMR (500 MHz, CD_3_OD) δ (ppm) 178.08, 173.65, 171.69, 169.55, 155.49, 141.72, 133.65, 133.48, 133.33, 129.01, 117.44, 114.87, 109.66, 109.48, 68.51, 68.20, 60.93, 52.39, 52.05, 51.88, 51.71, 51.54, 51.37, 51.20, 51.03, 46.37, 33.27, 32.84, 27.35, 26.24, 25.97, 16.94.

Exact mass: calc: 541.2124, found: 541.2138.

### Biological Evaluation

#### MANT-GDP Loading Assay

The assay described in ref (56) was followed with some modifications as described below. KRAS^G12C^ protein was first buffer-exchanged into low magnesium buffer (20 mM HEPES-NaOH (pH 7.5), 50 mM NaCl, 0.5 mM MgCl_2_) using a NAP5 column (catalog no.: 17-0583-1, Cytiva). The proteins were then incubated with 20-fold molar excess of *N*-methylanthraniloyl (MANT)-GDP (catalog no.: 69244, Sigma-Aldrich) in loading buffer (50 mM NaCl, 20 mM HEPES-NaOH [pH 7.5], 0.5 mM MgCl_2_, 10 mM EDTA, and 1 mM Dithiothreitol (DTT)) in a total volume of 200 μL at 20 °C for 90 min. The reaction was stopped by adding MgCl_2_ to a final concentration of 10 mM, then incubated at 20 °C for 30 min. The unbound MANT-GDP was removed using the NAP-5 column equilibrated with nucleotide exchange buffer (40 mM HEPES-NaOH (pH 7.5), 50 mM NaCl, 10 mM MgCl_2_, 2 mM Dithiothreitol DTT).

#### MANT-GDP Exchange Assay

The assay described in ref (56) was followed with some modifications as described below. First, the MANT-GDP bound KRAS^G12C^ protein (in a final 1 μM molar concentration) was incubated with the inhibitor molecules for 60 min. After the incubation, the MANT-GDP KRAS-inhibitor mixtures were loaded into a black 384-well microplate in the presence or absence of the different inhibitor molecules in various concentrations. The nucleotide exchange reaction was initiated by adding a 100-fold molar excess of GppNHp (catalog no.: G0635, Sigma-Aldrich), a nonhydrolyzable GTP analog, and the SOS1 exchange domain (catalog no.: GE02, Cytoskeleton, Inc.) protein in 0.2 μM final concentration. The change in fluorescence intensity was measured every 30 s in RT for 60 min on an EnvSpire plate reader (PerkinElmer, Inc.). The measured values were fitted to a single exponential function by using GraphPad Prism 10 software. The derived rates were normalized to the RAS-SOS1 minus RAS-only samples from which the IC_50_ values were calculated from three independent experiments with each inhibitor using GraphPad Prism 10 software.

#### Cell Proliferation Assay

##### Seeding of Cells

On the first day, lung cancer cell lines H358 (KRAS^G12C^), H1792 (KRAS^G12C^), LCLC-103H (KRAS^wt^), and pancreatic cancer cell line MiaPaca2 (KRAS^G12C^) were seeded in density 5000 cells per well in Roswell Park Memorial Institute medium (RPMI-1640; Biosera, Nuaille, France), supplemented with 5% Fetal Bovine Serum (FBS; Biosera), and 1% penicillin/streptomycin (Biosera).

##### Treatments

On the second day, after 24 h of incubation at 37 °C, cells were treated with various concentrations of **1–4a,b** and ARS-1620 from 20 mM DMSO stocks, and incubated at 37 °C for 72 h. Cells were treated with compounds at concentrations: 100 μM, 20 μM, 4 μM, 800 nM, 160 nM, and 32 nM (dilution factor 5×), dissolved in serum-free medium. The final concentration of serum was 2.5%, while the final concentration of DMSO was 0.5%. Control wells were treated with medium where the final concentration of serum was 2.5%, and DMSO 0.5%. Three replicates per concentration and 3 independent experiments were performed. Cell viability was determined by MTT assay, using 3-(4,5-dimethylthiazol-2-yl)-2,5-diphenyl-tetrazolium bromide obtained from Duchefa Biochemie (Haarlem, Netherlands) while IC_50_ was calculated using GraphPad Prism 6 Software (GraphPad, La Jolla, San Diego, CA, USA).

### HPLC-Based Reactivity Assay

HPLC-MS measurements were performed using a Shimadzu LCMS-2020 device equipped with a positive–negative double ion source (DUIS±) and a quadrupole MS analyzer in the range of *m*/*z* 50–1000. The sample was eluted with gradient elution using eluent A (0.1% FA in H2O) and eluent B (0.1% FA in ACN). The column temperature was always kept at 30 °C; the injection volume was 20 μL, the flow rate was set to 1.5 mL/min, and a Reprospher C18 (5 μm, 100 mm × 3 mm) column was used along with the following gradient. The initial condition was 0% B eluent, followed by a linear gradient to 100% B eluent by 1 min; from 1 to 3.5 min, 100% B eluent was retained. From 3.5 to 4.5 min, the initial condition with 5% B eluent was restored and retained until 5 min. For the reactivity and stability assay, a 250 μM solution of the fragment [in PBS buffer (pH 7.4) with 5% acetonitrile] with a 100 μM solution of indoprofen as the internal standard was incubated with or without 5 mM glutathione (providing results of reactivity or stability, respectively). The reaction mixture was analyzed by HPLC-MS sampling after 0, 1, 2, 4, 8, 12, 24, 48, and 72 h. The AUC (area under the curve) values were determined via the integration of HPLC chromatograms and then corrected with the internal standard. The fragments’ AUC values were subjected to ordinary least-squares (OLS) linear regression, and to compute the important parameters (kinetic rate constant and half-life time), an Excel sheet was applied. The data are expressed as means of duplicate determinations. The kinetic rate constant for the degradation and corrected GSH reactivity were calculated as follows. The reaction half-life for pseudo-first-order reactions (*t*_1/2_) is ln 2/*k*, where *k* is the reaction rate. In the case of competing reactions (reaction with GSH and degradation), the apparent reaction rate is *k*_app_ = *k*_deg_ + *k*_GSH_. When half-lives are measured experimentally, *t*_1/2(app)_ = ln 2/(*k*_app_) = ln 2/(*k*_deg_ + *k*_GSH_). In our case, the corrected *k*_deg_ and *k*_app_ (regarding blank and GSH-containing samples, respectively) can be calculated by the linear regression of the measured kinetic data points. The corrected *k*_GSH_ is calculated as *k*_app_ – *k*_deg_, and finally, the half-life is determined using the equation *t*_1/2_ = ln 2/*k*.

### LC-MS/MS Measurements

#### LC-MS Occupancy Measurements

MS-based occupancy analysis of the protein was performed on a Sciex Triple Quad 3500 LC-MS/MS system (Sciex, Framingham, U.S.A.) equipped with a Turbo V ion source in electrospray ionization mode. Chromatographic separation was performed on a Sciex Exion 2.0 HPLC system consisting of a binary pump, an autosampler, and a column compartment. Not a real chromatographic separation was carried out, samples were only retained on a short security guard cartridge (Phenomenex widepore C4, 4 × 3 mm) to focus and desalt the protein samples prior to ionization, the labeled and unlabeled proteins were not separated. A 4 min gradient (both in solvent composition and flow rate) was used with an initial flow of 0.5 mL/min and 10% eluent B. This was held for 0.5 min to wash out the salts from the sample. A 1.5 min linear increase was applied to reach the final flow of 1 mL/min and maximum eluent composition of B at 65%. These parameters were held for 0.5 min and a quick 0.1 min linear gradient was used to reach the initial flow rate and eluent composition. This was followed by a 1.4 min equilibrating part. Water containing 0.1% formic acid (eluent A) and acetonitrile containing 0.1% formic acid) were used for chromatography. The column temperature was kept at RT and the injection volume was 10 μL. The MS parameters were as follows: 450 °C source temperature, 5000 V ion spray voltage, and 120 V declustering potential. Compressed air was used as the nebulizer gas (GS1) and heater gas (GS2), and nitrogen was used as curtain gas with values set at 35, 45, and 45, respectively.

The protein was diluted to 100 μg/mL using pH 7.4 PBS buffer. Twenty microliters of the diluted protein were treated with 0.2 μL DMSO solutions of the compounds at concentrations of 1, 2, 4, 8, and 16 mM. The incubation times used for compounds **1–2a,b** were 0.5, 2, 5, 15, and 60 min, for compounds **3a** and **4b** 1, 2.5, 4.5, 8.5, and 24 h, and for compounds **3b** and **4a** 1, 2, 4.5, 17, and 24 h. The reactions were quenched with 2 μL 10% formic acid solution. Each experiment was measured at two biological parallels. The ratio of the labeling was determined from the ratio of the MS peak heights. After the identification of single occupancy levels in each experimental setup, those were plotted against time using GraphPad Prism Software, and based on experimental decay we calculated the *k*_obs_ (s^–1^). These values were then plotted against the concentration of the probe and *K*_I_ and *k*_inact_ were calculated directly from nonlinear regression according to the *k*_obs_ – *c* function as follows: .

LC-MS analysis: Intact protein analysis and peptide mapping were performed on a high-resolution hybrid quadrupole-time-of-flight mass spectrometer (Waters Select Series Cyclic IMS, Waters Corp., Wilmslow, U.K.). The mass spectrometer operated in positive V mode. Leucine enkephalin was used as the Lock Mass standard. Chromatographic separations were performed on a Waters Acquity I-Class UPLC system, coupled directly to the mass spectrometer.

#### Intact Reversed Phase Chromatography–Mass Spectrometry

RPLC-MS analysis of the intact proteins was performed on a Waters Acquity BEH300 C4 UPLC column (2.1 × 150 mm, 1.7 μm) under the following parameters: mobile phase “A”: 0.1% trifluoroacetic acid in water, mobile phase “B”: 0.1% trifluoroacetic acid in acetonitrile; flow rate: 400 μL/min; column temperature: 65 °C; gradient: 1 min: 5%B, 20 min: 50%B, 21 min: 90%B. UV detection was performed at 220 and 280 nm. The *m*/*z* range was 400–2000. Deconvolution was performed by the MaxEnt 1 software.

#### Peptide Mapping

Modification sites were determined by proteolysis and RPLC-MS peptide mapping. Briefly, proteins were enzymatically digested after buffer exchange using Amicon Ultra-0.5 mL Centrifugal Filter units (10 kDa, Merck Millipore). Trypsin-LysC mixture and ProAlanase (Promega Corporation, Madison, USA) were used for the enzymatic digestion. Briefly, protein samples were reduced by dithiothreitol at 37 °C for 30 min. After reduction, proteins were digested using a 1:50 enzyme:protein ratio. Overnight digestion was performed by Trypsin-LysC mixture in 50 mM NH_4_HCO_3_ solution at 37 °C. Tryptic digestion was stopped by adding formic acid in a final concentration of 0.2% (V/V). ProAlanase digestion was performed for 4 h at 37 °C in 50 mM HCl and was stopped by heating at 90 °C for 10 min. After digestions, an additional short incubation with dithiothreitol was repeated (5 min, 37 °C).

Gradient elution was performed on a Waters Acquity CSH Peptide C18 UPLC column (2.1 × 150 mm, 1.7 μm) under the following parameters: mobile phase “A”: 0.1% formic acid in water, mobile phase “B”: 0.1% formic acid in acetonitrile; flow rate: 300 μL/min; column temperature: 60 °C; gradient: 2 min: 2%B, 80 min: 45%B, 81 min: 85%B. MS^E^ experiments were performed using collision voltage ramping. MS data acquisition was performed under the following parameters: *m*/*z* 50–2000, scan time: 0.3 s, single Lock Mass: leucine enkephalin; low energy: 6 V, high energy: ramping 19–45 V. BiopharmaLynx 1.3.5 software (Waters Corp., Wilmslow, U.K.) was used to for data analysis.

### HMQC NMR Spectroscopy

NMR samples contained 50–70 μM ^15^N KRAS^G12C^ protein, 100–600 μM binding partner, 10 mM MgCl_2_, 3 mM NaN_3_, in PBS buffer, 5–10% DMSO-*d*_*6*_, 7% D_2_O, 5 μM DSS, pH 7.4. The samples were measured twice, immediately after the sample preparation and 1 day later, in between the two, they were stored at 4–8 °C. NMR spectra were acquired at 298 K on a Bruker AVANCE III spectrometer operating at 700.05 MHz, equipped with a 5 mm Prodigy TCI H&F-C/N-D, z-gradient probe head. The temperature was calibrated with a standard methanol solution. The chemical shifts were referenced with respect to the ^1^H-resonance of the internal DSS standard, while ^15^N chemical shifts were referenced indirectly via the gyromagnetic ratios according to the IUPAC conventions. All NMR data were processed with TopSpin and analyzed in NMRFAM-SPARKY. We used our former peak list of the free KRAS^G12C^ protein for the NMR signal assignment.^22^

### In Silico Docking

#### Induced Fit Docking

X-ray structure of KRAS^G12C^ in complex with ARS 1620 (PDBID: 5V9U) was used to perform the docking simulations. Protein was prepared with Protein Preparation Wizard (Schrödinger Release 2021-3) using default methods. Ligands were prepared with Schrödinger Ligprep using default methods. Docking was performed with Schrödinger Induced Fit Docking,^57−59^ generating 20 possible binding conformations. Redocking was done into structures within 30 kcal/mol of the best structure, and within the top 20 structures overall using a single precision method. Proteins with the best ligand binding conformations were selected for covalent docking.

#### Covalent Docking

CovDock^60^ of Schrödinger (Schrödinger Release 2021-3) was used to perform the covalent docking. Proteins were prepared as described previously. After choosing the appropriate reaction type, default settings were used.

### Computation of the Equilibrium Binding Constant

#### Thermodynamic Integration

Thermodynamic integration was carried out using the pmemd and pmemd.CUDA modules of the AMBER^61^ software package. The transformations were performed using the dual topology model applying the FF14SB^62^ force field. Each transformation step consisted of three parallel simulations, the final ΔΔ*G*s are reported as the average of the parallel ΔΔ*G*s along with the standard deviations. The starting structure of the protein was extracted from the above-mentioned ARS-1620–KRAS^G12C^ complex (PDBID: 5V9U). Ligand structures were constructed based on the crystallographic pose of ARS-1620 and modified into the respective molecules. The protein–ligand systems were constructed with the tLeap module of AMBERtools, combining the protein, the reference, and perturbed ligand structures for the protein complex and the two ligands for the solvated ligands. The complex and ligand systems were immersed into an octahedral TIP3P^63^ waterbox with a spacing of 12 Å and a closeness parameter set to 1.0. The constructed systems were minimized using 1000 steps of steepest descent minimization, which was followed by 20 ps of *NVT* heating to 300 K and 200 ps of *NPT* equilibration. All steps were carried out at λ = 0.5, applying softcore potential for the perturbed atoms (ifsc = 1). After system preparation, productive MDs were carried out from λ = 0 to λ = 1 by 0.1 increments in three steps: (i) a decharging step, where the charges of the softcore region’s atoms are deleted; (ii) a van der Waals step, where the vdW radii of reference molecules softcore region are transformed into those of the perturbed ones; and (iii) a recharging step, where the charges are reintroduced into the perturbed structure’s softcore region. The productive MDs are constructed by 3 × 11 = 33 windows per transformation, each consisting of a 20 ps *NVT* heating step and a 200 ps *NVT* MD with the logdvdl parameter set to 1. The ΔΔ*G* values were calculated using the analyze.sh and getdvdl.py scripts, available at AMBER’s Web site. The calculated ΔΔ*G* values are transformed into Δ*G* values using ligand **1a** as a reference molecule with its Δ*G* set to 0 kcal/mol. In the case of the **1a** → **1b** transformation the appearing additional positive charge on the protonated heterocyclic ring of **1b** is neutralized by a dummy chlorine ion placed in the TI region of the perturbed ligand. Temperature and pressure regulation were carried out by the Langevin thermostat with a collision frequency of 2 ps^–1^ and with the Berendsen barostat, applying 2 ps relaxation time, respectively. The softcore regions and ligand transformation steps are shown in Figure S5. The individual ΔΔ*G* values of the given transformation steps are shown in Table S3.

## Supporting Information Available

Additional experimental and computational details, compound characterization, NMR spectra (PDF)

## References

[ref1] Boike L.; Henning N. J.; Nomura D. K. Advances in Covalent Drug Discovery. Nat. Rev. Drug Discovery 2022, 21 (12), 881–898. 10.1038/s41573-022-00542-z. 36008483 PMC9403961

[ref2] Gehringer M.; Laufer S. A. Emerging and Re-Emerging Warheads for Targeted Covalent Inhibitors: Applications in Medicinal Chemistry and Chemical Biology. J. Med. Chem. 2019, 62 (12), 5673–5724. 10.1021/acs.jmedchem.8b01153. 30565923

[ref3] Schaefer D.; Cheng X. Recent Advances in Covalent Drug Discovery. Pharmaceuticals 2023, 16 (5), 663 10.3390/ph16050663. 37242447 PMC10220821

[ref4] Copeland R. A. Evaluation of Enzyme Inhibitors in Drug Discovery: A Guide for Medicinal Chemists and Pharmacologists, 2nd edition; John Wiley & Sons, 2013. 16350889

[ref5] Strelow J. M. A Perspective on the Kinetics of Covalent and Irreversible Inhibition. SLAS Discovery 2017, 22 (1), 3–20. 10.1177/1087057116671509. 27703080

[ref6] Downward J. Regulatory Mechanisms Forras Proteins. BioEssays 1992, 14 (3), 177–184. 10.1002/bies.950140308. 1586371

[ref7] Milburn M. v; Tong L.; Abraham M.; Brünger A.; Nishimura S.; Kim S.; Milburn M. V.; Tong L.; Devos A. M.; Brunger A.; Yamaizumi Z.; Nishimura S.; Kim S. Switch Molecular Signal for Transduction: Structural Differences Betwveen Active and Inactive Forms of Protooncogenic Ras Proteins. Science 1990, 247 (4945), 939–945. 10.1126/science.2406906. 2406906

[ref8] Prior I. A.; Lewis P. D.; Mattos C. A Comprehensive Survey of Ras Mutations in Cancer. Cancer Res. 2012, 72 (10), 2457–2467. 10.1158/0008-5472.CAN-11-2612. 22589270 PMC3354961

[ref9] Hobbs G. A.; Der C. J.; Rossman K. L. RAS Isoforms and Mutations in Cancer at a Glance. J. Cell Sci. 2016, 129 (7), 1287–1292. 10.1242/jcs.182873. 26985062 PMC4869631

[ref10] Zhao H.; Li L.; Liu J.; Mai R.; Chen J.; Chen J. Discovery of ARS-1620 Analogs as KRas G12C Inhibitors with High in Vivo Antitumor Activity. Bioorg Chem. 2022, 121, 105652 10.1016/j.bioorg.2022.105652. 35158284

[ref11] Johnson L.; Greenbaum D.; Cichowski K.; Mercer K.; Murphy E.; Schmitt E.; Bronson R. T.; Umanoff H.; Edelmann W.; Kucherlapati R.; Jacks T. K-Ras Is an Essential Gene in the Mouse with Partial Functional Overlap with N-Ras. Genes Dev. 1997, 11 (19), 2468–2481. 10.1101/gad.11.19.2468. 9334313 PMC316567

[ref12] Ostrem J. M.; Peters U.; Sos M. L.; Wells J. A.; Shokat K. M. K-Ras(G12C) Inhibitors Allosterically Control GTP Affinity and Effector Interactions. Nature 2013, 503, 548 10.1038/nature12796. 24256730 PMC4274051

[ref13] Wang C.; Fakih M. Targeting KRAS in Colorectal Cancer. Curr. Oncol. Rep. 2021, 23 (3), 28 10.1007/S11912-021-01022-0. 33582927

[ref14] Janes M. R.; Zhang J.; Li L. S.; Hansen R.; Peters U.; Guo X.; Chen Y.; Babbar A.; Firdaus S. J.; Darjania L.; Feng J.; Chen J. H.; Li S.; Li S.; Long Y. O.; Thach C.; Liu Y.; Zarieh A.; Ely T.; Kucharski J. M.; Kessler L. V.; Wu T.; Yu K.; Wang Y.; Yao Y.; Deng X.; Zarrinkar P. P.; Brehmer D.; Dhanak D.; Lorenzi M. V.; Hu-Lowe D.; Patricelli M. P.; Ren P.; Liu Y. Targeting KRAS Mutant Cancers with a Covalent G12C-Specific Inhibitor. Cell 2018, 172 (3), 578–589. 10.1016/J.CELL.2018.01.006. 29373830

[ref15] Orgován Z.; Keserű G. M. Small Molecule Inhibitors of RAS Proteins with Oncogenic Mutations. Cancer Metastasis Rev. 2020, 39, 1107–1126. 10.1007/s10555-020-09911-9. 32770300 PMC7680341

[ref16] Li L.; Zhao H.; Liao H.; Chen J.; Liu J.; Chen J. Discovery of Novel Quinazoline-Based Covalent Inhibitors of KRAS G12C with Various Cysteine-Targeting Warheads as Potential Anticancer Agents. Bioorg Chem. 2021, 110, 104825 10.1016/j.bioorg.2021.104825. 33774492

[ref17] Lee C. U.; Grossmann T. N. Reversible Covalent Inhibition of a Protein Target. Angewandte Chemie - International Edition 2012, 51 (35), 8699–8700. 10.1002/anie.201203341. 22806944

[ref18] Kwan A. K.; Piazza G. A.; Keeton A. B.; Leite C. A. The Path to the Clinic: A Comprehensive Review on Direct KRASG12C Inhibitors. Journal of Experimental & Clinical Cancer Research 2022, 41 (1), 1–23. 10.1186/s13046-021-02225-w. 35045886 PMC8767686

[ref19] Jiang Z.; Li Y.; Zhou X.; Wen J.; Zheng P.; Zhu W. Research Progress on Small Molecule Inhibitors Targeting KRAS G12C with Acrylamide Structure and the Strategies for Solving KRAS Inhibitor Resistance. Bioorg. Med. Chem. 2024, 100, 117627 10.1016/j.bmc.2024.117627. 38310752

[ref20] McGregor L. M.; Jenkins M. L.; Kerwin C.; Burke J. E.; Shokat K. M. Expanding the Scope of Electrophiles Capable of Targeting K-Ras Oncogenes. Biochemistry 2017, 56 (25), 3178–3183. 10.1021/acs.biochem.7b00271. 28621541 PMC5665167

[ref21] Fell J. B.; Fischer J. P.; Baer B. R.; Ballard J.; Blake J. F.; Bouhana K.; Brandhuber B. J.; Briere D. M.; Burgess L. E.; Burkard M. R.; Chiang H.; Chicarelli M. J.; Davidson K.; Gaudino J. J.; Hallin J.; Hanson L.; Hee K.; Hicken E. J.; Hinklin R. J.; Marx M. A.; Mejia M. J.; Olson P.; Savechenkov P.; Sudhakar N.; Tang T. P.; Vigers G. P.; Zecca H.; Christensen J. G. Discovery of Tetrahydropyridopyrimidines as Irreversible Covalent Inhibitors of KRAS-G12C with in Vivo Activity. ACS Med. Chem. Lett. 2018, 9 (12), 1230–1234. 10.1021/acsmedchemlett.8b00382. 30613331 PMC6295846

[ref22] Pálfy G.; Vida I.; Perczel A. 1H, 15N Backbone Assignment and Comparative Analysis of the Wild Type and G12C, G12D, G12V Mutants of K-Ras Bound to GDP at Physiological PH. Biomol NMR Assign 2020, 14 (1), 1–7. 10.1007/s12104-019-09909-7. 31468366 PMC7069925

[ref23] Lito P.; Solomon M.; Li L. S.; Hansen R.; Rosen N. Cancer Therapeutics: Allele-Specific Inhibitors Inactivate Mutant KRAS G12C by a Trapping Mechanism. Science (1979) 2016, 351 (6273), 604–608. 10.1126/science.aad6204. PMC4955282 26841430

[ref24] Schrödinger Release 2023–4: Maestro; Schrödinger, LLC, New York, NY, 2023.

[ref25] Petri L.; Ábrányi-Balogh P.; Varga P. R.; Imre T.; Keserű G. M. Comparative Reactivity Analysis of Small-Molecule Thiol Surrogates. Bioorg. Med. Chem. 2020, 28 (7), 115357 10.1016/j.bmc.2020.115357. 32081630

[ref26] Ábrányi-Balogh P.; Petri L.; Imre T.; Szijj P.; Scarpino A.; Hrast M.; Mitrović A.; Fonovič U. P. U. P.; Németh K.; Barreteau H.; Roper D. I. D. I.; Horváti K.; Ferenczy G. G. G. G.; Kos J.; Ilaš J.; Gobec S.; Keserű G. M. G. M. A Road Map for Prioritizing Warheads for Cysteine Targeting Covalent Inhibitors. Eur. J. Med. Chem. 2018, 160, 94–107. 10.1016/j.ejmech.2018.10.010. 30321804

[ref27] Helten H.; Schirmeister T.; Engels B. Model Calculations about the Influence of Protic Environments on the Alkylation Step of Epoxide, Aziridine, and Thiirane Based Cysteine Protease Inhibitors. J. Phys. Chem. A 2004, 108 (38), 7691–7701. 10.1021/jp048784g.

[ref28] Vicik R.; Helten H.; Schirmeister T.; Engels B. Rational Design of Aziridine-Containing Cysteine Protease Inhibitors with Improved Potency: Studies on Inhibition Mechanism. ChemMedChem. 2006, 1 (9), 1021–1028. 10.1002/cmdc.200600081. 16933238

[ref29] Wade A. D.; Bhati A. P.; Wan S.; Coveney P. V. Alchemical Free Energy Estimators and Molecular Dynamics Engines: Accuracy, Precision, and Reproducibility. J. Chem. Theory Comput 2022, 18 (6), 3972–3987. 10.1021/acs.jctc.2c00114. 35609233 PMC9202356

[ref30] Wan S.; Tresadern G.; Pérez-Benito L.; van Vlijmen H.; Coveney P. V. Accuracy and Precision of Alchemical Relative Free Energy Predictions with and without Replica-Exchange. Adv. Theory Simul 2020, 3 (1), 1900195 10.1002/adts.201900195. 34527855 PMC8427472

[ref31] Mason P. E.; Jungwirth P.; Duboué-Dijon E. Quantifying the Strength of a Salt Bridge by Neutron Scattering and Molecular Dynamics. J. Phys. Chem. Lett. 2019, 10 (12), 3254–3259. 10.1021/acs.jpclett.9b01309. 31125523

[ref32] Ahmed M. C.; Papaleo E.; Lindorff-Larsen K. How Well Do Force Fields Capture the Strength of Salt Bridges in Proteins?. PeerJ. 2018, 6, e4967 10.7717/PEERJ.4967. 29910983 PMC6001725

[ref33] Capoferri L.; Lodola A.; Rivara S.; Mor M. Quantum Mechanics/Molecular Mechanics Modeling of Covalent Addition between EGFR-Cysteine 797 and N -(4-Anilinoquinazolin-6-Yl) Acrylamide. J. Chem. Inf Model 2015, 55 (3), 589–599. 10.1021/ci500720e. 25658136

[ref34] Silva J. R. A.; Cianni L.; Araujo D.; Batista P. H. J.; De Vita D.; Rosini F.; Leitão A.; Lameira J.; Montanari C. A. Assessment of the Cruzain Cysteine Protease Reversible and Irreversible Covalent Inhibition Mechanism. J. Chem. Inf Model 2020, 60 (3), 1666–1677. 10.1021/acs.jcim.9b01138. 32126170

[ref35] Martí S.; Arafet K.; Lodola A.; Mulholland A. J.; Świderek K.; Moliner V. Impact of Warhead Modulations on the Covalent Inhibition of SARS-CoV-2 Mpro Explored by QM/MM Simulations. ACS Catal. 2022, 12 (1), 698–708. 10.1021/acscatal.1c04661. 35036042

[ref36] Hansen R.; Peters U.; Babbar A.; Chen Y.; Feng J.; Janes M. R.; Li L. S.; Ren P.; Liu Y.; Zarrinkar P. P. The Reactivity-Driven Biochemical Mechanism of Covalent KRAS G12C Inhibitors. Nat. Struct Mol. Biol. 2018, 25 (6), 454–462. 10.1038/s41594-018-0061-5. 29760531

[ref37] Huynh M. V.; Parsonage D.; Forshaw T. E.; Chirasani V. R.; Hobbs G. A.; Wu H.; Lee J.; Furdui C. M.; Poole L. B.; Campbell S. L. Oncogenic KRAS G12C: Kinetic and Redox Characterization of Covalent Inhibition. J. Biol. Chem. 2022, 298 (8), 102186 10.1016/j.jbc.2022.102186. 35753348 PMC9352912

[ref38] Khrenova M. G.; Kulakova A. M.; Nemukhin A. V. Proof of Concept for Poor Inhibitor Binding and Efficient Formation of Covalent Adducts of KRASG12C and ARS Compounds. Org. Biomol Chem. 2020, 18 (16), 3069–3081. 10.1039/D0OB00071J. 32101243

[ref39] Mihalovits L. M.; Ferenczy G. G.; Keserű G. M. Free Energy Calculations in Covalent Drug Design. Computational Drug Discovery 2024, 561–578. 10.1002/9783527840748.ch23.

[ref40] Mihalovits L. M.; Ferenczy G. G.; Keserű G. M. The Role of Quantum Chemistry in Covalent Inhibitor Design. Int. J. Quantum Chem. 2022, 122, 26768 10.1002/qua.26768.

[ref41] Resnick E.; Bradley A.; Gan J.; Douangamath A.; Krojer T.; Sethi R.; Geurink P. P.; Aimon A.; Amitai G.; Bellini D.; Bennett J.; Fairhead M.; Fedorov O.; Gabizon R.; Gan J.; Guo J.; Plotnikov A.; Reznik N.; Ruda G. F.; Díaz-Sáez L.; Straub V. M.; Szommer T.; Velupillai S.; Zaidman D.; Zhang Y.; Coker A. R.; Dowson C. G.; Barr H. M.; Wang C.; Huber K. V. M.; Brennan P. E.; Ovaa H.; Von Delft F.; London N. Rapid Covalent-Probe Discovery by Electrophile-Fragment Screening. J. Am. Chem. Soc. 2019, 141 (22), 8951–8968. 10.1021/jacs.9b02822. 31060360 PMC6556873

[ref42] Crowley V. M.; Thielert M.; Cravatt B. F. Functionalized Scout Fragments for Site-Specific Covalent Ligand Discovery and Optimization. ACS Cent Sci. 2021, 7 (4), 613–623. 10.1021/acscentsci.0c01336. 34056091 PMC8155467

[ref43] PubChem. https://pubchem.ncbi.nlm.nih.gov/ (accessed February 2024).

[ref44] Wang H.; Chi L.; Yu F.; Dai H.; Gao C.; Si X.; Wang Z.; Liu L.; Zheng J.; Shan L.; Liu H.; Zhang Q. Annual Review of KRAS Inhibitors in 2022. Eur. J. Med. Chem. 2023, 249, 115124 10.1016/j.ejmech.2023.115124. 36680986

[ref45] Bröker J.; Waterson A. G.; Smethurst C.; Kessler D.; Böttcher J.; Mayer M.; Gmaschitz G.; Phan J.; Little A.; Abbott J. R.; Sun Q.; Gmachl M.; Rudolph D.; Arnhof H.; Rumpel K.; Savarese F.; Gerstberger T.; Mischerikow N.; Treu M.; Herdeis L.; Wunberg T.; Gollner A.; Weinstabl H.; Mantoulidis A.; Krämer O.; McConnell D. B.; Fesik S. W. Fragment Optimization of Reversible Binding to the Switch II Pocket on KRAS Leads to a Potent, in Vivo Active KRASG12CInhibitor. J. Med. Chem. 2022, 65 (21), 14614–14629. 10.1021/ACS.JMEDCHEM.2C01120. 36300829 PMC9661478

[ref46] Purkey H. Abstract ND11: Discovery of GDC-6036, a Clinical Stage Treatment for KRAS G12C-Positive Cancers. Cancer Res. 2022, 82 (12_Supplement), ND11 10.1158/1538-7445.AM2022-ND11.

[ref47] Fell J. B.; Fischer J. P.; Baer B. R.; Blake J. F.; Bouhana K.; Briere D. M.; Brown K. D.; Burgess L. E.; Burns A. C.; Burkard M. R.; Chiang H.; Chicarelli M. J.; Cook A. W.; Gaudino J. J.; Hallin J.; Hanson L.; Hartley D. P.; Hicken E. J.; Hingorani G. P.; Hinklin R. J.; Mejia M. J.; Olson P.; Otten J. N.; Rhodes S. P.; Rodriguez M. E.; Savechenkov P.; Smith D. J.; Sudhakar N.; Sullivan F. X.; Tang T. P.; Vigers G. P.; Wollenberg L.; Christensen J. G.; Marx M. A. Identification of the Clinical Development Candidate MRTX849, a Covalent KRASG12CInhibitor for the Treatment of Cancer. J. Med. Chem. 2020, 63 (13), 6679–6693. 10.1021/acs.jmedchem.9b02052. 32250617

[ref48] Zhang J.; Wu W.; Xu Y.; Qian Y.; Xia Y.; Lu J.; Zheng Z.; Lu J.; Chen J.; Chen C.; Wang J.; Chen C.; Rui H.; Wang A.; Jin J.; Chen Z. J. Preclinical Characterization of D3S-001, a Highly Potent, Selective, and Differentiated Covalent Inhibitor of KRAS G12C. Journal of Clinical Oncology 2022, 40 (16_suppl), e15102 10.1200/JCO.2022.40.16_SUPPL.E15102.

[ref49] Bodnarchuk M. S.; Cassar D. J.; Kettle J. G.; Robb G.; Ward R. A. Drugging the Undruggable: A Computational Chemist’s View of KRASG12C. RSC Med. Chem. 2021, 12 (4), 609–614. 10.1039/D1MD00055A. 34046632 PMC8128063

[ref50] Abdeldayem A.; Raouf Y. S.; Constantinescu S. N.; Moriggl R.; Gunning P. T. Advances in Covalent Kinase Inhibitors. Chem. Soc. Rev. 2020, 49 (9), 2617–2687. 10.1039/C9CS00720B. 32227030

[ref51] Hopper M.; Gururaja T.; Kinoshita T.; Dean J. P.; Hill R. J.; Mongan A. Relative Selectivity of Covalent Inhibitors Requires Assessment of Inactivation Kinetics and Cellular Occupancy: A Case Study of Ibrutinib and Acalabrutinib. J. Pharmacol. Exp. Ther. 2020, 372 (3), 331–338. 10.1124/jpet.119.262063. 31871305

[ref52] Elwood F.; Witter D. J.; Piesvaux J.; Kraybill B.; Bays N.; Alpert C.; Goldenblatt P.; Qu Y.; Ivanovska I.; Lee H. H.; Chiu C. S.; Tang H.; Scott M. E.; Deshmukh S. V.; Zielstorff M.; Byford A.; Chakravarthy K.; Dorosh L.; Rivkin A.; Klappenbach J.; Pan B. S.; Kariv I.; Dinsmore C.; Slipetz D.; Dandliker P. J. Evaluation of JAK3 Biology in Autoimmune Disease Using a Highly Selective, Irreversible JAK3 Inhibitor. Journal of Pharmacology and Experimental Therapeutics 2017, 361 (2), 229–244. 10.1124/jpet.116.239723. 28193636

[ref53] Engel J.; Richters A.; Getlik M.; Tomassi S.; Keul M.; Termathe M.; Lategahn J.; Becker C.; Mayer-Wrangowski S.; Grütter C.; Uhlenbrock N.; Krüll J.; Schaumann N.; Eppmann S.; Kibies P.; Hoffgaard F.; Heil J.; Menninger S.; Ortiz-Cuaran S.; Heuckmann J. M.; Tinnefeld V.; Zahedi R. P.; Sos M. L.; Schultz-Fademrecht C.; Thomas R. K.; Kast S. M.; Rauh D. Targeting Drug Resistance in EGFR with Covalent Inhibitors: A Structure-Based Design Approach. J. Med. Chem. 2015, 58 (17), 6844–6863. 10.1021/acs.jmedchem.5b01082. 26275028

[ref54] Brameld K. A.; Owens T. D.; Verner E.; Venetsanakos E.; Bradshaw J. M.; Phan V. T.; Tam D.; Leung K.; Shu J.; Lastant J.; Loughhead D. G.; Ton T.; Karr D. E.; Gerritsen M. E.; Goldstein D. M.; Funk J. O. Discovery of the Irreversible Covalent FGFR Inhibitor 8-(3-(4-Acryloylpiperazin-1-Yl)Propyl)-6-(2,6-Dichloro-3,5-Dimethoxyphenyl)-2-(Methylamino)Pyrido[2,3-d]Pyrimidin-7(8H)-One (PRN1371) for the Treatment of Solid Tumors. J. Med. Chem. 2017, 60 (15), 6516–6527. 10.1021/acs.jmedchem.7b00360. 28665128

[ref55] Misale S.; Fatherree J. P.; Cortez E.; Li C.; Bilton S.; Timonina D.; Myers D. T.; Lee D.; Gomez-Caraballo M.; Greenberg M.; Nangia V.; Greninger P.; Egan R. K.; McClanaghan J.; Stein G. T.; Murchie E.; Zarrinkar P. P.; Janes M. R.; Li L. S.; Liu Y.; Hata A. N.; Benes C. H. KRAS G12C NSCLC Models Are Sensitive to Direct Targeting of KRAS in Combination with PI3K Inhibition. Clin. Cancer Res. 2019, 25 (2), 796–807. 10.1158/1078-0432.CCR-18-0368. 30327306

[ref56] Kanie T.; Abbott K. L.; Mooney N. A.; Plowey E. D.; Demeter J.; Jackson P. K. The CEP19-RABL2 GTPase Complex Binds IFT-B to Initiate Intraflagellar Transport at the Ciliary Base. Dev Cell 2017, 42 (1), 22–36. 10.1016/J.DEVCEL.2017.05.016. 28625565 PMC5556974

[ref57] Sherman W.; Day T.; Jacobson M. P.; Friesner R. A.; Farid R. Novel Procedure for Modeling Ligand/Receptor Induced Fit Effects. J. Med. Chem. 2006, 49 (2), 534–553. 10.1021/jm050540c. 16420040

[ref58] Farid R.; Day T.; Friesner R. A.; Pearlstein R. A. New Insights about HERG Blockade Obtained from Protein Modeling, Potential Energy Mapping, and Docking Studies. Bioorg. Med. Chem. 2006, 14 (9), 3160–3173. 10.1016/j.bmc.2005.12.032. 16413785

[ref59] Sherman W.; Beard H. S.; Farid R. Use of an Induced Fit Receptor Structure in Virtual Screening. Chem. Biol. Drug Des 2006, 67 (1), 83–84. 10.1111/j.1747-0285.2005.00327.x. 16492153

[ref60] Zhu K.; Borrelli K. W.; Greenwood J. R.; Day T.; Abel R.; Farid R. S.; Harder E. Docking Covalent Inhibitors: A Parameter Free Approach To Pose Prediction and Scoring. J. Chem. Inf Model 2014, 54 (7), 1932–1940. 10.1021/ci500118s. 24916536

[ref61] Case D. A.; Ben-Shalom I. Y.; Brozell S. R.; Cerutti D. S.; Cheatham T.E. I.; Cruzeiro V. W. D.; Darden T. A.; Duke R. E.; Ghoreishi D.; Gilson M. K.; Gohlke H.; Goetz A. W.; Greene D.; Harris R.; Homeyer N.; Izadi S.; Kovalenko A.; Kurtzman T.; Lee T. S.; LeGra S.; Kollman P. A. AMBER 2020; University of California: San Francisco, 2020.

[ref62] Maier J. A.; Martinez C.; Kasavajhala K.; Wickstrom L.; Hauser K. E.; Simmerling C. Ff14SB: Improving the Accuracy of Protein Side Chain and Backbone Parameters from Ff99SB. J. Chem. Theory Comput 2015, 11 (8), 3696–3713. 10.1021/acs.jctc.5b00255. 26574453 PMC4821407

[ref63] Jorgensen W. L.; Chandrasekhar J. M.; Madura J.; Impey W. R.; Klein M. L. Comparison of Simple Potential Functions for Simulating Liquid Water. J. Chem. Phys. 1983, 79 (2), 926–935. 10.1063/1.445869.

