# Supplementary material for: Contribution of Noncovalent Recognition and Reactivity to the Optimization of Covalent Inhibitors: A Case Study on KRasG12C
Source: ACS Chem Biol. 2024 Jul 11;19(8):1743–56. doi: 10.1021/acschembio.4c00217 (PMC11334105; doi:10.1021/acschembio.4c00217)
Supplement: Supplementary file 1 — cb4c00217_si_001.pdf [file cb4c00217_si_001.pdf]

SUPPORTING INFORMATION

Contribution of non-covalent recognition and reactivity to the optimization of covalent inhibitors: a case study on $KRas^{G12C}$

Nikolett Péczka[a,b#], Ivan Ranđelović[c#], Zoltán Orgován[a], Noémi Csorba[a,b], Attila Egyed[a], László Petri[a], Péter Ábrányi-Balogh[a], Márton Gadanecz[c,d], András Perczel[c], József Tóvári[e], Gitta Schlosser[f], Tamás Takács[g,h], Levente M. Mihalovits[a], György G. Ferenczy[a], László Buday[g], György M. Keserű[a,b]*

[a]HUN-REN Research Centre for Natural Sciences, Medicinal Chemistry Research Group and National Drug Discovery and Development Laboratory, 1117 Budapest, Hungary

[b]Budapest University of Technology and Economics, Department of Organic Chemistry and Technology, 1111 Budapest, Hungary

[c]ELTE Institute of Chemistry, Protein Modeling Research Group, Laboratory of Structural Chemistry and Biology, 1117 Budapest, Hungary

[d]Hevesy György PhD School of Chemistry, Eötvös Loránd University, Pázmány Péter sétány. 1/A, H-1117 Budapest, Hungary

[e]National Institute of Oncology, Department of Experimental Pharmacology and the National Tumor Biology Laboratory, 1122 Budapest, Hungary

[f]MTA-ELTE „Lendület" Ion Mobility Mass Spectrometry Research Group, 1117 Budapest, Hungary

[g]HUN-REN Research Centre for Natural Sciences, Signal Transduction and Functional Genomics Research Group, 1117 Budapest, Hungary

[h]Doctoral School of Biology, Institute of Biology, ELTE Eötvös Loránd University, 1117 Budapest, Hungary

corresponding author: György M. Keserű keseru.gyorgy@ttk.hu

#Note: Nikolett Péczka and Ivan Ranđelović contributed equally to this work.

Contents

Synthesis procedures 2

$^{1}H/^{13}C$ NMR Spectra and HPLC Traces of Compounds............................ 6

LC-MS/MS peptide mapping data 26

Protein NMR spectra 27

k_{inact} and K_I determination 33

Computational data... 36

References.. 39

Synthesis procedures

7-bromo-6-chloroquinazolin-4(3*H*)-one (**7**)

2-amino-4-bromo-5-chlorobenzoic acid (**5**) (5 g, 19.96 mmol) was dissolved in ethanol (110 ml), then formamidine acetate (**6**) (20.78 g, 199.60 mmol) was added followed by 16 h stirring at 78 °C. The solvent was evaporated, the crude product was rinsed with water, filtered, and dried, yielding the product as a light brown solid (4.56 g, 92 %).

^{1}H-NMR (300 MHz, DMSO-d_6) δ (ppm) 12.50 (s, 1H), 8.14 (d, *J* = 2.5 Hz, 2H), 8.07 (s, 1H)

7-bromo-6-chloroquinazoline-2,4(1*H*,3*H*)-dione (**9**)

2-amino-4-bromo-5-chlorobenzoic acid (**5**) (10.0 g, 40.0 mmol), urea (**8**) (16.8 g, 280.3 mmol) were mixed together and heated to 200 °C for three hours. After cooling to 100 °C, water was added and stirred for 1 hour. The reaction mixture was cooled to room temperature, filtered and washed whith water, then dried in vacuum oven to afford compound **9** as a brownish solid (10,79 g, 98 %).

^{1}H-NMR (300 MHz, DMSO-d_6) δ (ppm) 11.50 (s, 1H), 11.27 (s, 1H), 7.90 (s, 1H), 7.46 (s, 1H)

7-bromo-4,6-dichloroquinazoline (**10a**)

In a heated flask 7-bromo-6-chloroquinazolin-4(3*H*)-one (**7**) (5.05 g 19.46 mmol), $POCl_3$ (45 ml) and DIPEA (3.4 ml) were measured followed by 16h stirring at 106 °C. $POCl_3$ was distilled, then toluene (20 ml) was added and evaporated two times. The crude product was purified with flash chromatography (eluent: hexane:EtOAc), yielding **10a** as a white solid (4.43 g, 82 %).

^{1}H-NMR (500 MHz, DMSO-d_6) δ (ppm) 8.63 (s, 1H), 8.52 (s, 1H), 8.10 (s, 1H)

7-bromo-2,4,6-trichloroquinazoline (**10c**)

Compound **9** (11.0 g, 40.0 mmol) was dissolved in $POCl_3$ (80ml), DIPEA was added (132.0 mmol, 18.9 ml), and the reaction mixture was stirred at 110 °C for overnight. The volatiles were evaporated and azeotroped with toluene to get the residue as a dark brown solid. (11.1g, 89%)

^{1}H-NMR (300 MHz, DMSO-d_6) δ (ppm) 8.52 (s, 1H), 8.44 (s, 1H).

tert-butyl 4-(7-bromo-6-chloroquinazolin-4-yl)piperazine-1-carboxylate (**12a**)

7-bromo-4,6-dichloroquinazoline (**10a**) (4.43g, 15.96mmol) was dissolved in DCM (80 ml) and TEA (6.7 ml, 47.88 mmol). *tert*-butyl piperazine-1-carboxylate (**11**) (5.93 g, 31.29 mmol) in DCM (20 ml) was added dropwise. The reaction mixture was stirred at r.t. for 40 minutes, then 1 M HCl was added. The organic phase was washed with saturated $NaHCO_3$ solution, and brine, then dried over Na_2SO_4. The solvent was evaporated. The crude product was washed with petrolether/EtOAc 5:1, filtered and dried, yielding the product as a light yellow solid (6.82 g, 82 %).

^{1}H NMR (500 MHz, $CDCl_3$) δ (ppm) 8.70 (s, 1H), 8.27 (s, 1H), 7.95 (s, 1H), 3.81-3.77 (m, 4H), 3.67-3.63 (m, 4H), 1.49 (s, 9H)

tert-butyl 4-(7-bromo-6-chloro-2-hydroxyquinazolin-4-yl)piperazine-1-carboxylate (**12c**)

To a solution of **10c** (12.5 g, 40.0 mmol) in DCM (100 ml) *tert*-butyl piperazine-1-carboxylate (**11**) (14.5 g, 120 mmol) and TEA (16.8 ml, 120 mmol) was added at 0 °C, then the reaction mixture was stirred at room temperature for 40 min. The mixture was washed twice with 1 M HCl solution, once with distilled water, twice with saturated $NaHCO_3$ solution, and once with saturated NaCl solution. The organic layer was dried over Na_2SO_4 and concentrated under vacuum. The crude product was washed with mixture of petroleum ether/ethyl acetate 5:1 to afford the product as brown solid (13.5g, 73 %)

tert-butyl 4-(6-chloro-7-(2-fluoro-6-hydroxyphenyl)quinazolin-4-yl)piperazine-1-carboxylate (**14a**)

12a (1.59 g, 3.73 mmol), boronic acid (**13a**) (3.17 g, 18.66 mmol), Na_2CO_3 (1.19 g, 11.20 mmol) and $Pd(PPh_3)_2Cl_2$ (0.43 g, 0.373 mmol) were dissolved in dioxane-water 4:1 (90 ml). The mixture was stirred at 90 °C for 16 h under argon. After the reaction the solvents were evaporated. The crude product was purified by flash column chromatography (hexane/EtOAc), giving the product as yellowish crystals (1.19 g, 70 %).

^{1}H NMR (500 MHz, DMSO-d_6) δ (ppm) 10.08 (s, 1H) 8.66 (s, 1H), 8.13, (s, 1H), 7.75 (s, 1H), 7.30 (dd, *J*=15.3, 8.2 Hz, 1H), 6.83 (d, *J*=8.3 Hz, 1H), 6.77 (t, *J* = 8.7 Hz, 1H), 3.84 – 3.75 (m, 4H), 3.57 (s, 4H), 1.44 (s, 9H).

tert-butyl 4-(6-chloro-7-(2-fluoro-6-hydroxyphenyl)-2-((1-methylpyrrolidin-2-yl)methoxy)quinazolin-4-yl)piperazine-1-carboxylate (**14b**)

To a solution of *N*-methyl-L-prolinol (4.06 mmol, 0.48 ml) in THF (50 ml) NaH (60 %, 162 mg, 4.057 mmol) was added, and after stirring for 30 minutes, **14c** (1g, 2.028 mmol) was added. The resulting mixture was stirred for 2 hours, then diluted with water, and extracted with EtOAc. The combined organic layers were dried over Na_2SO_4 and concentrated under vacuum, to afford the product as a yellow solid (1.07g, 93 %).

^{1}H NMR (500 MHz, $CDCl_3$) δ (ppm) 8.06 (s, 1H), 7.57 (s, 1H), 7.26 (td, J = 8.3, 6.6 Hz, 1H), 6.76 (d, J = 8.3 Hz, 1H), 6.69 (t, J = 8.7 Hz, 1H), 4.51 – 4.40 (m, 2H), 3.93 – 3.87 (m, 4H), 3.72 – 3.65 (m, 4H), 3.13 – 3.08 (m, 1H), 2.83 – 2.77 (m, 1H), 2.53 (s, 3H), 2.38 (q, J = 8.9 Hz, 1H), 2.17 – 2.08 (m, 1H), 1.88 – 1.80 (m, 2H), 1.79 – 1.72 (m, 1H), 1.51 (s, 9H).^{13}C NMR (500 MHz, CD_3OD) δ (ppm) 165.7, 161.6, 159.4, 156.1, 155.0, 151.8, 137.5, 129.9, 129.9, 129.6, 129.0, 124.9, 113.3, 110.9, 110.9, 105.7, 105.5, 80.2, 69.0, 64.3, 57.2, 49.0, 40.7, 28.1, 27.3, 22.1.

tert-butyl 4-(6-chloro-7-(2-fluoro-6-hydroxyphenyl)-2-hydroxyquinazolin-4-yl)piperazine-1-carboxylate (**14c**)

To a solution of **12c** (1.0 g, 2.16 mmol) in dioxane:water 4:1 (80 ml) (2-fluoro-6-hydroxyphenyl)boronic acid (**13a**) (0.37 g, 2.38 mmol), Na_2CO_3 (0.69 g, 6.48 mmol) and $Pd(PPh_3)_4$ (0.25 g, 0.22 mmol) was added, and the reaction mixture was stirred at 90 °C overnight. After cooling to room temperature, the solvents were removed under vacuum, then EtOAc was added, and the suspension was filtered through celite pad, the filtratum was concentrated under vacuum. The crude product was purified via flash column chromatography (eluent: hexane:EtOAc 4:1), affording product as a yellow solid (0.19g, 18 %)

^{1}H NMR (500 MHz, $CDCl_3$) δ (ppm) 7.96 (s, 1H), 7.90 (s, 1H), 7.31 – 7.22 (m, 1H), 7.00 (d, J = 8.3 Hz, 1H), 6.72 (t, J = 8.5 Hz, 1H), 4.00 – 3.85 (m, 4H), 3.73 – 3.61 (m, 4H), 1.51 (s, 9H).

^{13}C NMR (500 MHz, $CDCl_3$) δ (ppm) 163.8, 156.4, 155.4, 155.4, 154.7, 150.7, 137.9, 132.6, 131.1, 130.7, 125.1, 114.4, 113.3, 112.2, 106.8, 80.7, 60.4, 49.2, 28.4, 14.2.

tert-butyl 4-(6-chloro-7-(2-fluoro-6-methoxyphenyl)quinazolin-4-yl)piperazine-1-carboxylate (**14d**)

12a (1.0 g, 2.16 mmol), boronic acid (**13d**) (0.405 g, 2.38 mmol), Na_2CO_3 (0.689 g, 6.50 mmol) and $Pd(PPh_3)_2Cl_2$ (0.15 g, 0.22 mmol) were dissolved in dioxane-water 4:1 (80 ml). The mixture was stirred at 90 °C for 16 h under argon. After the reaction the solvents were evaporated. The crude product was purified by flash column chromatography (hexane/EtOAc), giving the product as yellowish crystals (0.62 g, 61 %).

^1H NMR (500 MHz, dmso) δ 8.61 (s, 1H), 8.09 (s, 1H), 7.72 (s, 1H), 7.25 (q, *J* = 8.1 Hz, 1H), 6.81 (d, *J* = 8.3 Hz, 1H), 6.72 (t, *J* = 8.8 Hz, 1H), 3.79 – 3.74 (m, 4H), 3.58 – 3.50 (m, 4H), 2.46 (s, 3H), 1.39 (s, 9H).

2-(6-chloro-4-(piperazin-1-yl)quinazolin-7-yl)-3-fluorophenol (**15a**)

14a (276 mg, 0.602 mmol) was dissolved in DCM (6.3 ml), then 2,2,2-trifluoroacetic acid was added (790 µl). The mixture was stirred for 40 min at r.t. then the solvent was evaporated. $NaHCO_3$ was added and washed with EtOAc. The organic phase was washed with brine and dried on Na_2SO_4. The solvent was evaporated resulting in 209.9 mg (97 %) yellow powder.

^1H NMR (500 MHz, CD_3OD) δ (ppm) 8.67 (s, 1H), 8.17 (s, 1H), 8.15 (s, 1H), 7.79 (s, 1H), 7.28 (td, *J* = 8.3, 6.6 Hz, 1H), 6.77 (d, *J* = 8.2 Hz, 1H), 6.71 (t, *J* = 8.7 Hz, 1H), 3.97-3.88 (m, 4H), 3.79-3.68 (m, 4H).

Exact Mass: Calc: 357.0923, Found: 357.0942

2-(6-chloro-2-((1-methylpyrrolidin-2-yl)methoxy)-4-(piperazin-1-yl)quinazolin-7-yl)-3-fluorophenol (**15b**)

To a solution of **14b** (1.18 g, 2.068 mmol) in DCM (100 ml) TFA (7.7 ml) was added, then stirred for 2 hours at room temperature. Then the volatiles were evaporated under vacuum. Saturated $NaHCO_3$ solution was added, then extracted with EtOAc. The combined organic layers were dried over Na_2SO_4 and concentrated under vacuum, to afford the product as a yellow solid (0.91g, 93%).

^1H NMR (500 MHz, $CDCl_3$) δ (ppm) 7.46 (s, 1H), 7.04 (s, 1H), 6.71 – 6.62 (m, 1H), 6.18 (d, J = 8.3 Hz, 1H), 6.05 (t, J = 8.7 Hz, 1H), 3.97 – 3.87 (m, 1H), 3.89 – 3.81 (m, 1H), 2.81 – 2.76 (m, 1H), 2.58 – 2.46 (m, 5H), 2.27 – 2.17 (m, 1H), 1.97 (s, 3H), 1.80 (q, J = 9.0 Hz, 1H), 1.62 – 1.51 (m, 1H), 1.34 – 1.14 (m, 3H).

^{13}C NMR (500MHz, CD3OD) δ (ppm) 169.0, 165.8, 161.6, 151.8, 138.1, 129.7, 129.6, 129.2, 124.8, 114.3, 114.2, 113.2, 111.8, 104.3, 104.2, 69.1, 69.1, 64.2, 57.2, 50.2, 45.0, 40.7, 28.1, 22.1.

Exact Mass: Calc: 472.191, Found: 472.1927

2-(6-chloro-2-((1-methylpyrrolidin-2-yl)methoxy)-4-(piperazin-1-yl)quinazolin-7-yl)-3-fluorophenol (**15d**)

To a solution of **14d** (1.00 g, 2.11 mmol) in DCM (100 ml) TFA (7.7 ml) was added, then stirred for 2 hours at room temperature. Then the volatiles were evaporated under vacuum. Saturated $NaHCO_3$ solution was added, then extracted with EtOAc. The combined organic layers were dried over Na_2SO_4 and concentrated under vacuum, to afford the product as a yellow solid (0.73g, 92%).

^1H NMR (500 MHz, CD_3OD) δ 8.65 (s, 1H), 8.19 (s, 1H), 7.78 (s, 1H), 7.31 – 7.25 (m, 1H), 6.77 (d, *J* = 8.3 Hz, 1H), 6.71 (t, *J* = 8.6 Hz, 1H), 4.34 (s, 3H), 3.99 (dd, *J* = 23.3, 5.6 Hz, 4H), 3.84 (dd, *J* = 6.6, 3.6 Hz, 4H).

(4-(6-chloro-7-(2-fluoro-6-hydroxyphenyl)quinazolin-4-yl)piperazin-1-yl)(1-tritylaziridin-2-yl)methanone (**19a**)

15a (95 mg, 0.26 mmol), PyAOP (152 mg, 0.29 mmol), HOBt.H_2O (39 mg, 0.29 mmol) were dissolved in acetonitrile (15 ml). DIPEA (184 µl, 1 mmol) was added and the mixture was cooled to 0 °C, and 1-(triphenylmethyl)aziridine-2-carboxilate (**18**) (104 mg, 0.29 mmol) was added, followed by 16 h stirring at r.t. After the reaction water was added and stirred for 30 min then extracted with DCM. The organic phase was dried on Na_2SO_4 and then was evaporated. The product was used directly in the following reaction.

(4-(6-chloro-7-(2-fluoro-6-hydroxyphenyl)-2-((1-methylpyrrolidin-2-yl)methoxy)quinazolin-4-yl)piperazin-1-yl)(1-tritylaziridin-2-yl)methanone (**19b**)

The solution of **15b** (71 mg, 0.150 mmol) in MeCN (3 ml) was cooled to 0 °C, then 1-tritylaziridine-2-carboxylic acid (**18**) (54 mg, 0.165 mmol), HOBt.H_2O (22 mg, 0.165 mmol), PyAOP (94 mg, 0.180 mmol) and DIPEA (0.1 ml, 0.601 mmol) were added. The reaction mixture was stirred overnight at room temperature. Distilled water was added, then extracted with EtOAc. The combined organic layers were dried over Na_2SO_4 and concentrated under vacuum, affording the product as a yellow oil. The product was used directly in the following reaction.

$^{1}H/^{13}C$ NMR Spectra and HPLC Traces of Compounds

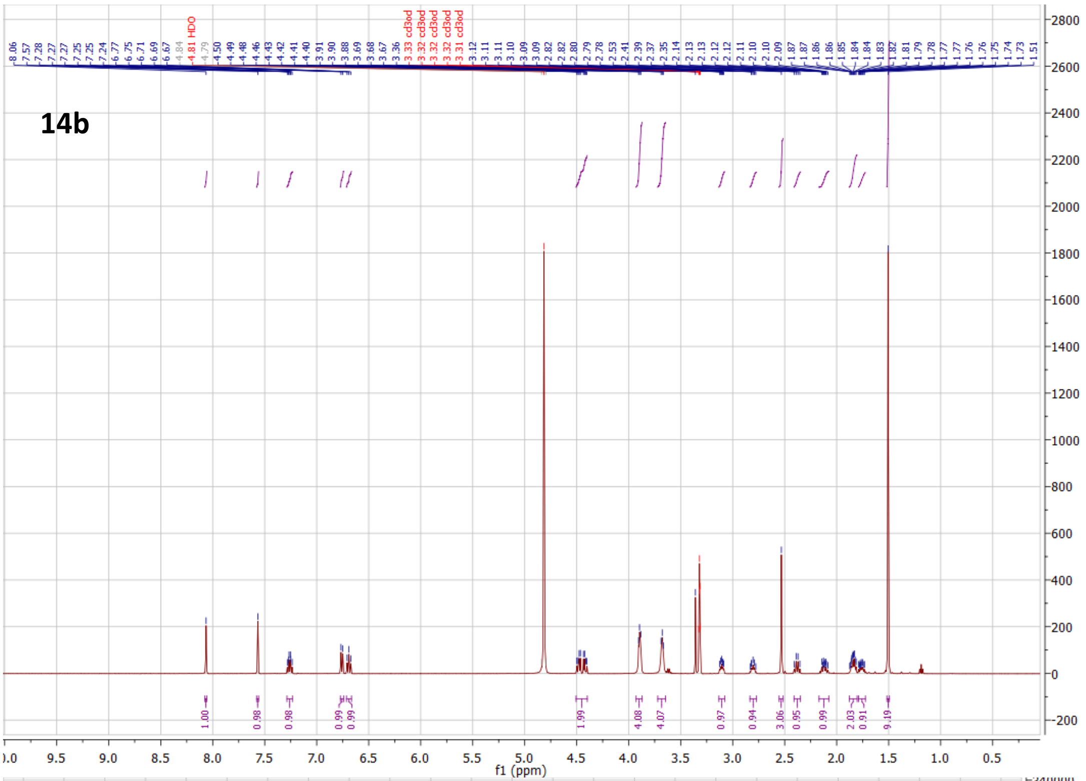

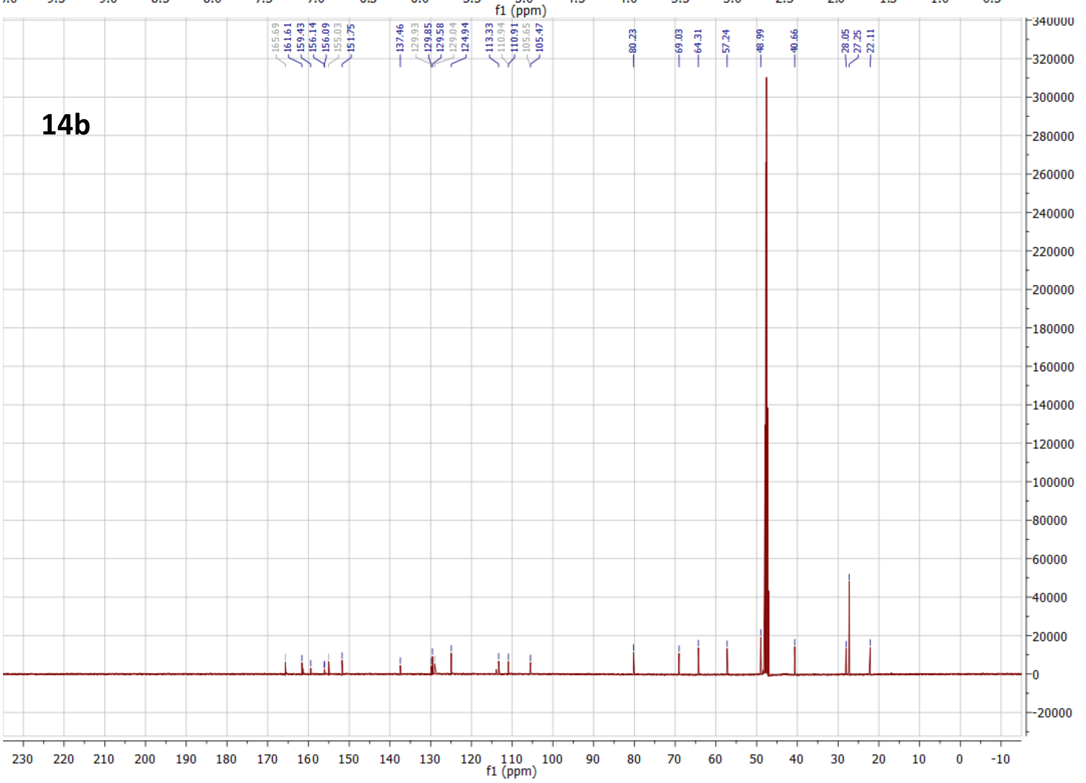

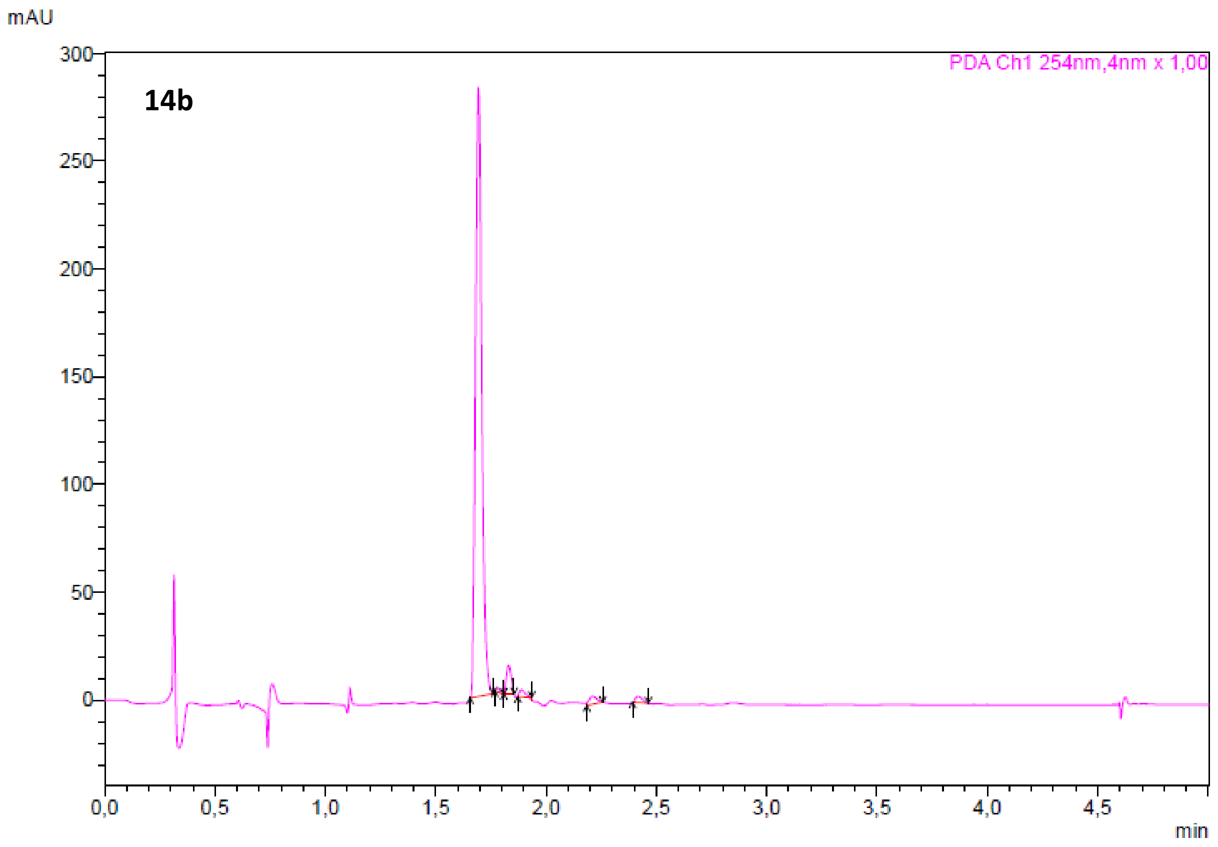

Peak#	Ret. Time	Height	Area	Area%
1	1.694	276433	557177	92.997
2	1.779	2013	2042	0.341
3	1.831	12603	19555	3.264
4	1.890	3074	5353	0.893
5	2.212	3647	9133	1.524
6	2.418	2768	5874	0.980
Total		300537	599134	100.000

15b

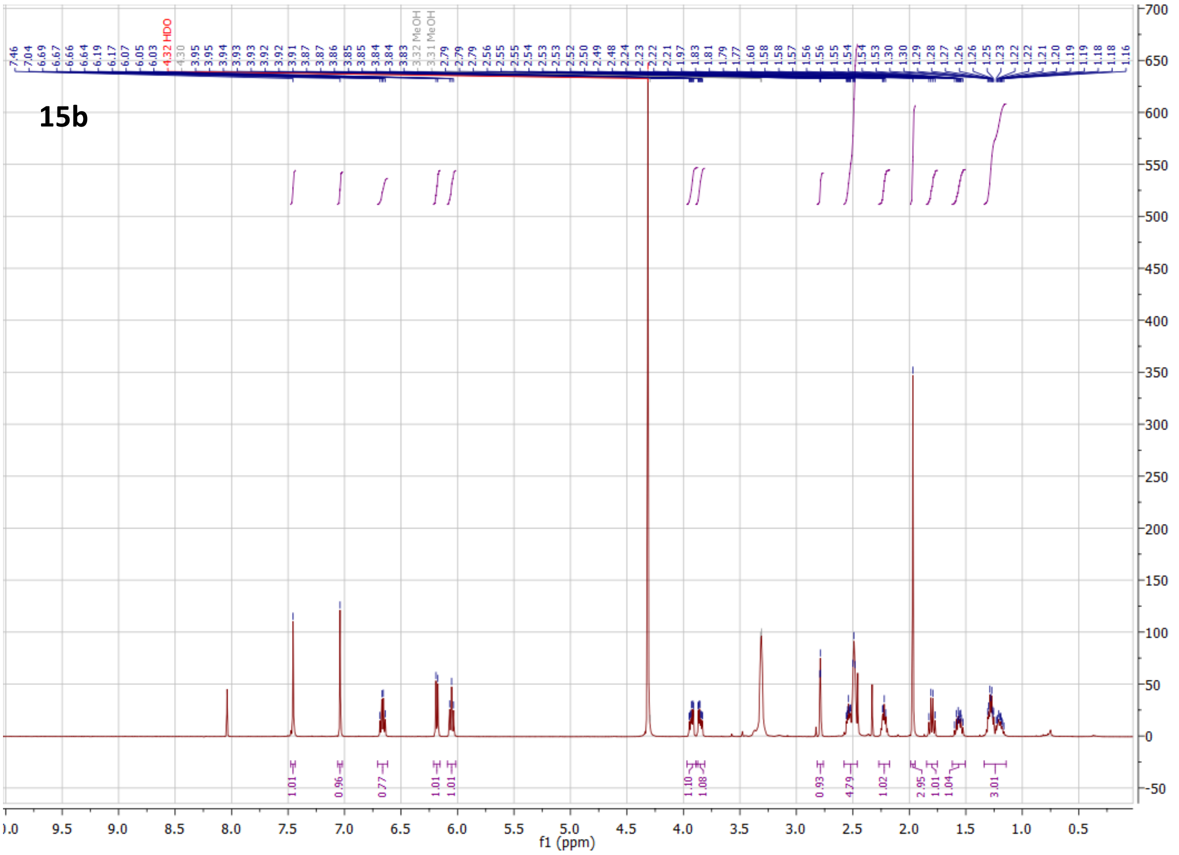

15b

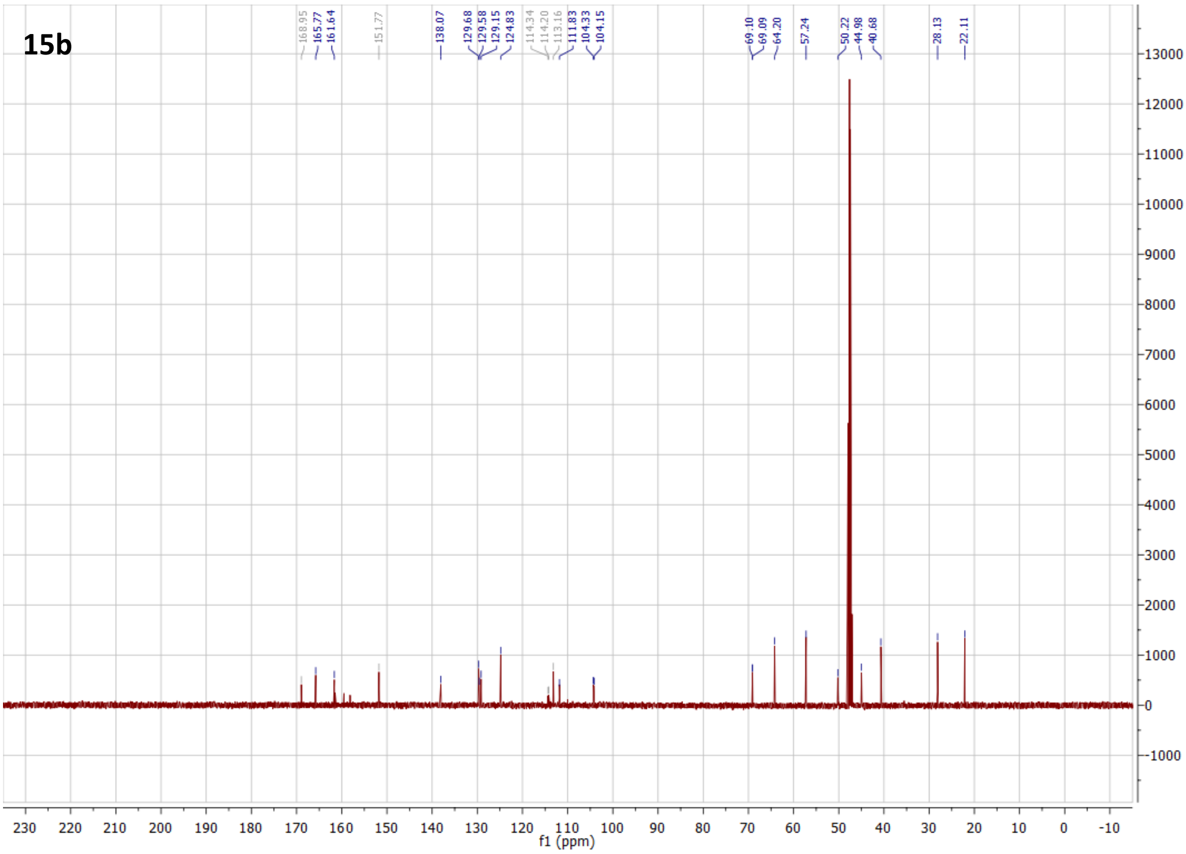

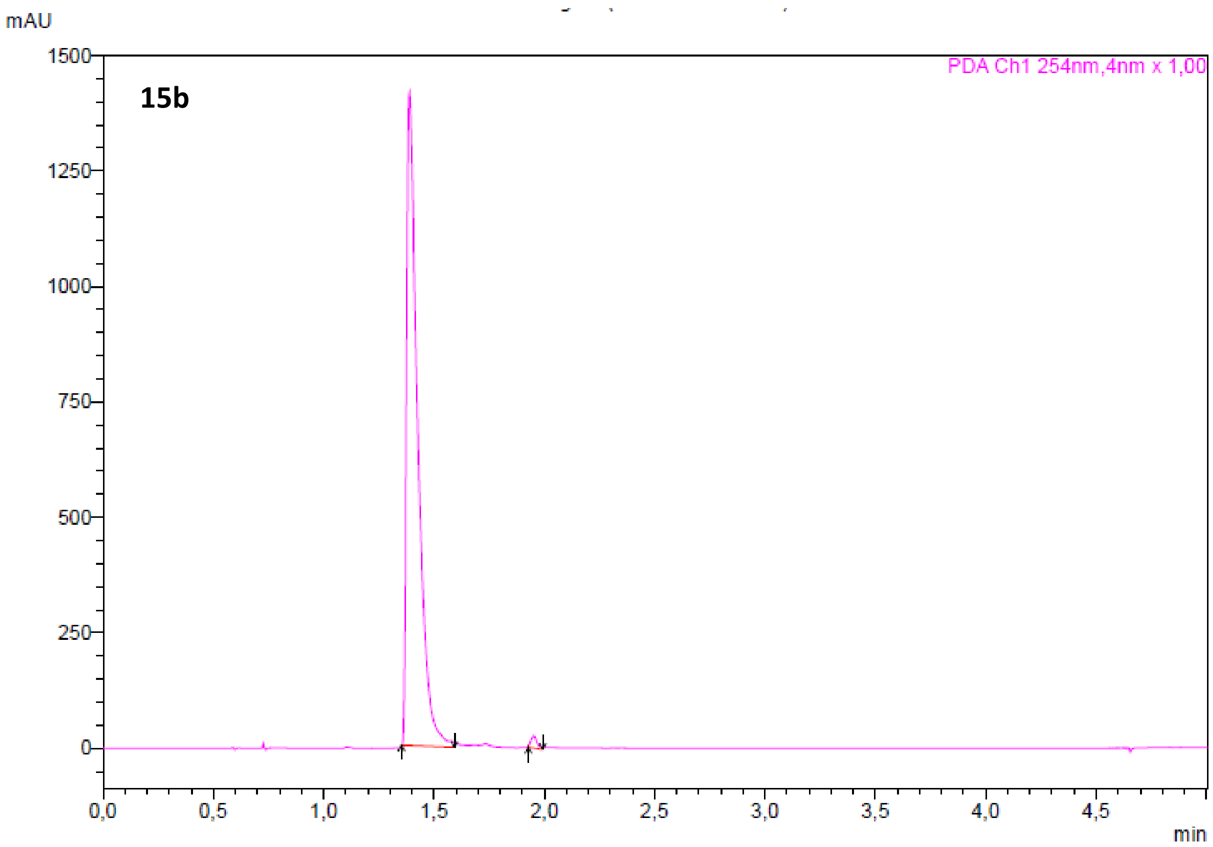

Peak#	Ret. Time	Height	Area	Area%
1	1.390	1402868	4979127	98.988
2	1.951	24701	50891	1.012
Total		1427568	5030018	100.000

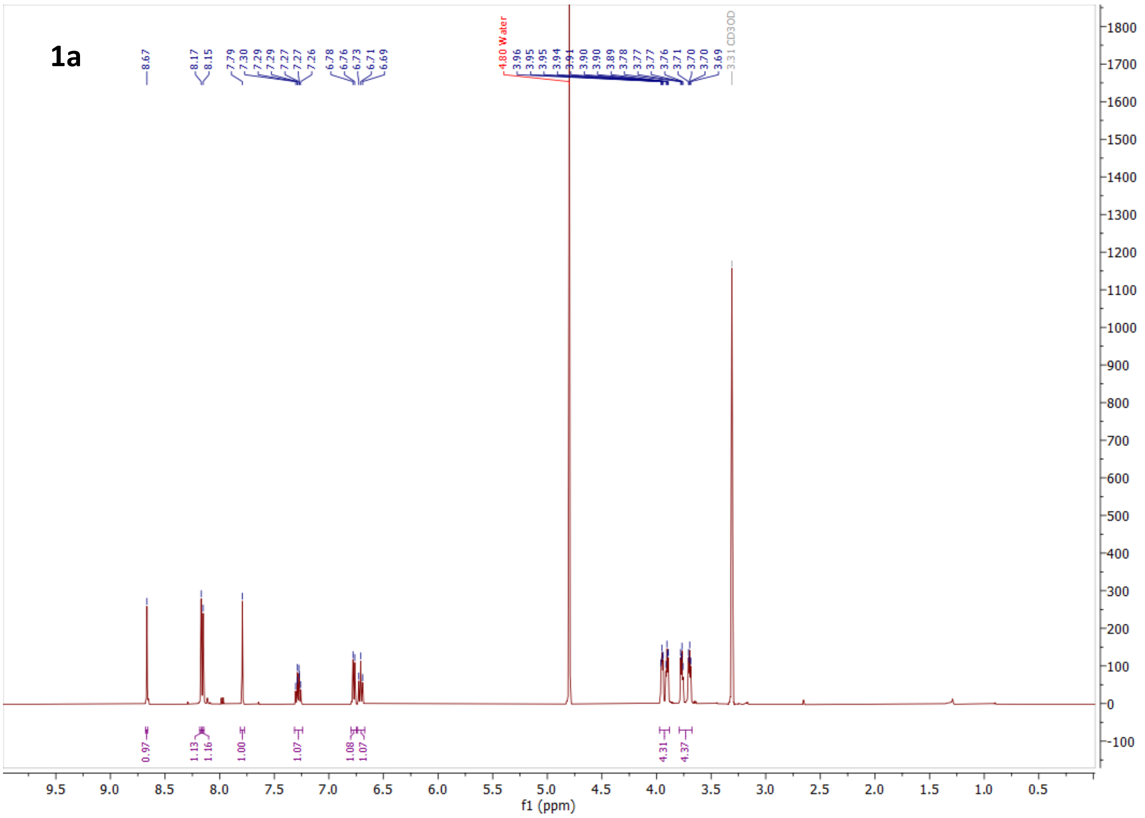
1a
8.67
8.17
8.15
7.79
7.30
7.29
7.29
7.29
7.27
7.27
7.26
6.78
6.76
6.73
6.71
6.69
4.80 Water
3.96
3.95
3.95
3.94
3.91
3.90
3.90
3.89
3.78
3.77
3.77
3.76
3.71
3.70
3.69
3.31 CD3OD
0.97
1.13
1.16
1.00
1.07
1.08
1.07
4.31
4.37
f1 (ppm)

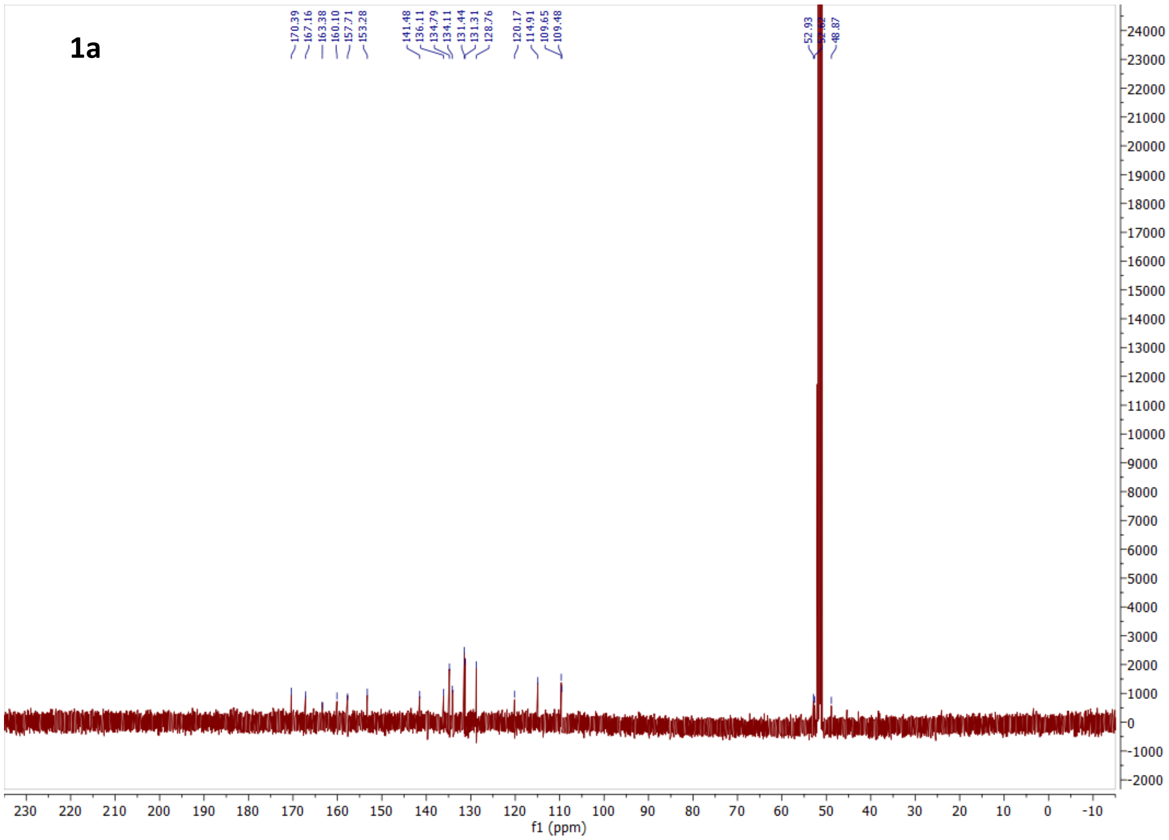
1a
170.39
167.16
163.38
160.10
157.71
153.28
141.48
136.11
134.79
134.11
131.44
131.31
128.76
120.17
114.91
109.65
109.48
52.93
48.87
f1 (ppm)

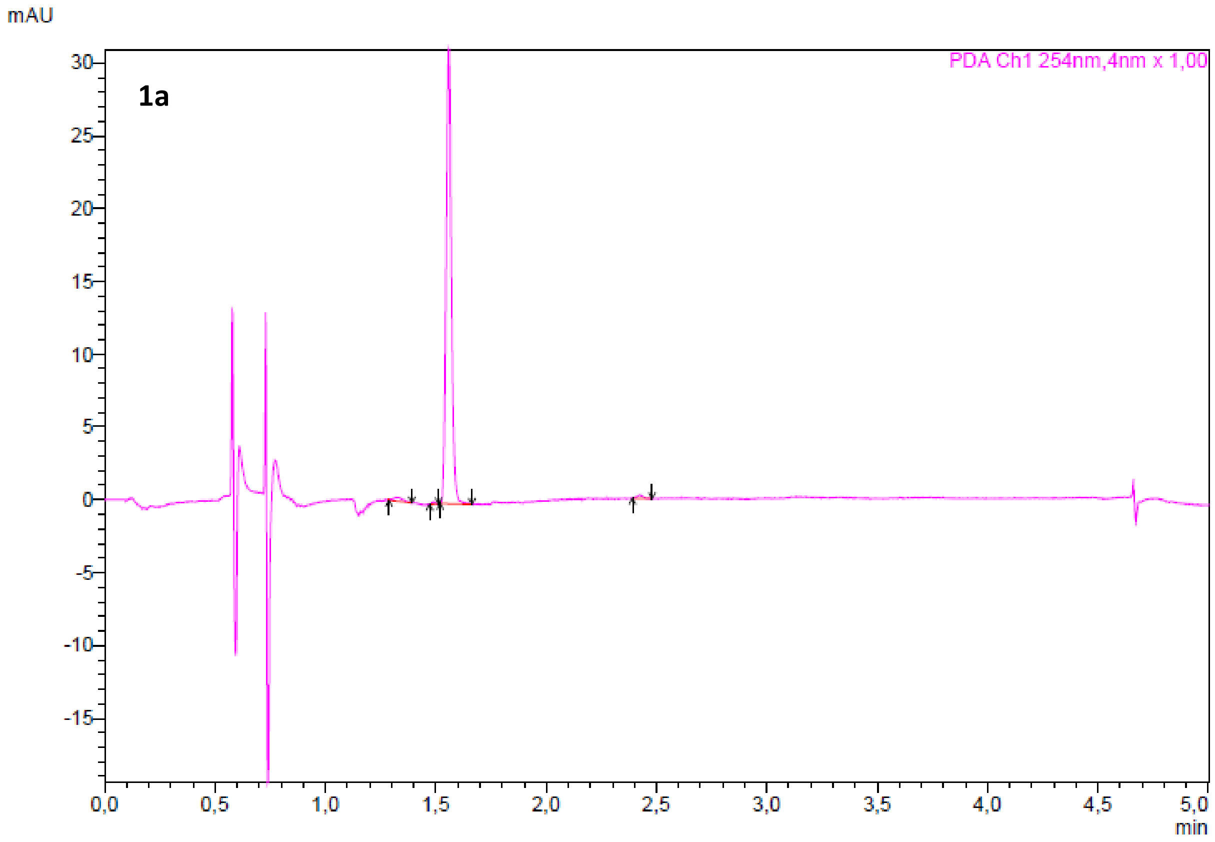

Peak#	Ret. Time	Height	Area	Area%
1	1.320	226	724	1.360
2	1.490	137	157	0.295
3	1.559	30011	51908	97.481
4	2.425	224	460	0.864
Total		30597	53250	100.000

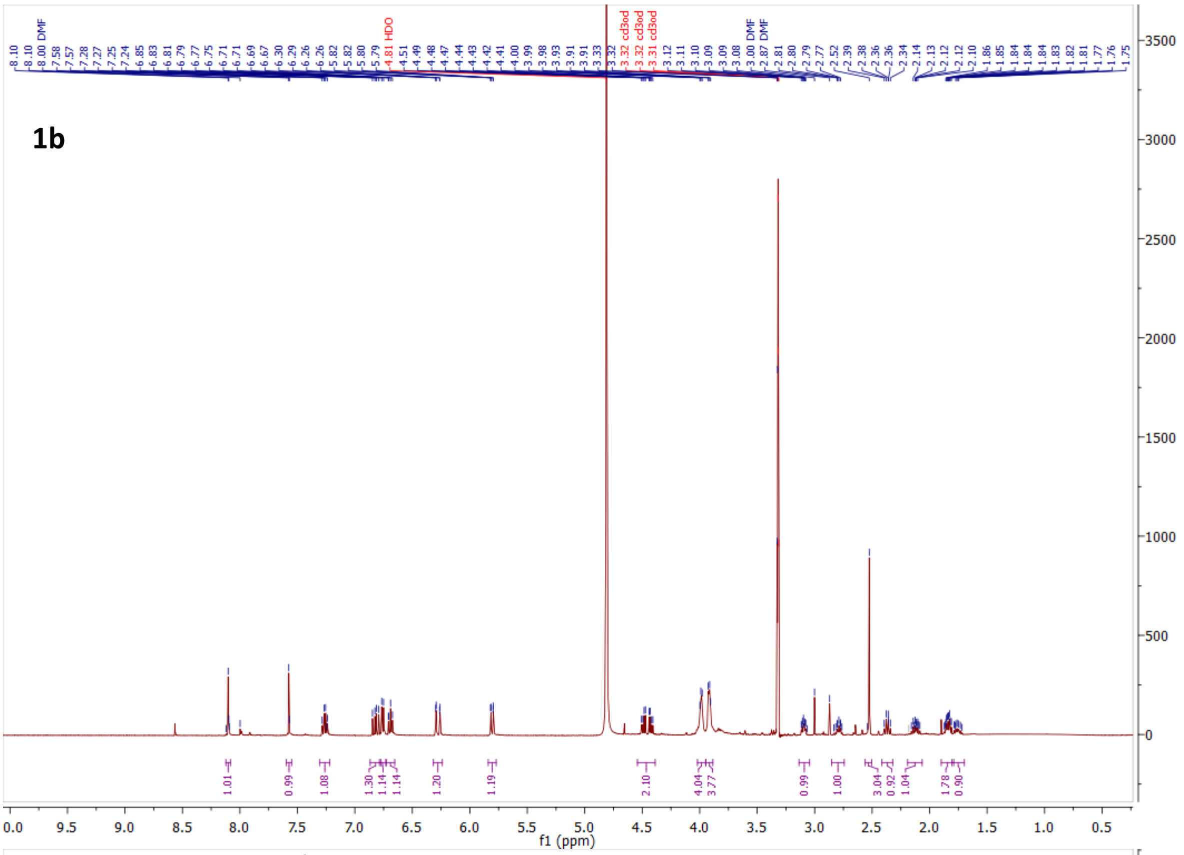
1b
f1 (ppm)

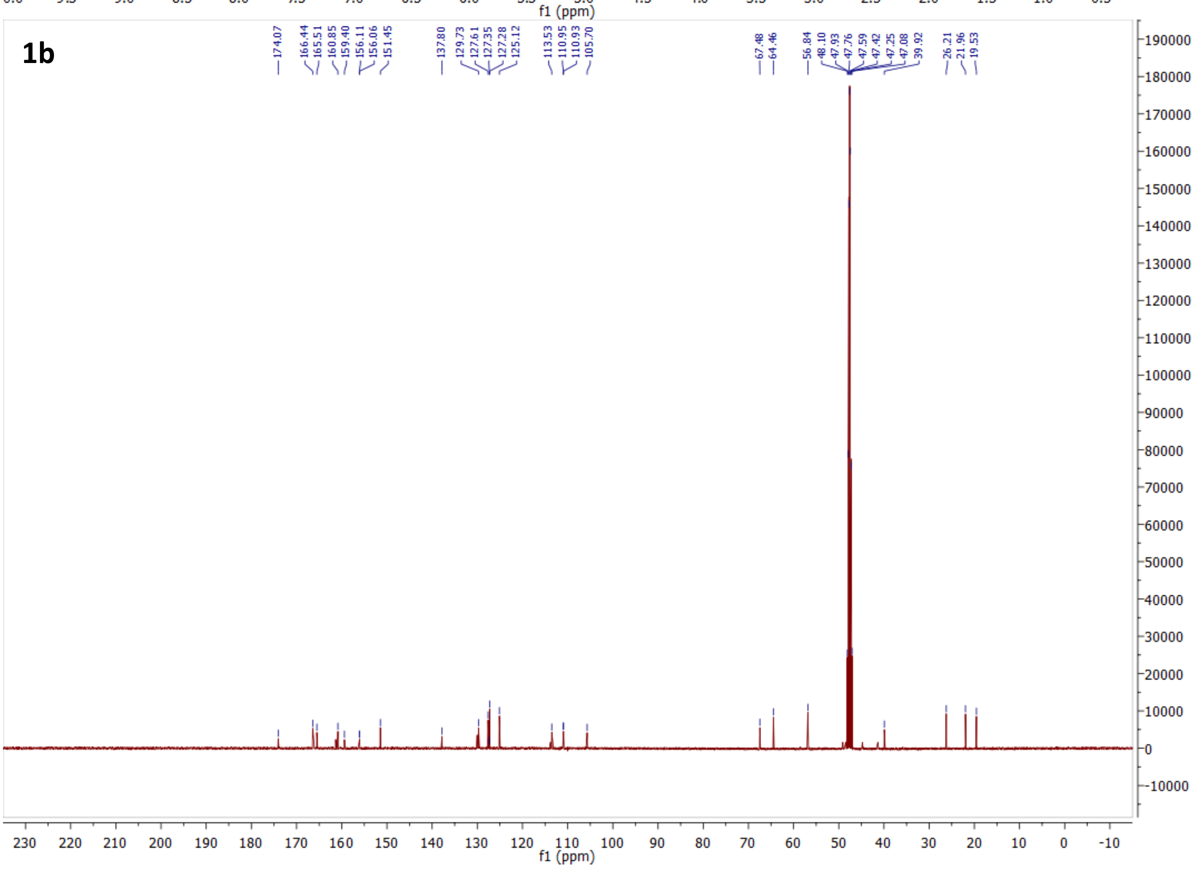
1b
f1 (ppm)

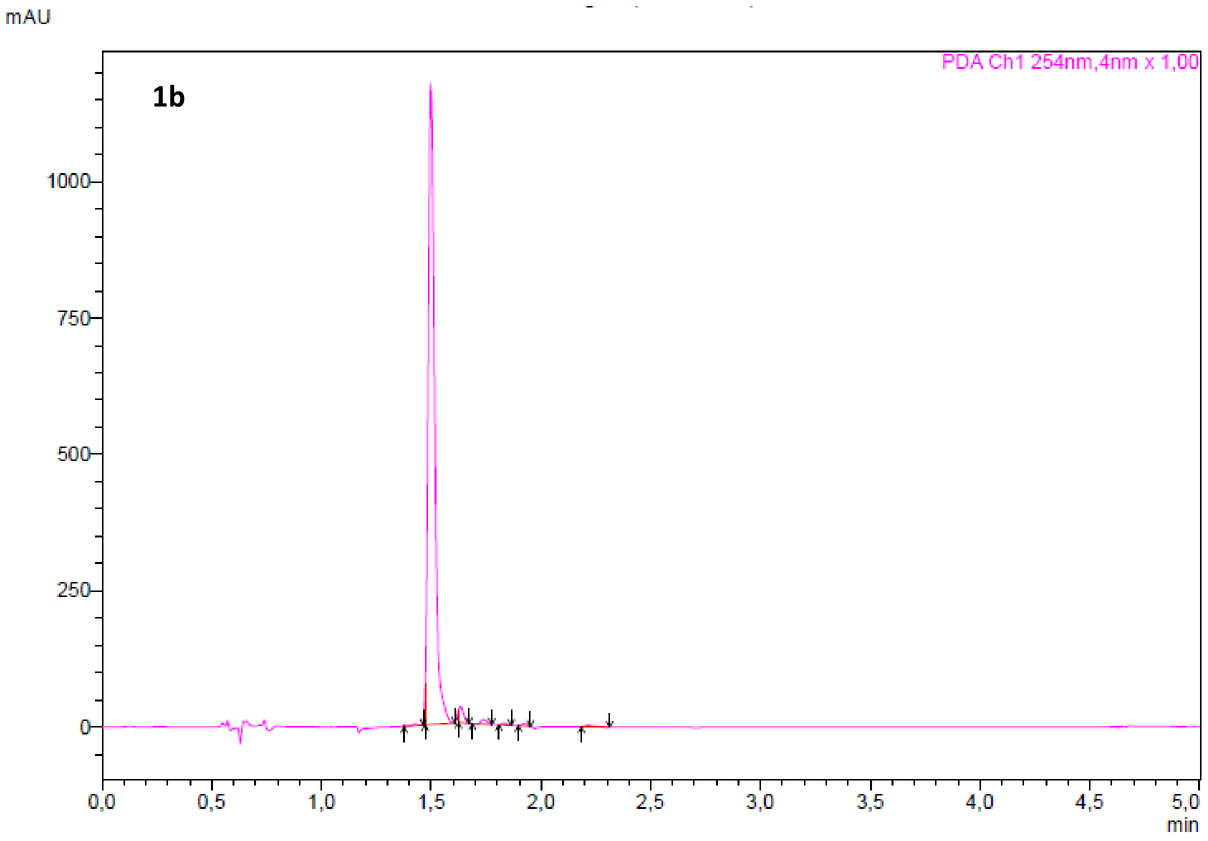

Peak#	Ret. Time	Height	Area	Area%
1	1.428	3043	10020	0.400
2	1.500	1145742	2410321	96.237
3	1.633	27520	43867	1.751
4	1.739	8401	17340	0.692
5	1.832	3276	5854	0.234
6	1.923	4408	8817	0.352
7	2.215	2836	8361	0.334
Total		1195225	2504580	100.000

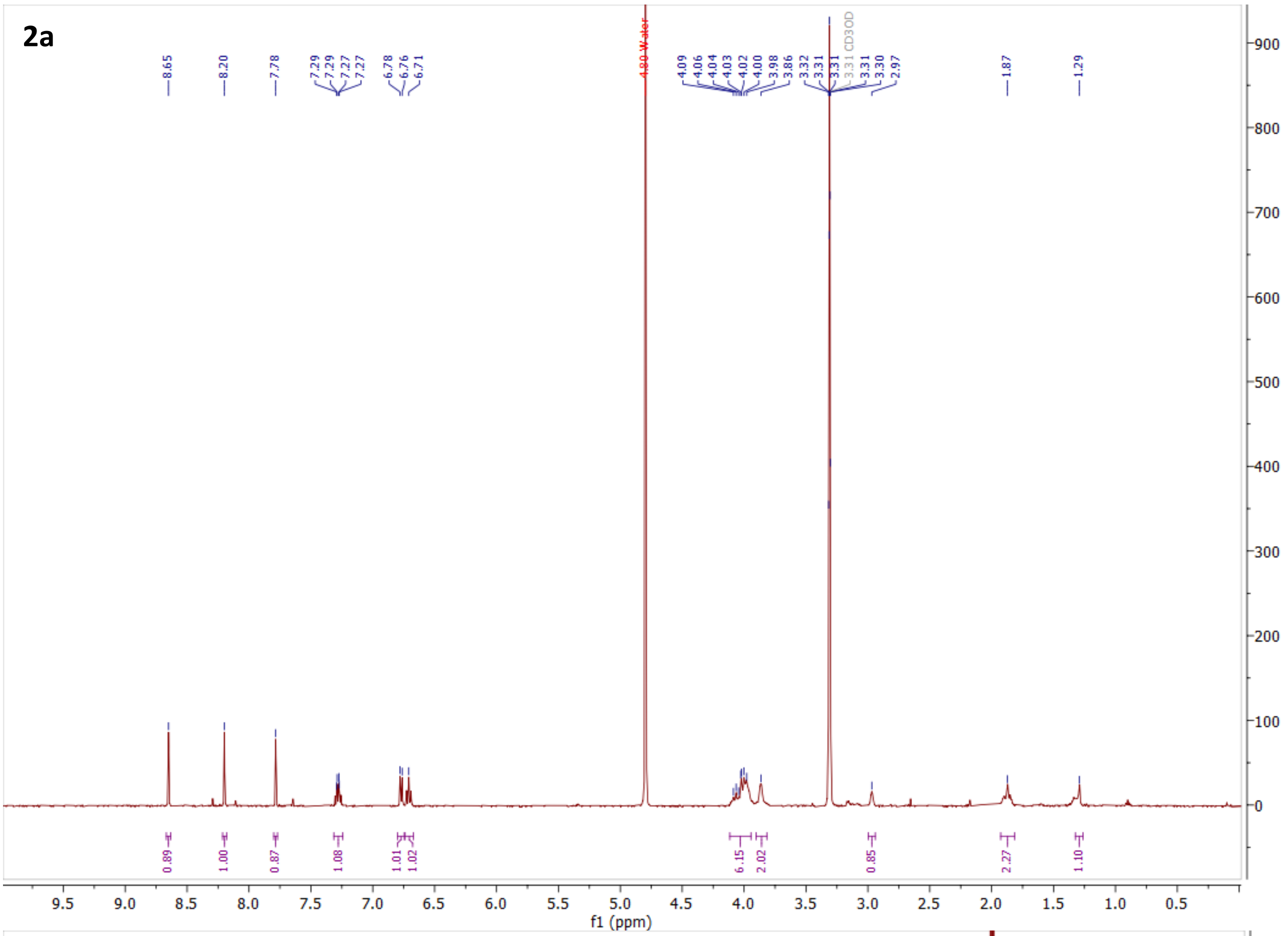
2a
8.65
8.20
7.78
7.29
7.29
7.27
7.27
6.78
6.76
6.71
4.80 Water
4.09
4.06
4.04
4.03
4.02
4.00
3.98
3.86
3.32
3.31
3.31 CD3OD
3.31
3.30
2.97
1.87
1.29
0.89
1.00
0.87
1.08
1.01
1.02
6.15
2.02
0.85
2.27
1.10
f1 (ppm)

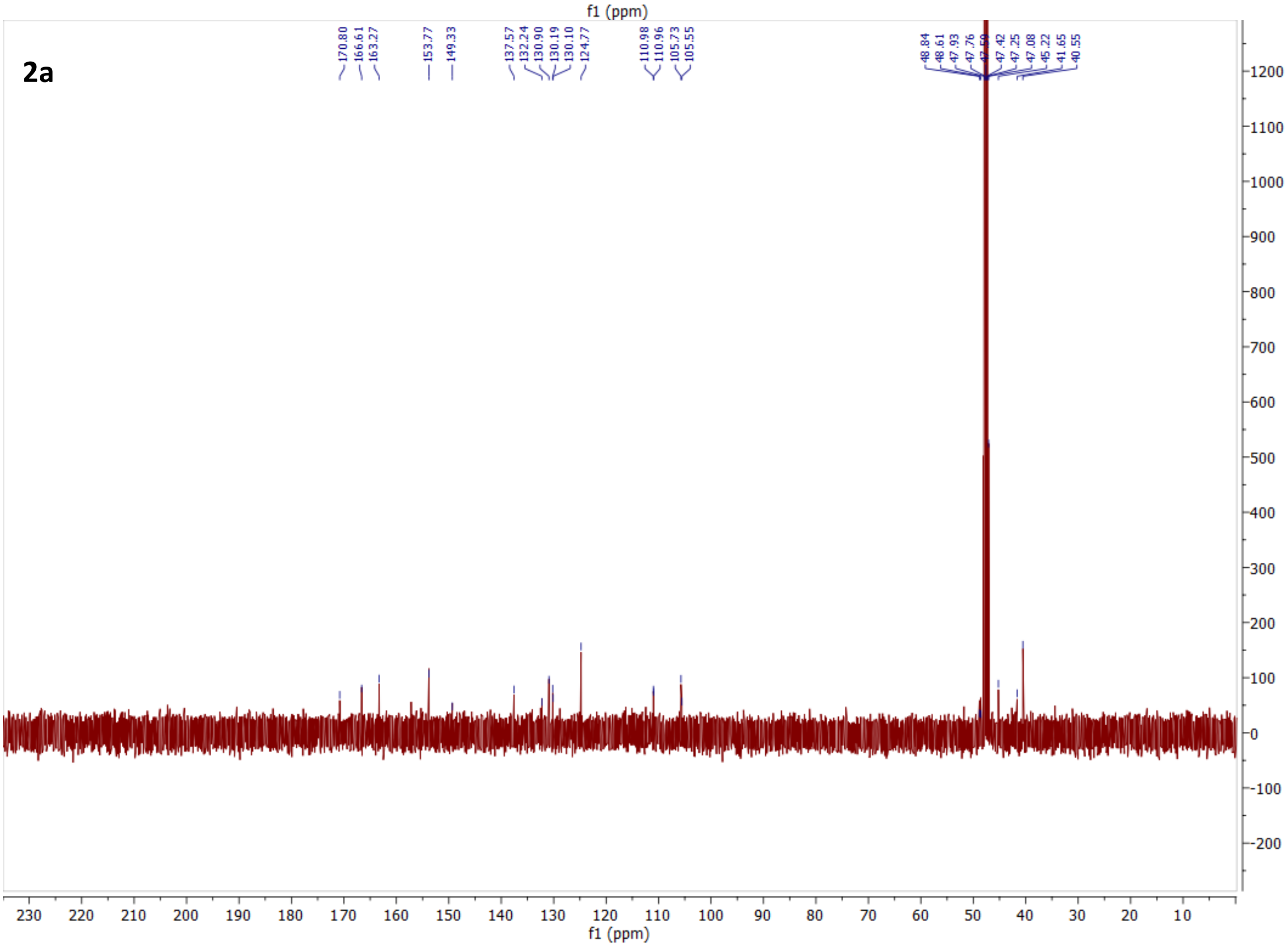
2a
170.80
166.61
163.27
153.77
149.33
137.57
132.24
130.90
130.19
130.10
124.77
110.98
110.96
105.73
105.55
48.84
48.61
47.93
47.76
47.42
47.25
47.08
45.22
41.65
40.55
f1 (ppm)

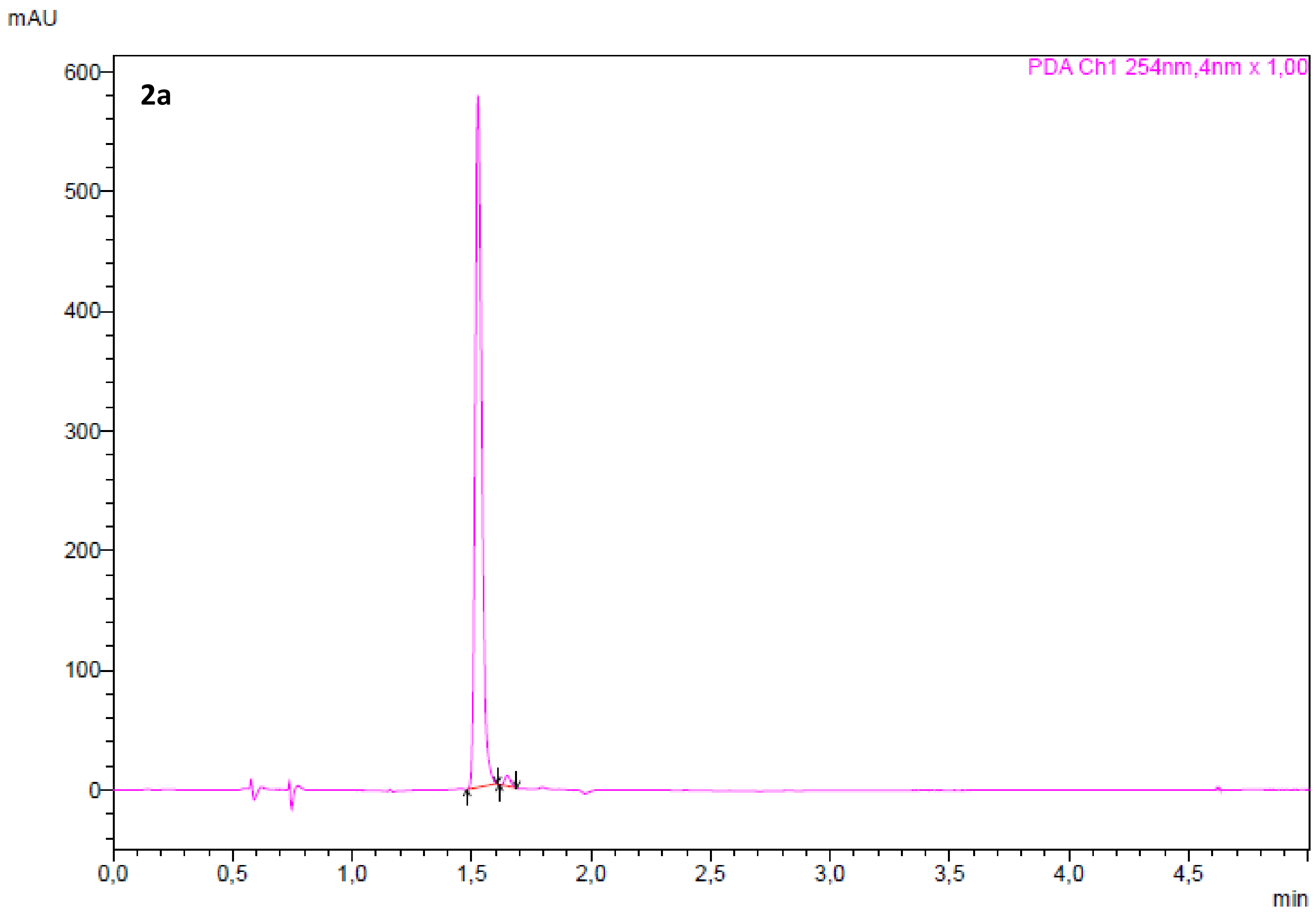

Peak#	Ret. Time	Height	Area	Area%
1	1.527	558520	1109353	98.776
2	1.650	8226	13748	1.224
Total		566746	1123101	100.000

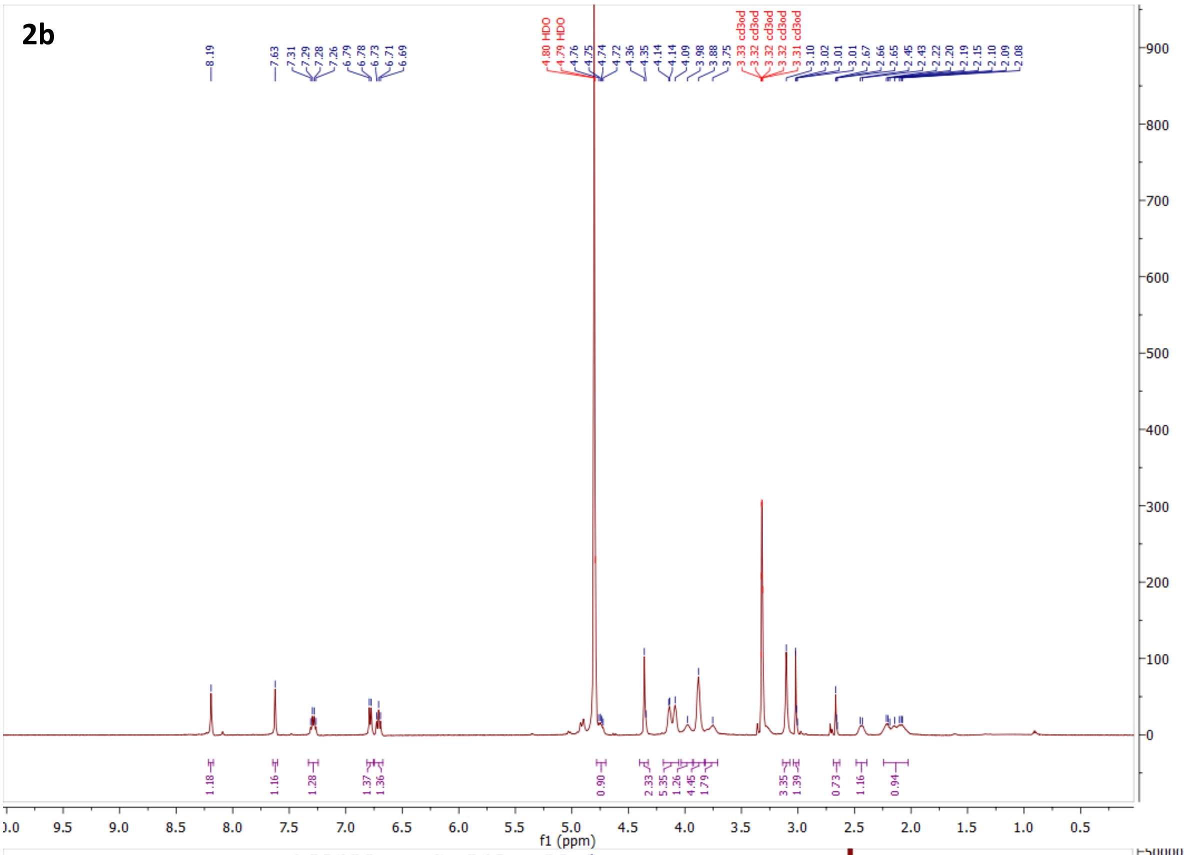
2b
f1 (ppm)

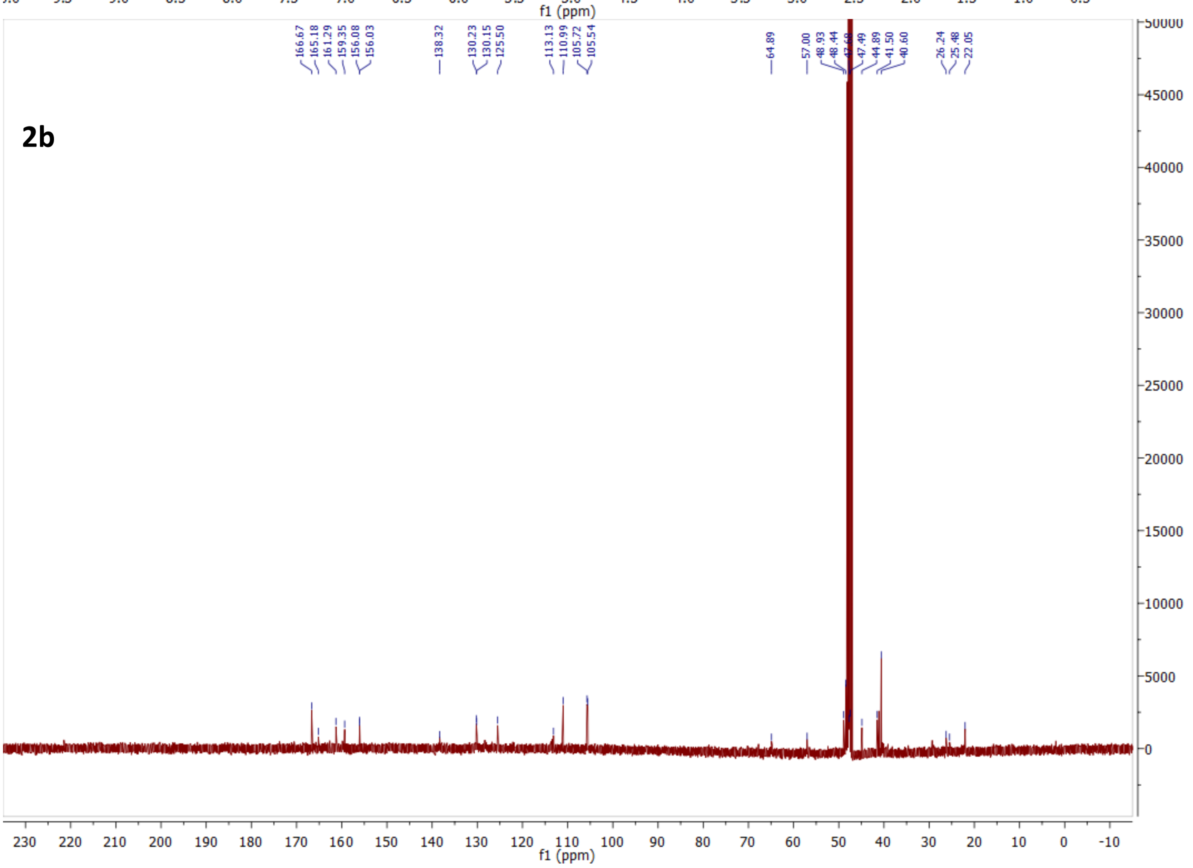
2b
f1 (ppm)

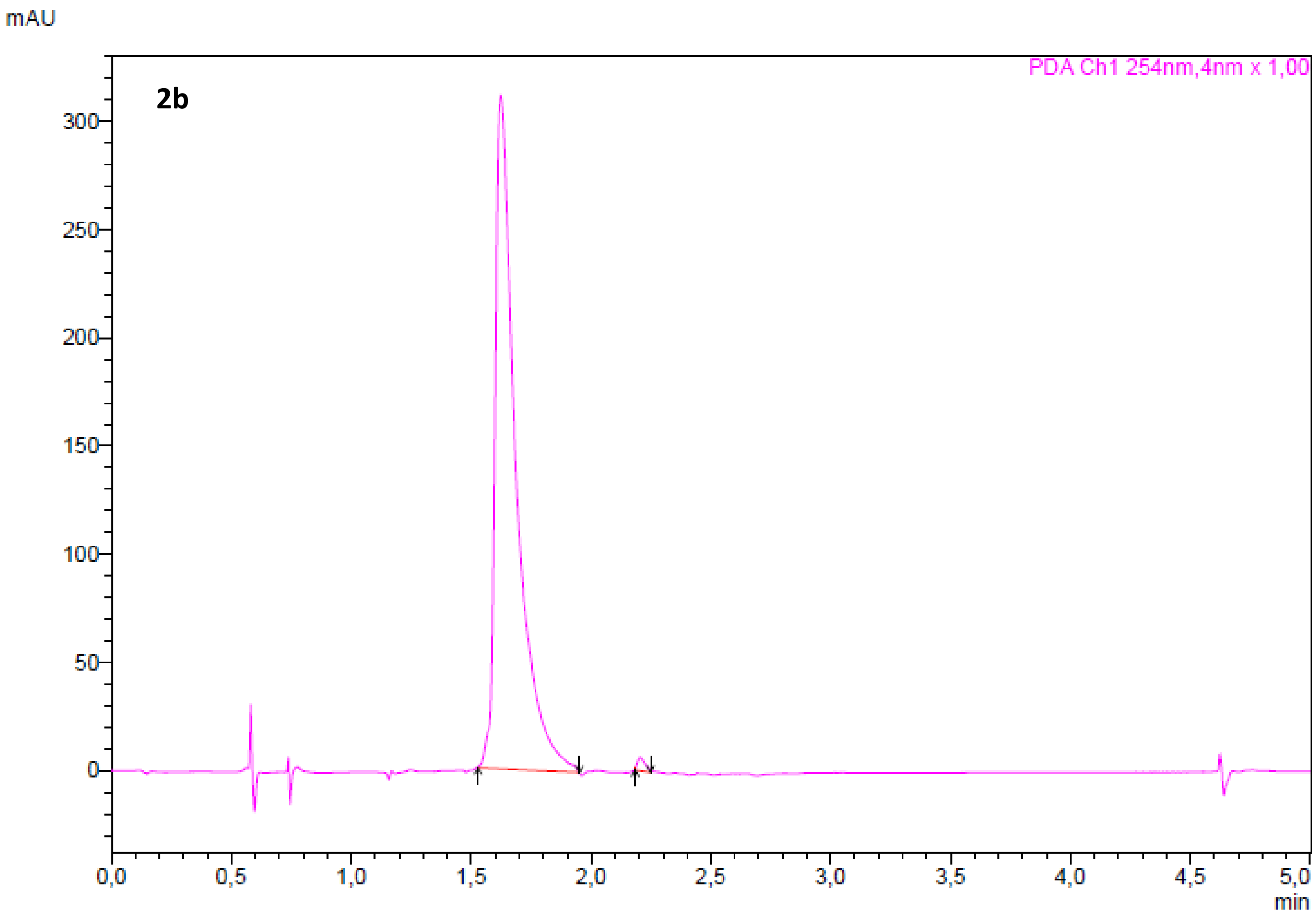

Peak#	Ret. Time	Height	Area	Area%
1	1.624	309125	1897060	99.247
2	2.206	6249	14392	0.753
Total		315374	1911452	100.000

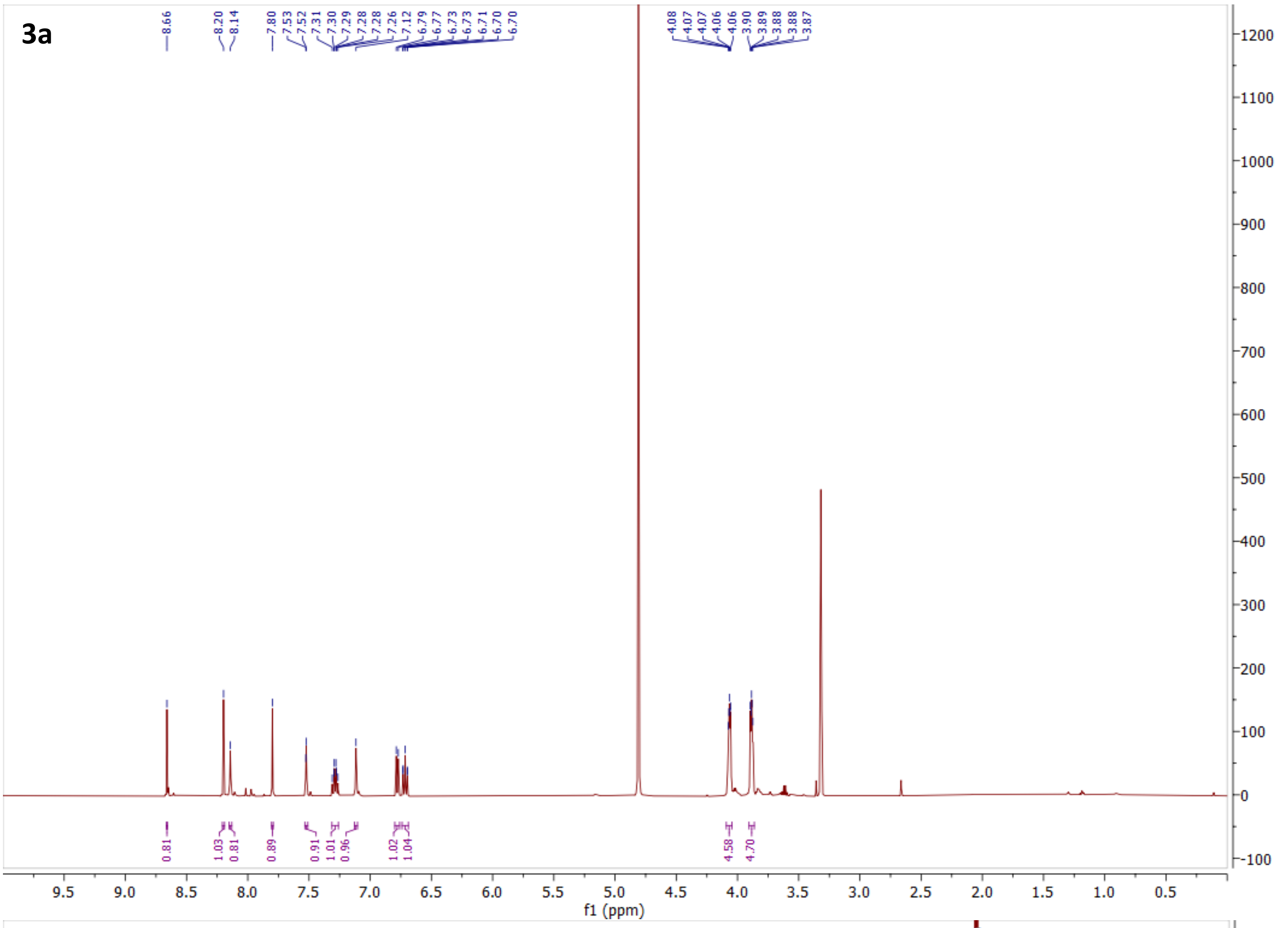
3a
f1 (ppm)

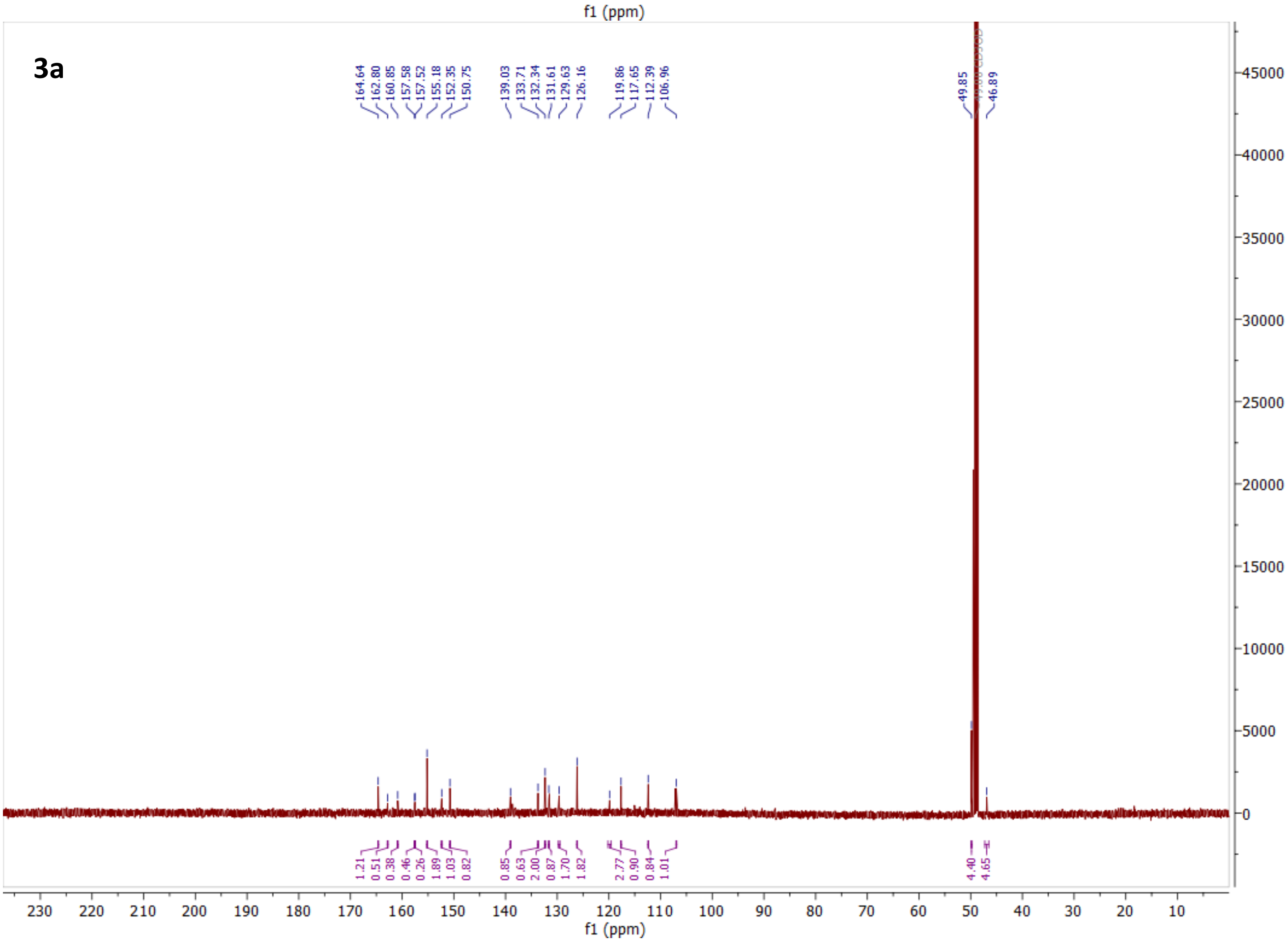
3a
f1 (ppm)

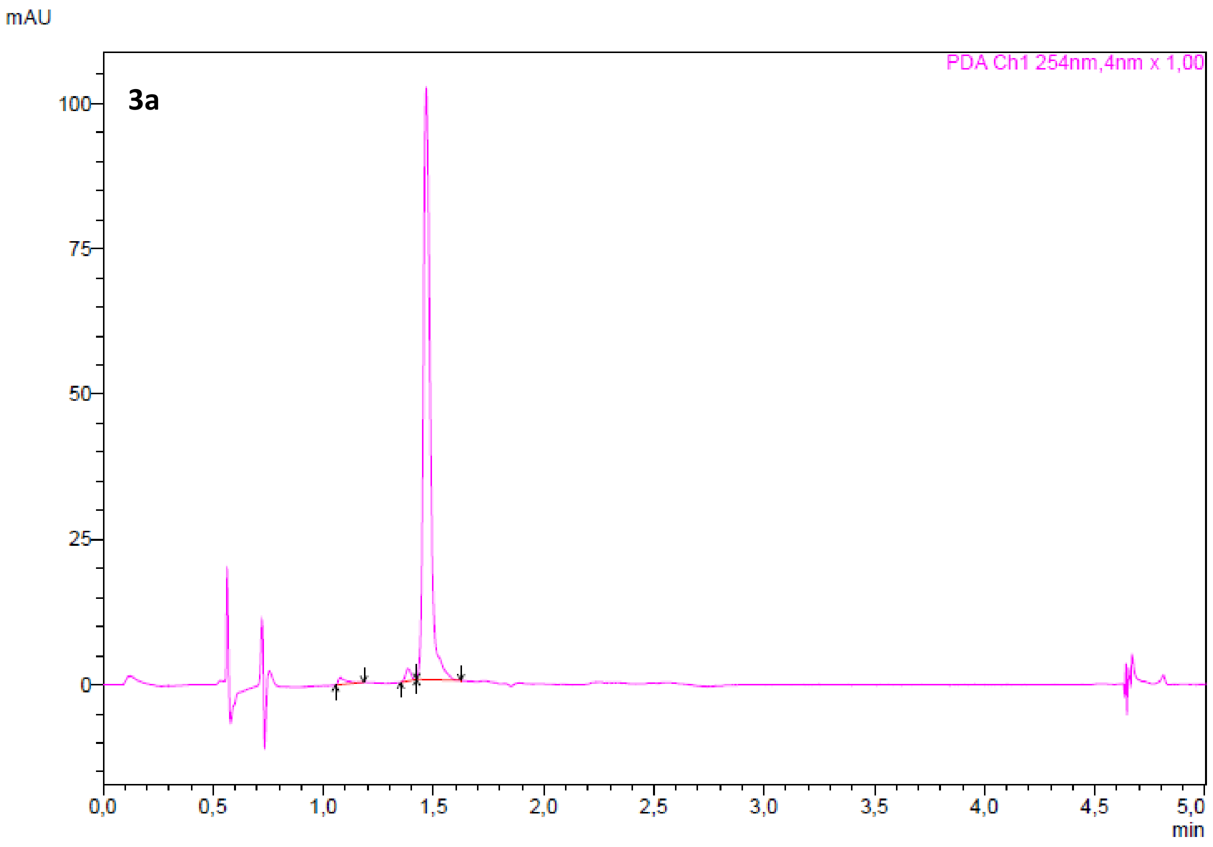

Peak#	Ret. Time	Height	Area	Area%
1	1.078	1213	3088	1.384
2	1.385	2015	3586	1.607
3	1.468	99853	216485	97.009
Total		103081	223159	100.000

3b

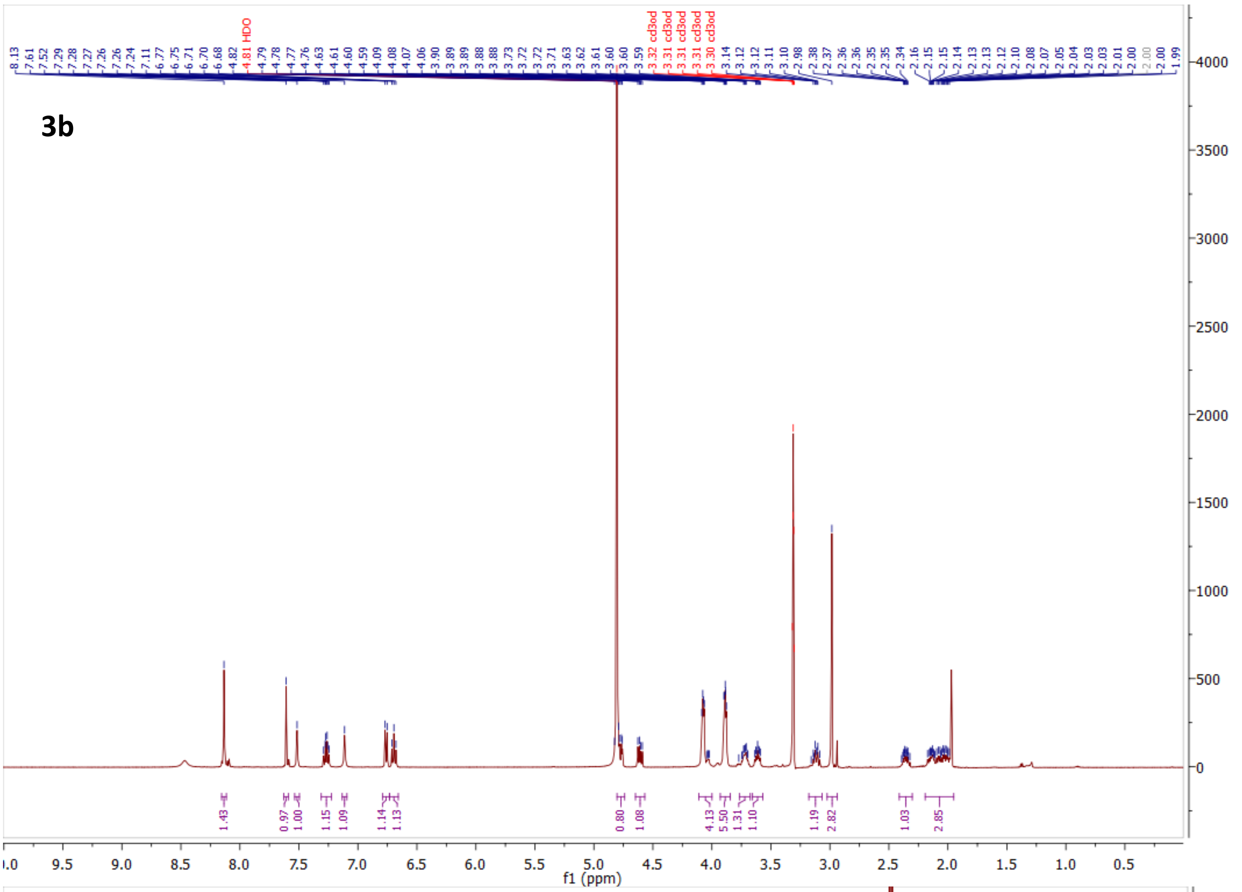

3b

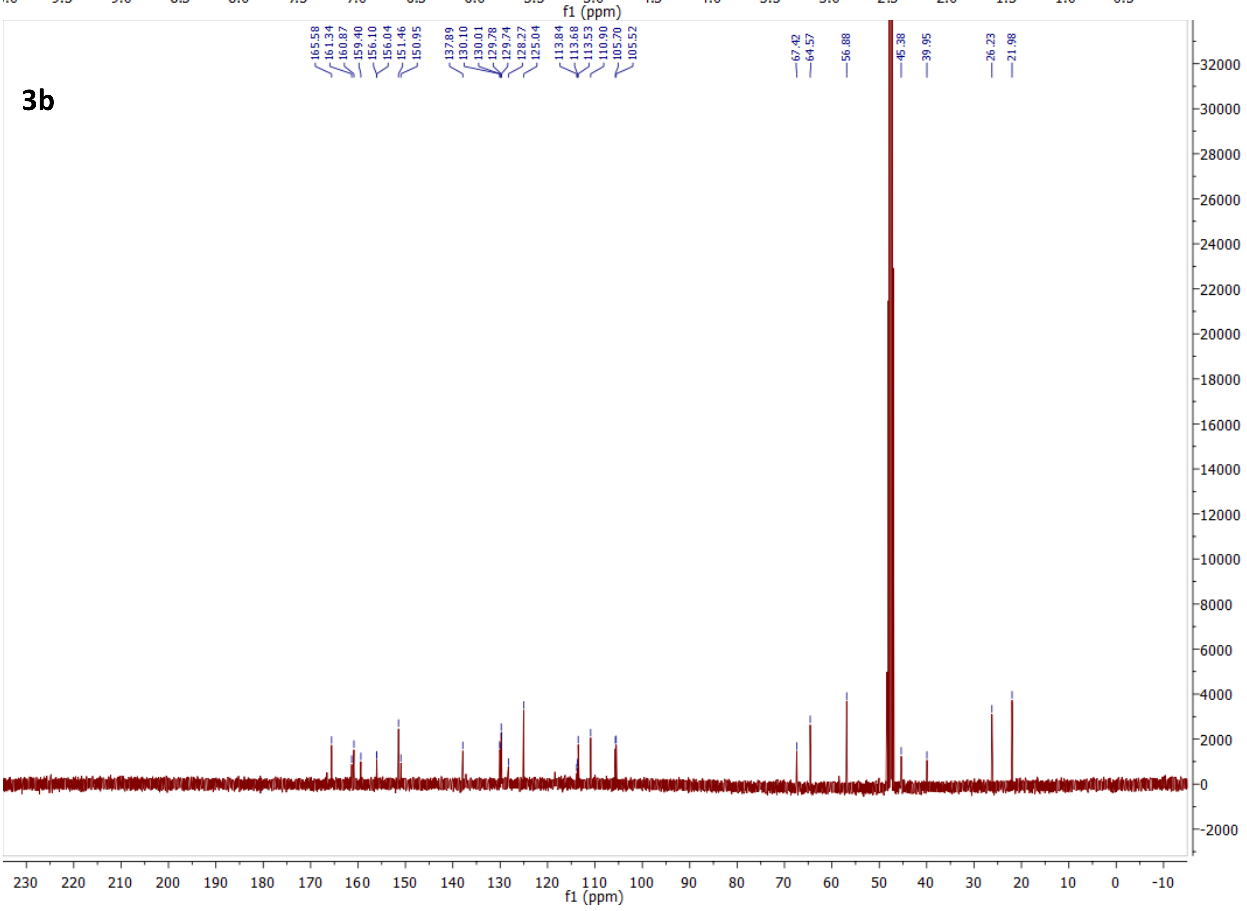

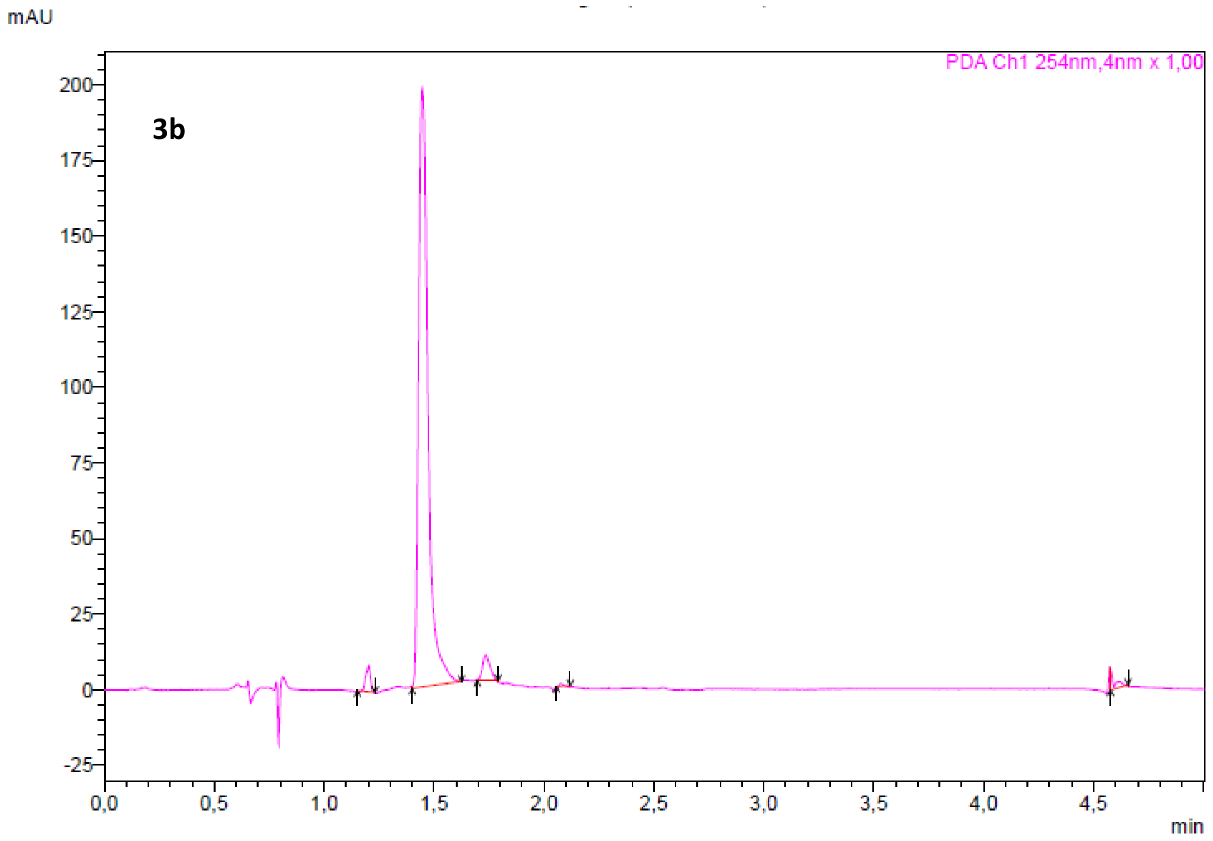

Peak#	Ret. Time	Height	Area	Area%
1	.1.202	7821	12343	1.970
2	1.736	195021	587612	93.800
3	2.077	8099	19512	3.115
4	4.577	806	51	0.008
5	1.448	6761	6932	1.107
Total		218508	626449	100.000

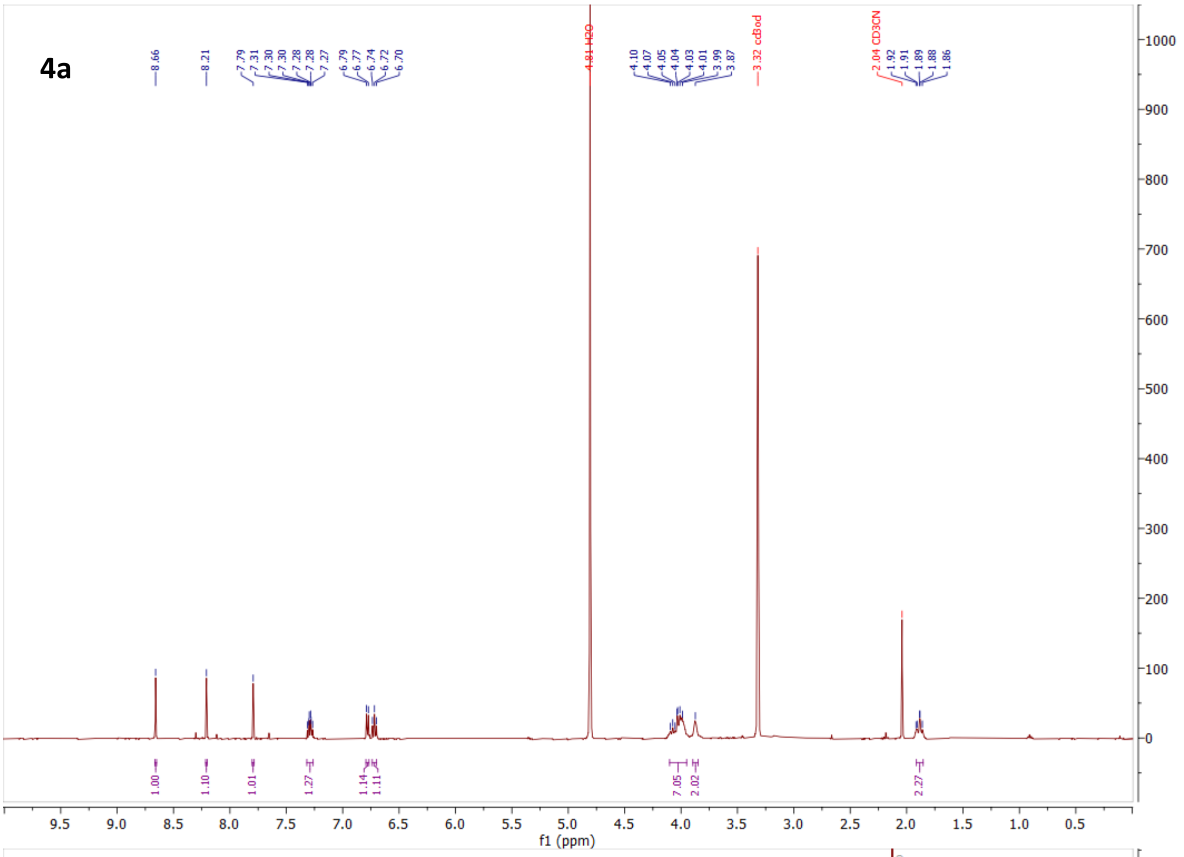
4a
f1 (ppm)

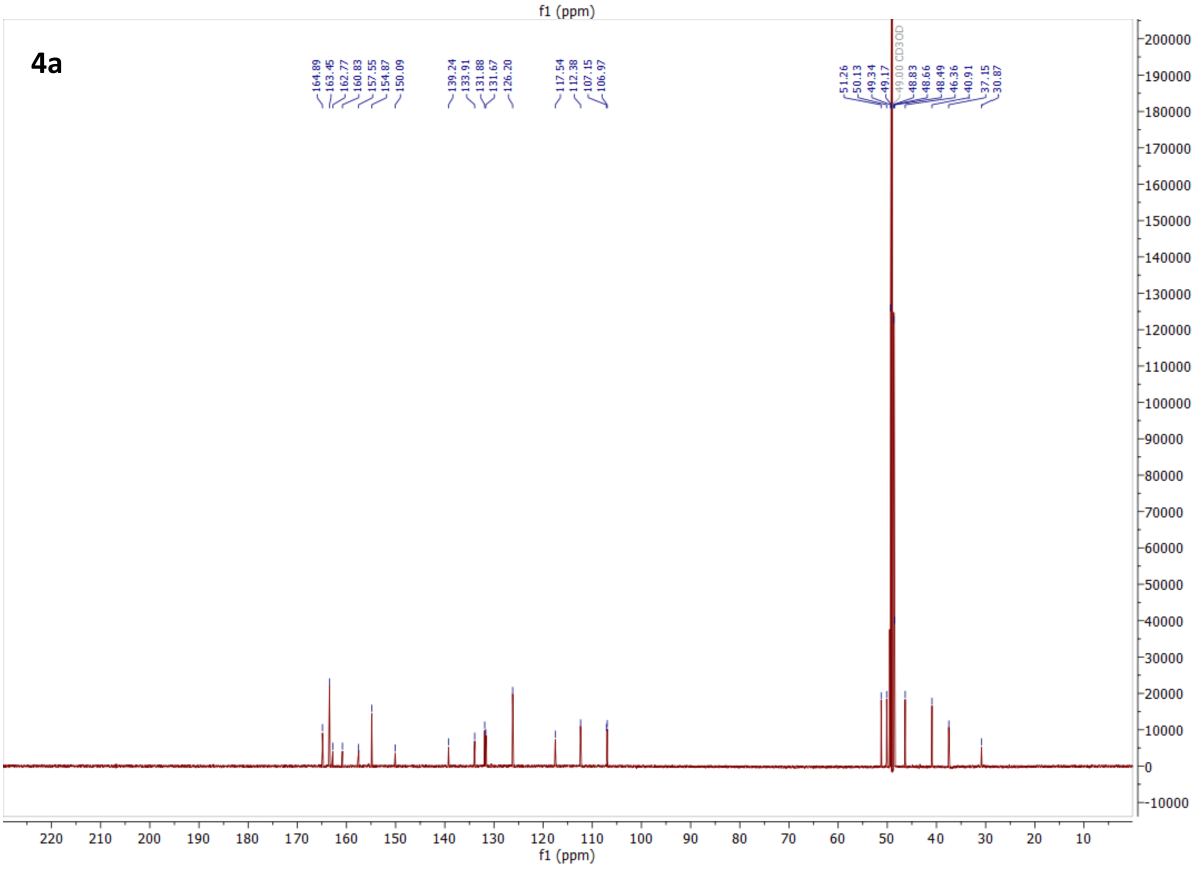
4a
f1 (ppm)

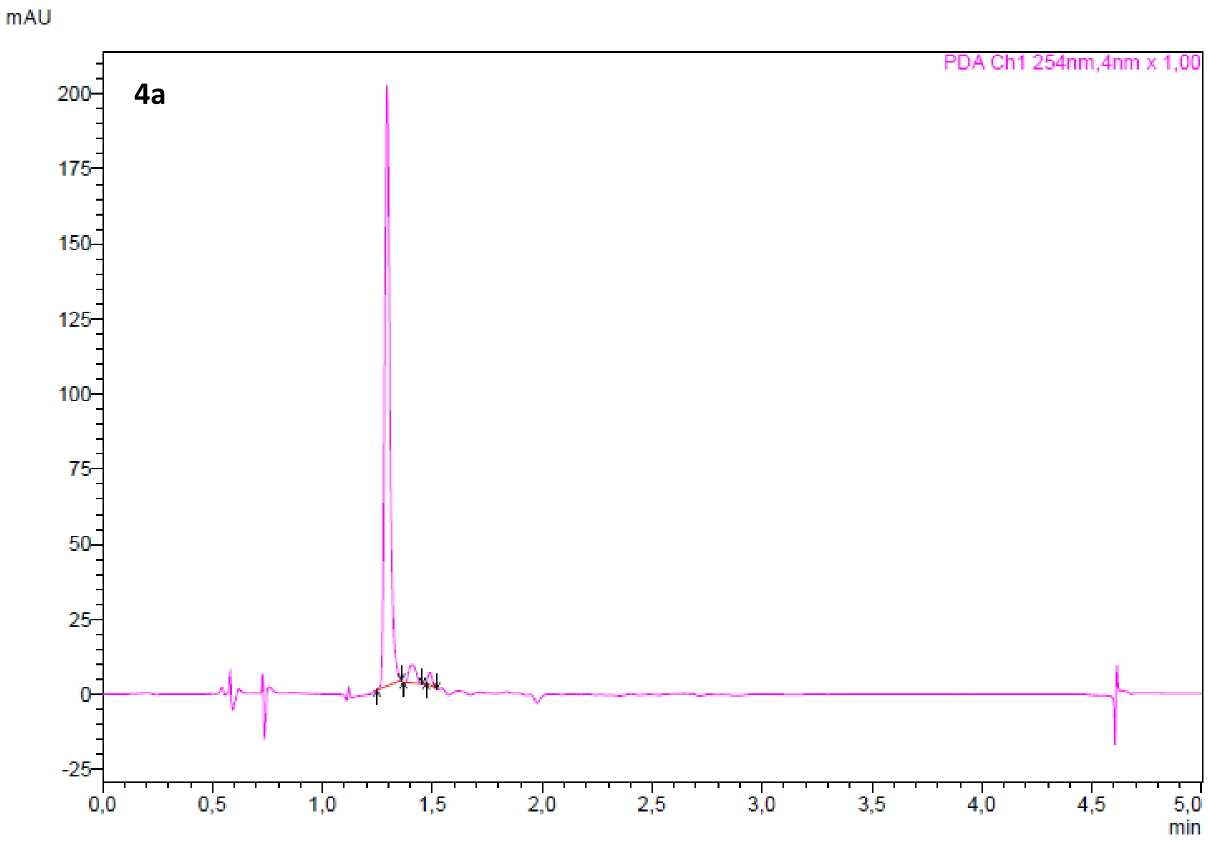

Peak#	Ret. Time	Height	Area	Area%
1	1.249	193396	329599	94.609
2	1.408	5834	13231	3.789
3	1.489	3852	5551	1.593
Total		203082	348380	100.000

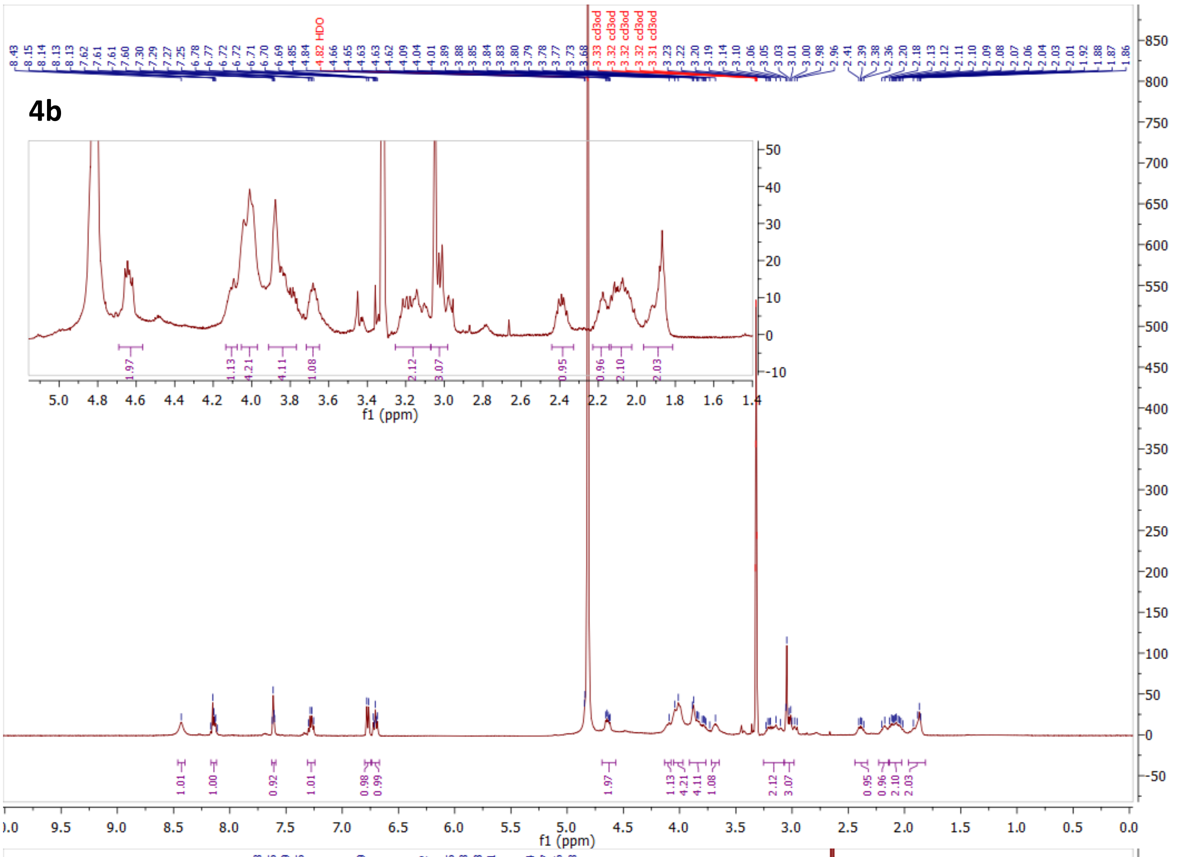
4b
f1 (ppm)

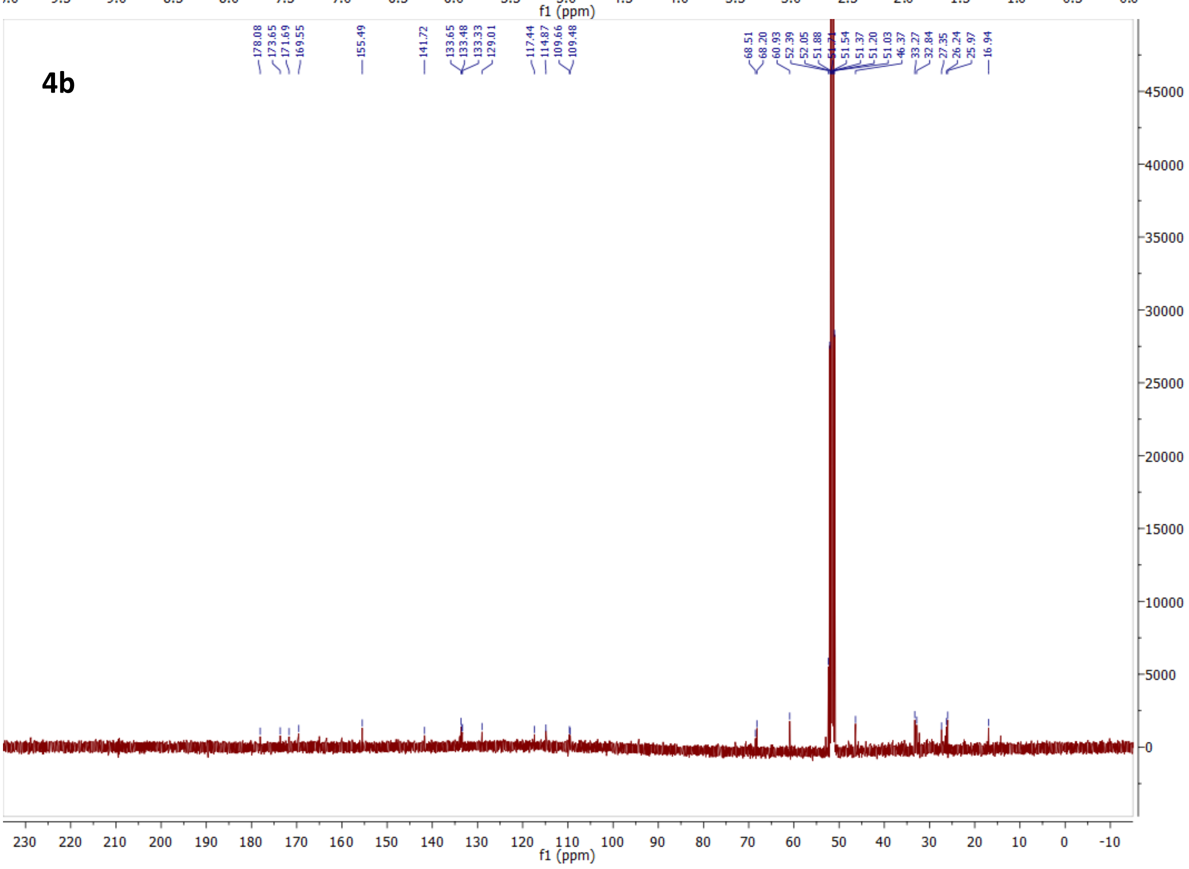
4b
f1 (ppm)

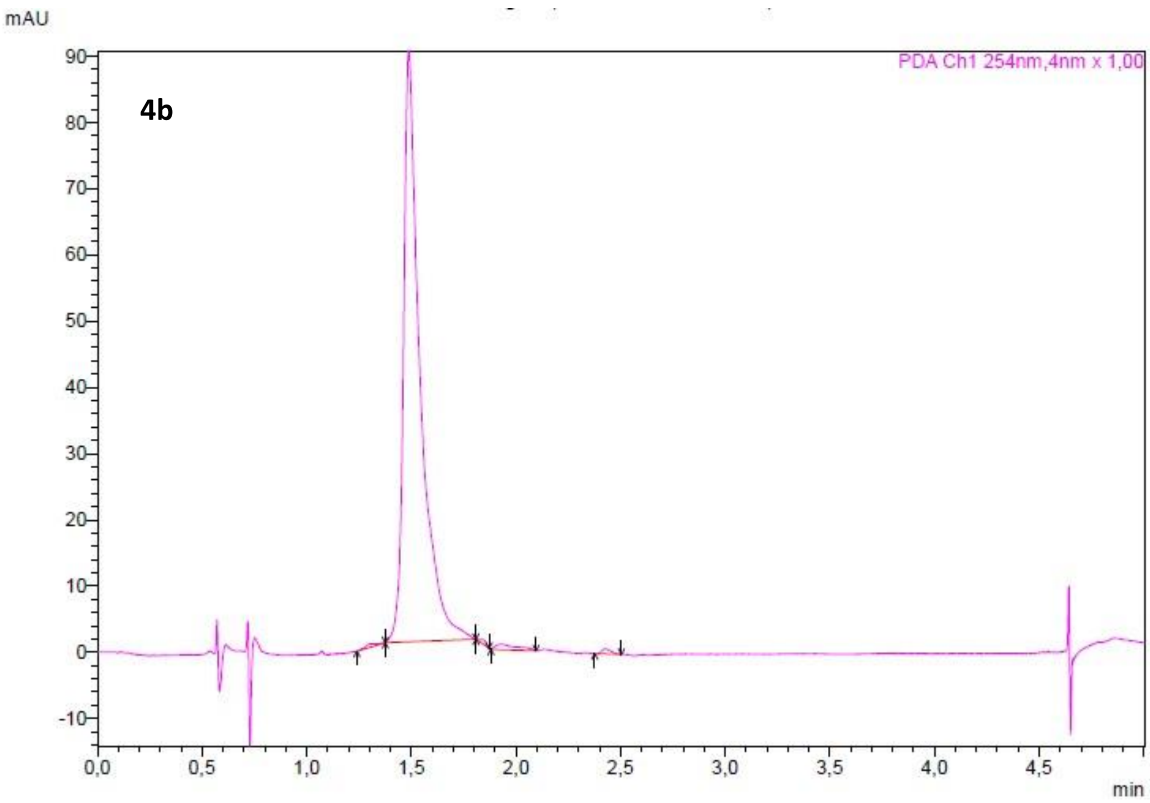

Peak#	Ret. Time	Height	Area	Area%
1	1.369	29	2056	0.406
2	1.488	87999	494185	97.715
3	1.817	132	1214	0.240
4	1.928	773	6063	1.199
5	2.426	745	2224	0.440
Total		89678	505742	100.000

LC-MS/MS peptide mapping data

Table S1 LC-MS/MS peptide mapping data: identification of KRasG12C peptides bearing 12Cys modification by compounds **1a-5b**.

Compound	Peptide sequence	Expected Peptide Mass (Da)	Calculated Peptide Mass (Da)	Mass difference	RT (Min)	Detected m/z	Charge State	Mass Error (ppm)
**1a**	GHMTEYKLVVVGACG	1562.7584	1974.8687	412.1103	32.8	494.7254	4	0.6
**1b**	GHMTEYKLVVVGACGVGKSA	2005.0124	2530.2068	525.1944	33.0	507.0503	5	1.9
	GHMTEYKLVVVGACGVGKS	1933.9753	2459.1697	525.1944	33.1	615.8004	4	0.1
**2a**	GHMTEYKLVVVGACGVGKSA	2005.0124	2403.1072	398.0948	32.5	601.7855	4	1.3
	GHMTEYKLVVVGACGVGKS	1933.9753	2332.0701	398.0948	32.4	584.0255	4	0.1
**2b**	GHMTEYKLVVVGACGVGKSA	2005.0124	2516.1912	511.1788	32.3	504.2456	5	-1.1
**3a**	GHMTEYKLVVVGACGVGKSA	2005.0124	2389.0913	384.0789	34.4	598.2807	4	-0.1
	LVVVGACGVGK	1000.5739	1384.6528	384.0789	36.5	462.5591	3	0.4
**3b**	GHMTEYKLVVVGACGVGKSA	2005.0124	2502.1755	497.1631	33.9	501.4425	5	-1.2
**4a**	GHMTEYKLVVVGACGVGKSA	2005.0124	2432.1335	427.1211	27.3	487.4353	5	1.3
	LVVVGACGVGK	1000.5739	1427.6949	427.121	26.8	476.9061	3	-0.4
**4b**	GHMTEYKLVVVGACGVGKS	1933.9753	2474.1807	540.2054	27.4	495.8449	5	1.8

Protein NMR spectra

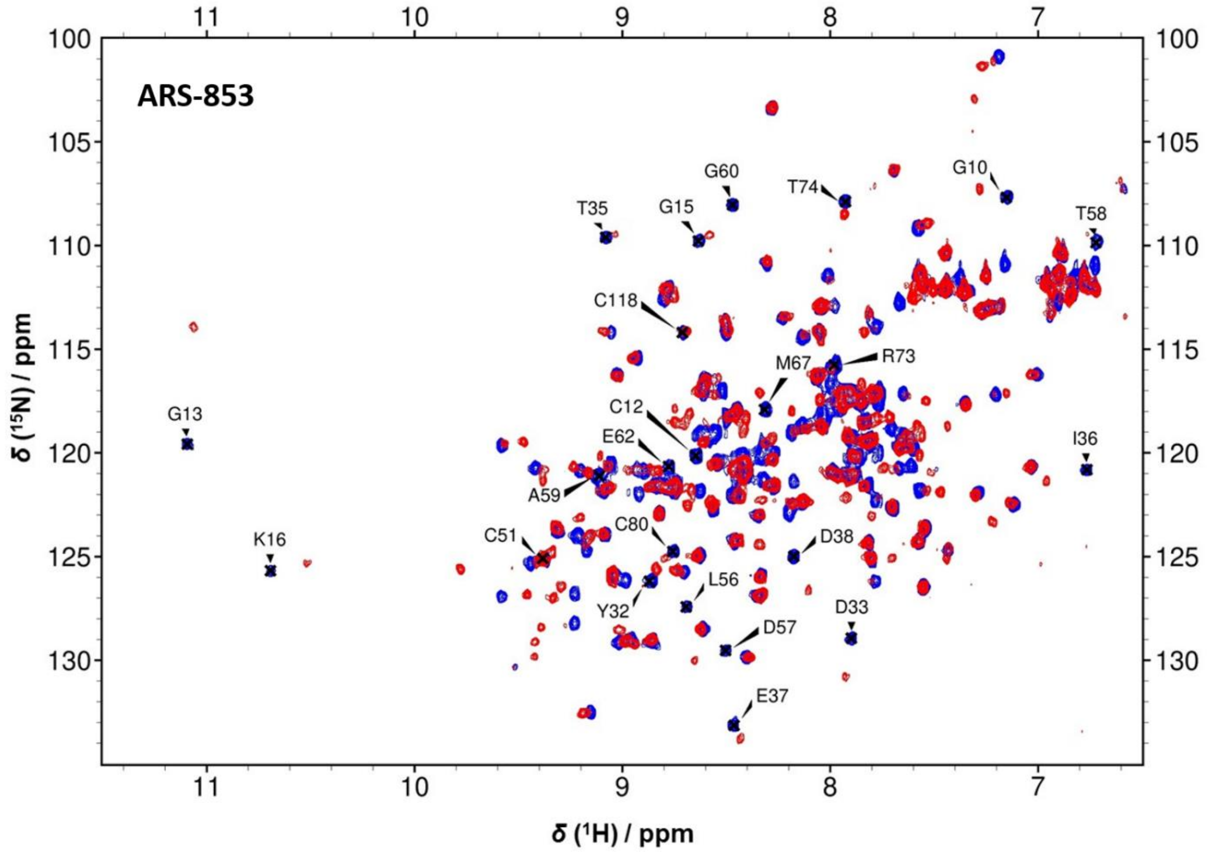

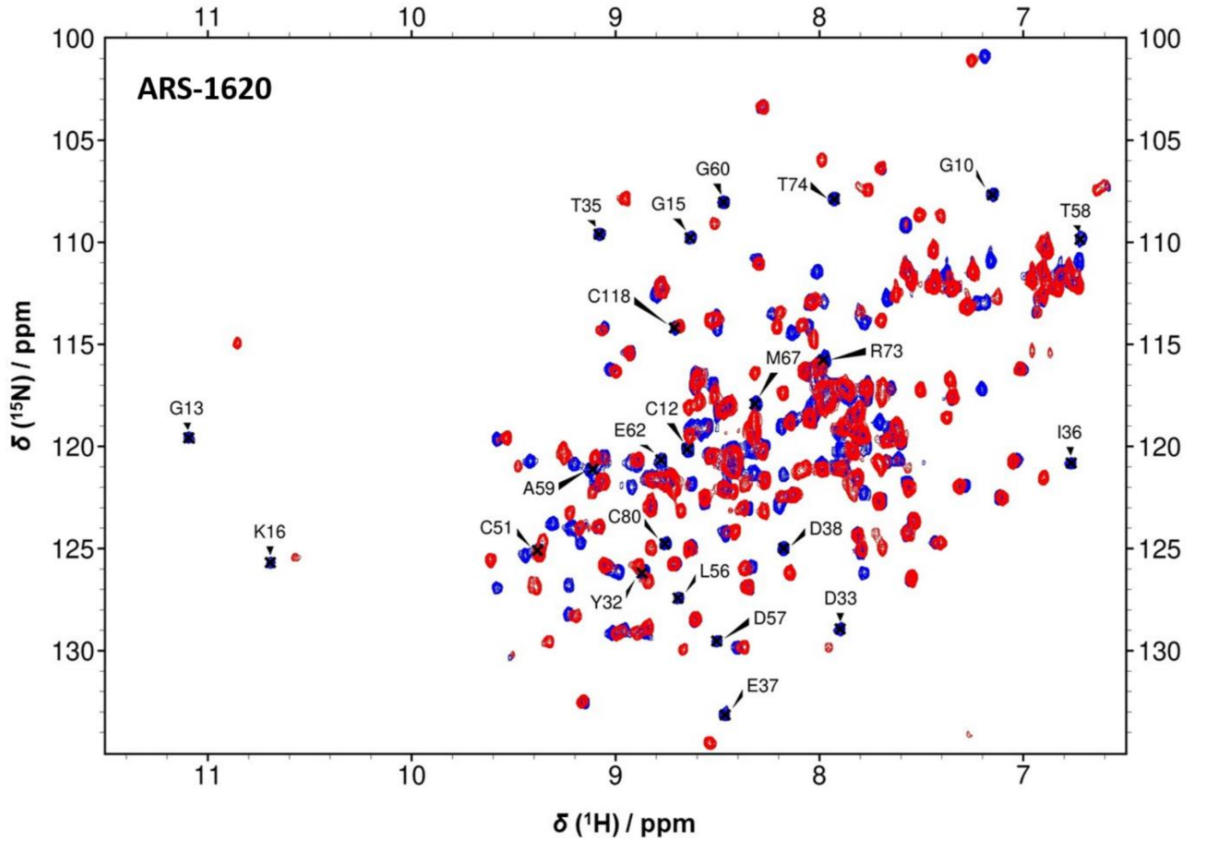

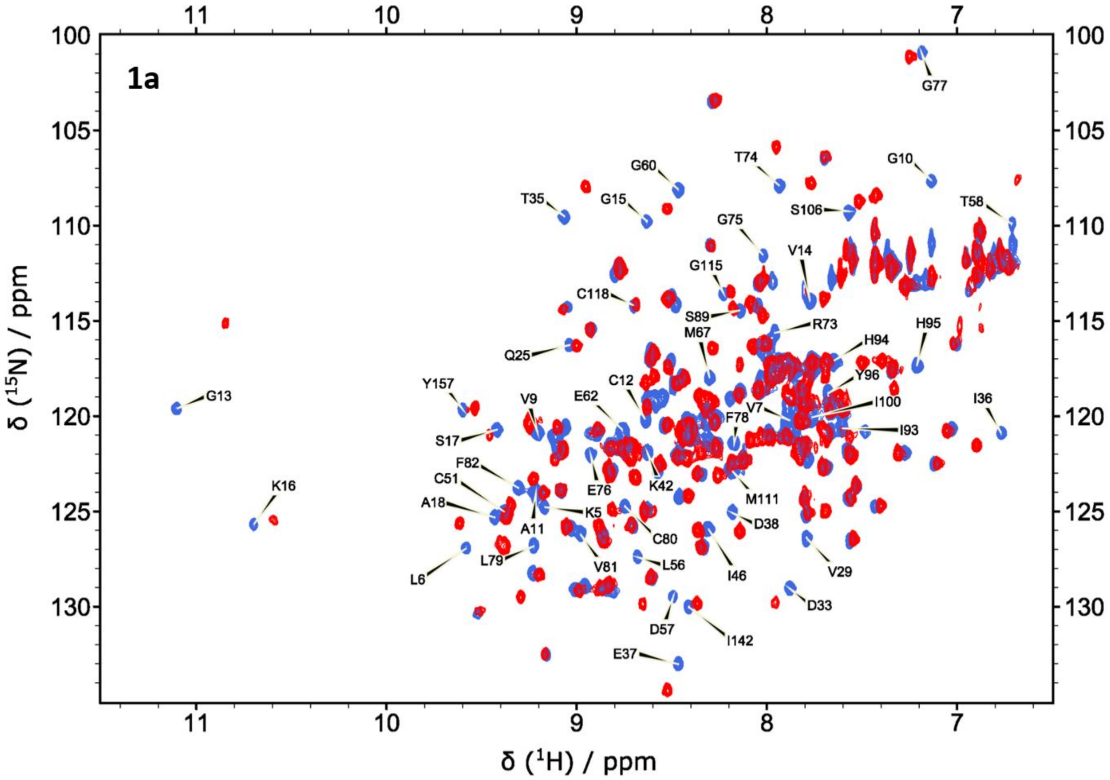
1a
δ (¹⁵N) / ppm
δ (¹H) / ppm
11
10
9
8
7
100
105
110
115
120
125
130
G77
T74
G10
G60
T35
G15
S106
T58
G75
V14
G115
C118
S89
M67
R73
H94
H95
Q25
Y96
C12
I36
Y157
E62
I100
G13
V9
F78
V7
I93
S17
F82
K42
K16
C51
E76
M111
A18
K5
D38
A11
C80
L79
V81
L56
I46
V29
L6
D33
D57
I142
E37
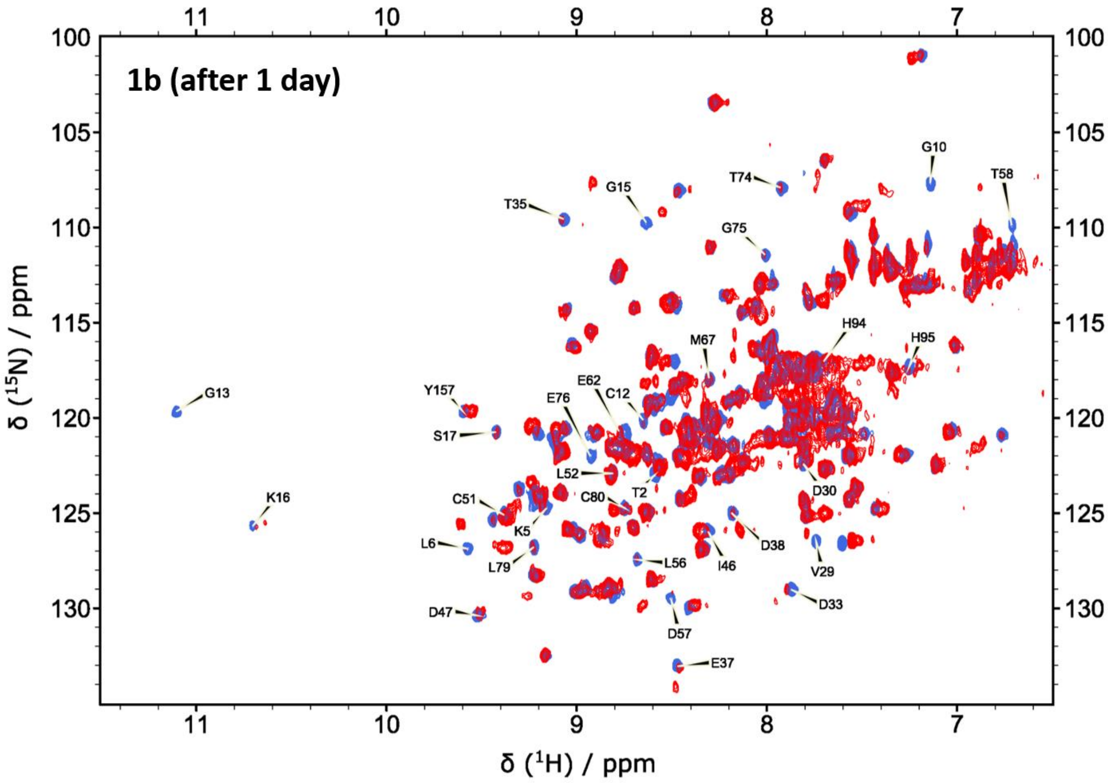
1b (after 1 day)
δ (¹⁵N) / ppm
δ (¹H) / ppm
11
10
9
8
7
100
105
110
115
120
125
130
G10
T74
T58
T35
G15
G75
H94
M67
H95
Y157
E62
G13
E76
C12
S17
L52
T2
D30
K16
C51
C80
K5
D38
L6
L79
L56
I46
V29
D47
D57
D33
E37

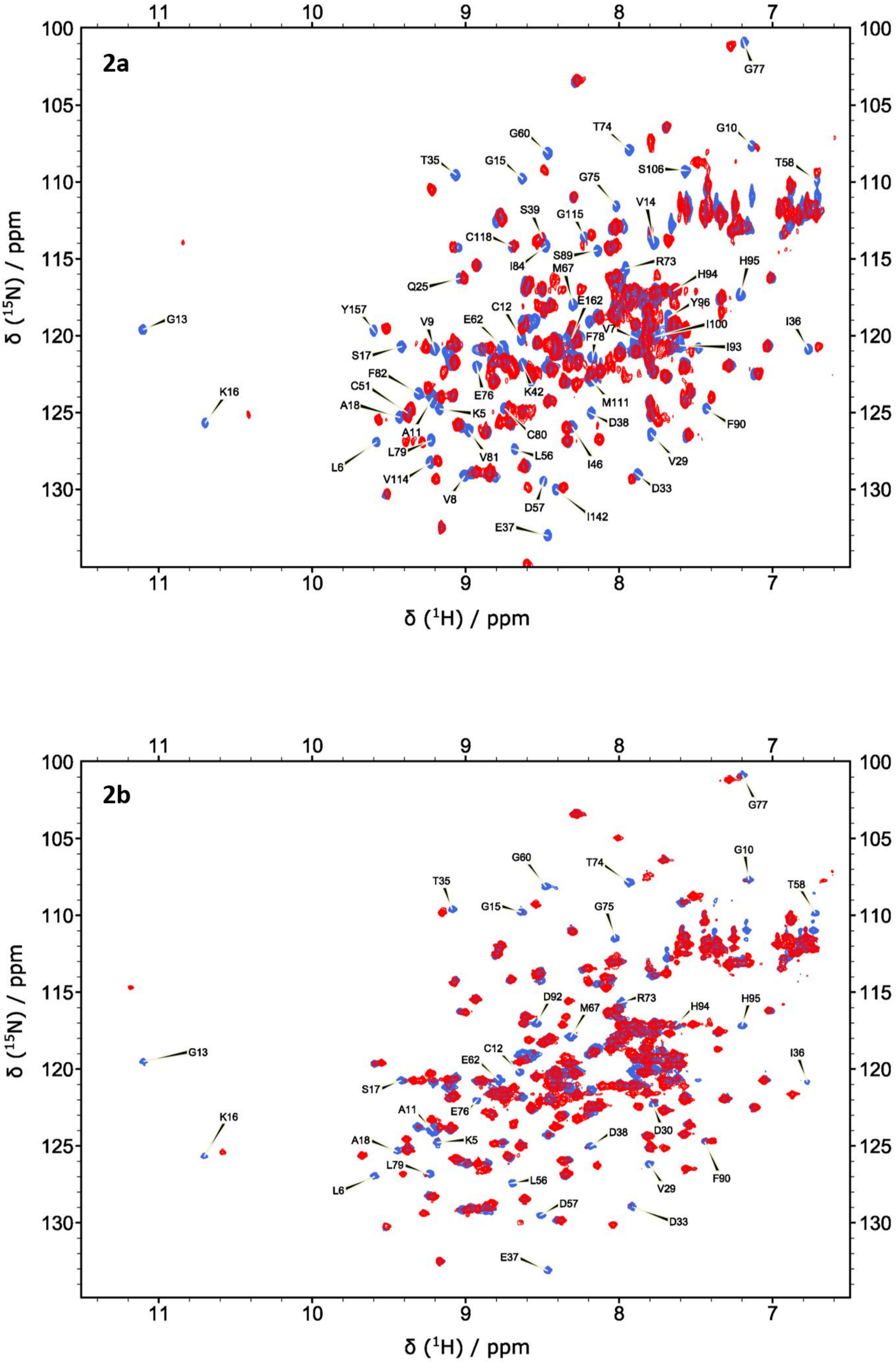
2a
G77
T74
G10
G60
T35
G15
S106
T58
G75
V14
S39
G115
C118
S89
I84
M67
R73
H94
H95
Q25
E162
Y96
C12
Y157
V9
E62
I100
I36
F78
V7
I93
S17
F82
K42
C51
E76
M111
A18
K5
D38
C80
F90
A11
L79
L56
V81
I46
V29
L6
V114
D33
V8
D57
I142
E37
G13
K16
δ (¹⁵N) / ppm
δ (¹H) / ppm
2b
G77
G60
T74
G10
T35
T58
G15
G75
D92
R73
H95
M67
H94
C12
I36
E62
S17
E76
A11
D30
D38
K5
A18
F90
L79
L56
V29
L6
D57
D33
E37
G13
K16
δ (¹⁵N) / ppm
δ (¹H) / ppm

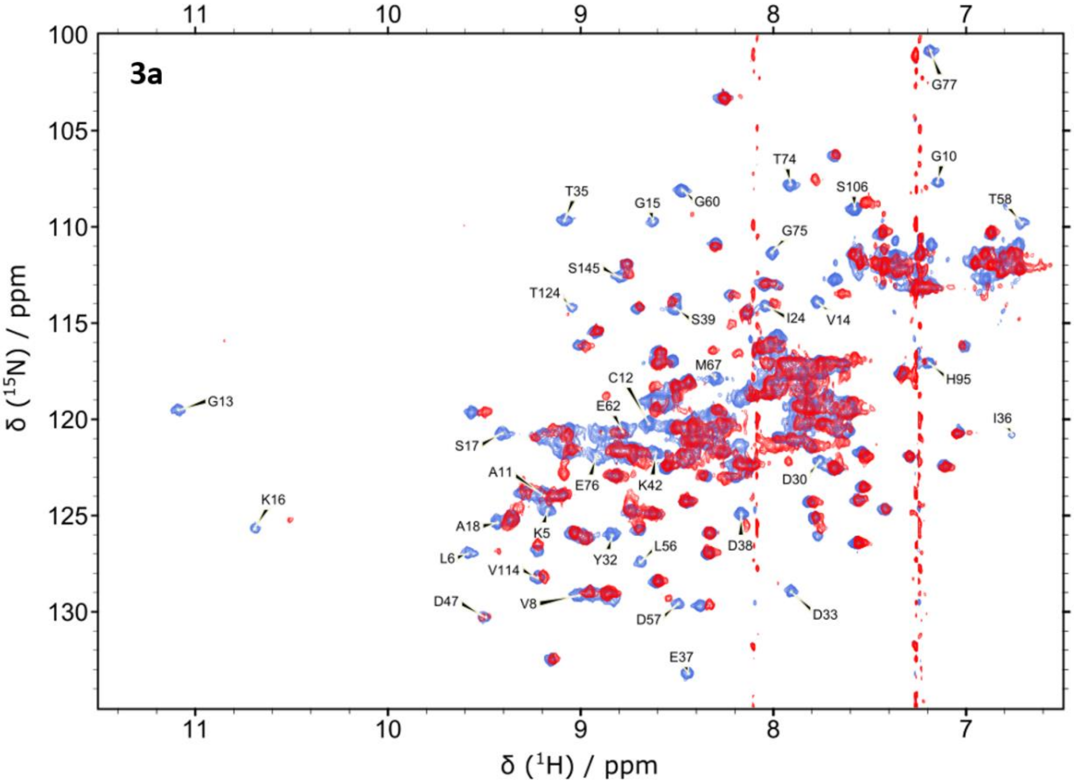
3a
δ (¹⁵N) / ppm
δ (¹H) / ppm
G77
T74
G10
S106
T35
G15
G60
T58
G75
S145
T124
S39
I24
V14
M67
H95
C12
G13
E62
I36
S17
A11
E76
K42
D30
K16
A18
K5
Y32
L56
D38
L6
V114
D47
V8
D57
D33
E37

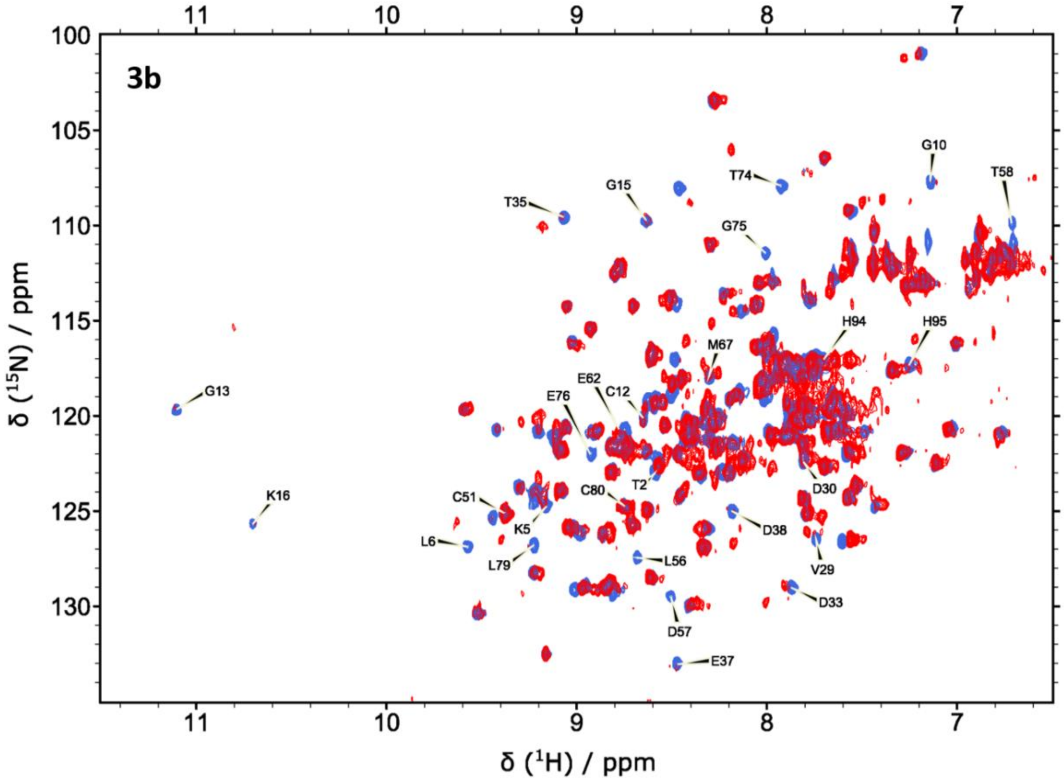
3b
δ (¹⁵N) / ppm
δ (¹H) / ppm
G10
T74
T58
T35
G15
G75
H94
H95
M67
E62
E76
C12
G13
T2
D30
C51
C80
K16
K5
D38
L6
L79
L56
V29
D33
D57
E37

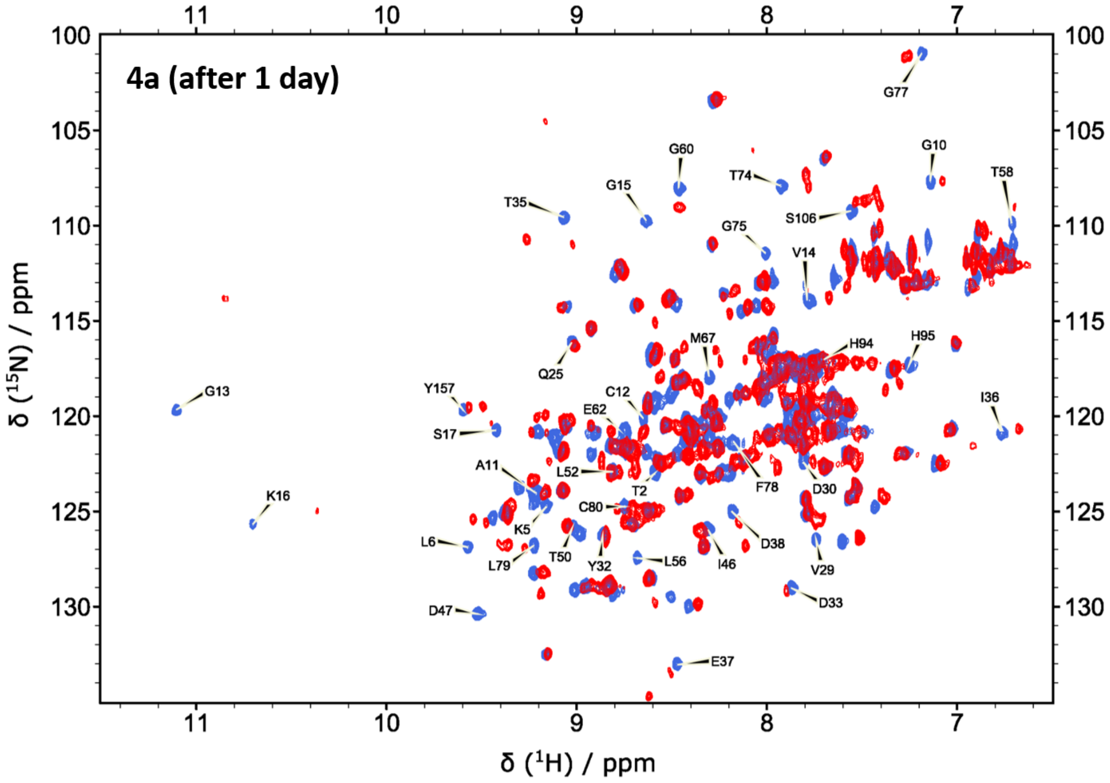
4a (after 1 day)
δ (¹⁵N) / ppm
δ (¹H) / ppm
G77
G60
G10
T74
T58
G15
T35
S106
G75
V14
M67
H94
H95
G13
Y157
C12
Q25
E62
I36
S17
A11
L52
T2
F78
D30
K16
C80
K5
L6
T50
D38
L79
Y32
L56
I46
V29
D33
D47
E37

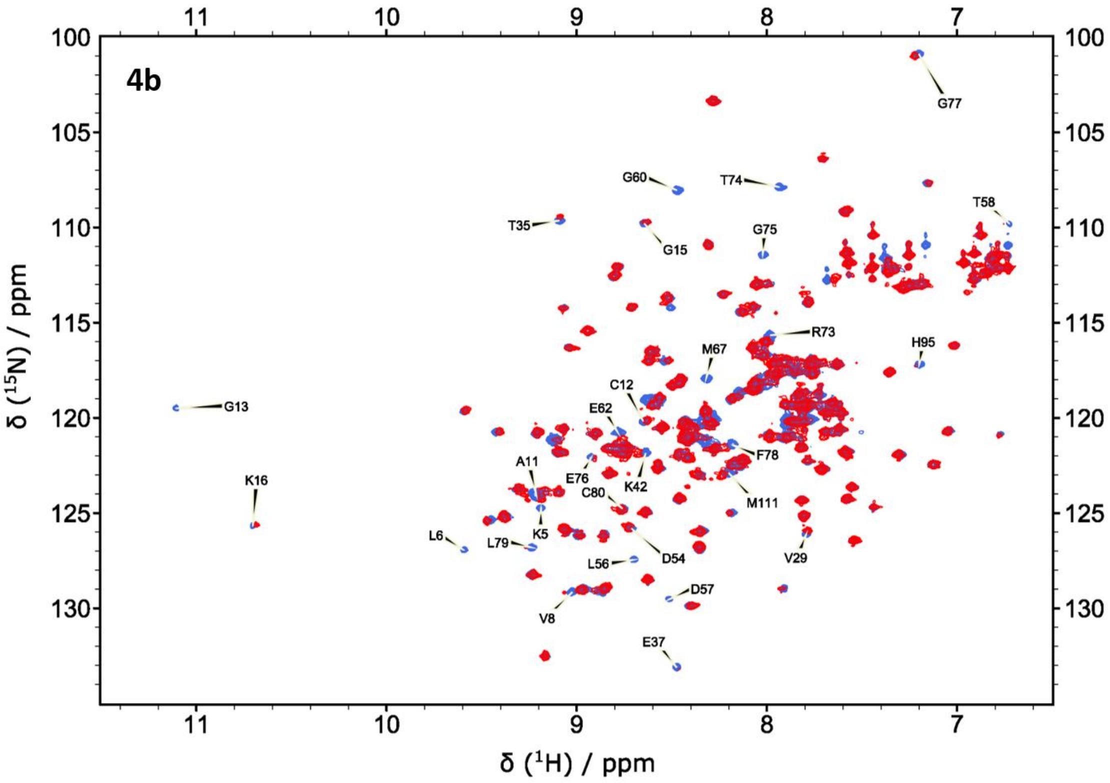
4b
δ (¹⁵N) / ppm
δ (¹H) / ppm
G77
G60
T74
T58
T35
G15
G75
R73
M67
H95
C12
G13
E62
F78
A11
E76
K42
C80
K16
M111
K5
L6
L79
D54
V29
L56
D57
V8
E37

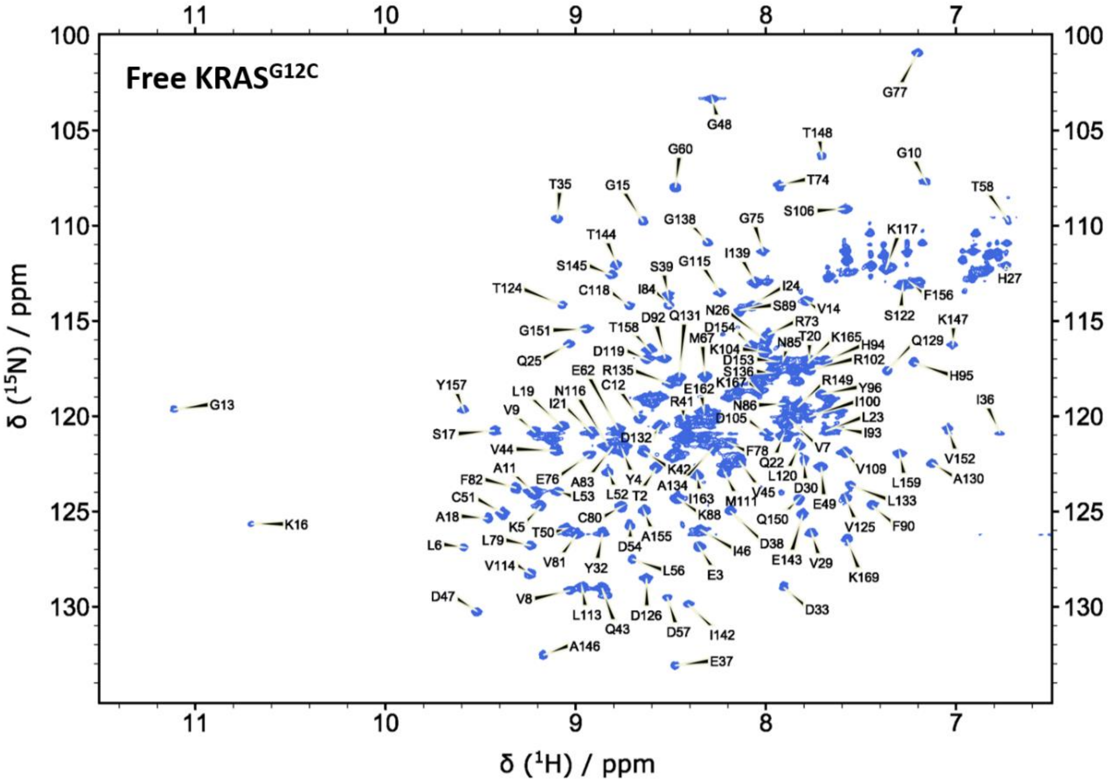

Figure S1 Overlaid ^{1}H, ^{15}N-SOFAST-HMQC (which is a fast version of HSQC) NMR spectra of KRAS^{G12C} interacting with the reference molecules (ARS-853 and ARS-1620), molecules **1-4a,b** are shown. The free KRAS^{G12C} protein spectra are colored blue, while the ligand-bound spectra are red. Residues showing the most significant chemical shift perturbation are shown.

kinact and KI determination

a)

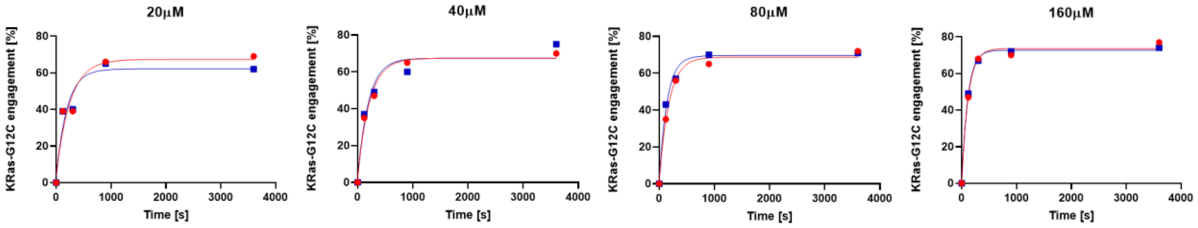

b)

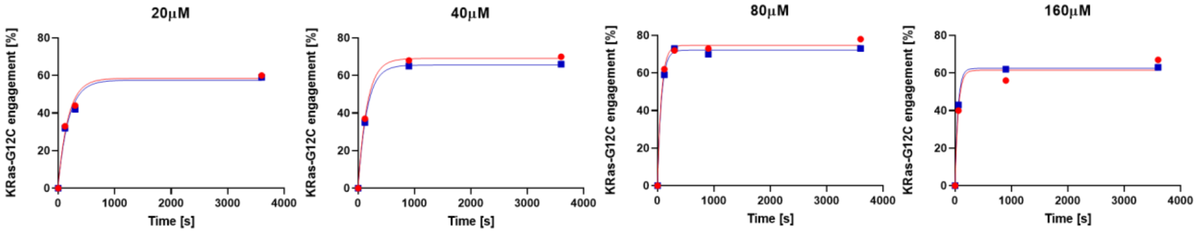

c)

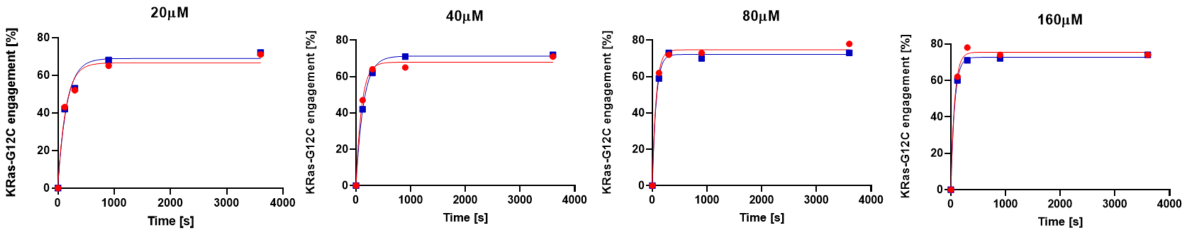

d)

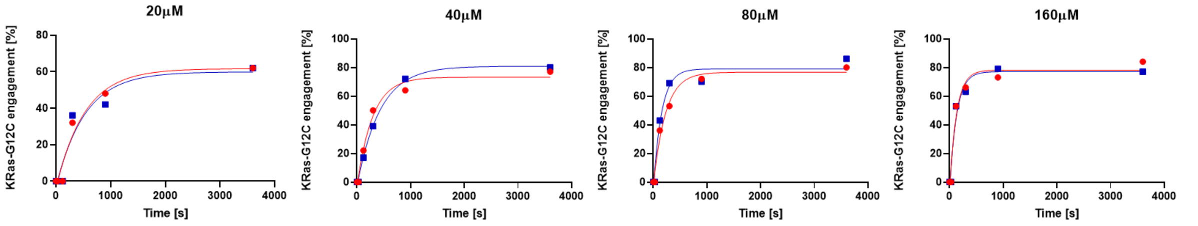

e)

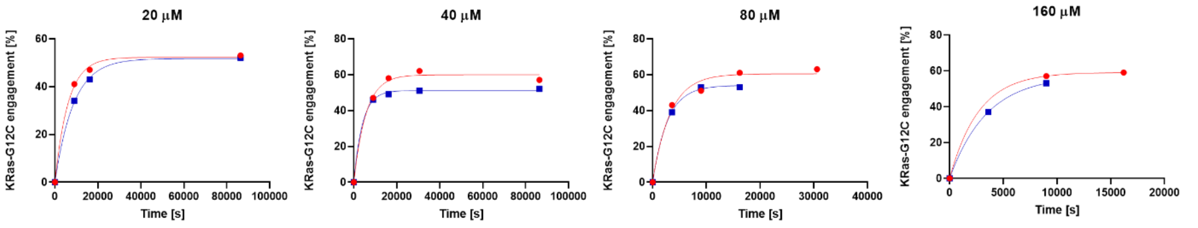

f)

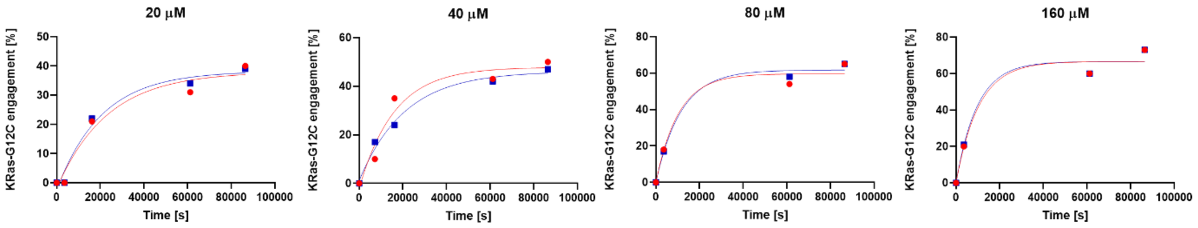

g)

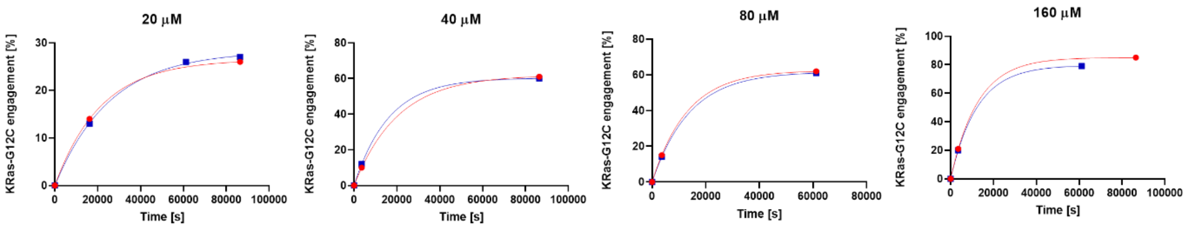

h)

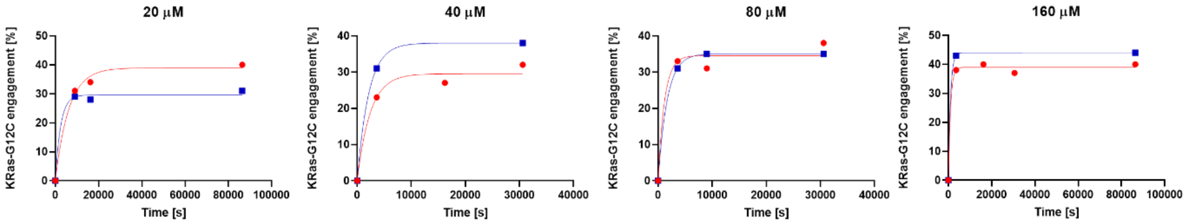

Figure S2 Time courses of KRAS^{G12C} covalent engagement. Data are shown for the duplicated experiments for each compound as follows: a) 1a; b) 1b; c) 2a; d) 2b; e) 3a; f) 3b; g) 4a and h) 4b. For each compound, the percent of covalently labeled KRAS^{G12C} was assessed by MS and exponential one-phase decoy regression was used (with fixed zero percent labelling at time zero) to calculate the k_{obs} values shown in **Table S2** and shown in **Figure S3**. For the experiments the KRAS^{G12C} concentration was 5 μM and the inhibitor concentration was varied from 20 to 160 μM, as shown.

Table S2 Calculated k_{obs} values for the 1a-4b compounds according to the covalent engagement of KRAS^{G12C} at different measured concentrations of the covalent probes (from 20 μM to 160 μM). Data are shown as results of duplicated experiments.

	20 μM	*40 μM*	*80 μM*	*160 μM*
**1a**	0.0456±0.0008 s^{-1}	0.0490±0.0003 s^{-1}	0.0640±0.0009 s^{-1}	0.0883±0.0005 s^{-1}
**1b**	0.0054±0.0005 s^{-1}	0.0069±0.0001 s^{-1}	0.0149±0.0019 s^{-1}	0.0185±0.0013 s^{-1}
**2a**	0.0067±0.0005 s^{-1}	0.0085±0.0018 s^{-1}	0.0144±0.0001 s^{-1}	0.0146±0.0001 s^{-1}
**2b**	0.0019±0.0001 s^{-1}	0.0029±0.0008 s^{-1}	0.0055±0.0015 s^{-1}	0.0077±0.0001 s^{-1}
**3a**	0.00014±0.00003 s^{-1}	0.00021±0.00005 s^{-1}	0.00033±0.00004 s^{-1}	0.00033±0.00006 s^{-1}
**3b**	0.00005±0.00001 s^{-1}	0.0006±0.00001 s^{-1}	0.00009±0.00001 s^{-1}	0.00010±0.00001 s^{-1}
**4a**	0.00004±0.00001 s^{-1}	0.0006±0.00001 s^{-1}	0.00007±0.00001 s^{-1}	0.00008±0.00001 s^{-1}
**4b**	0.00029±0.00018 s^{-1}	0.00044±0.00004 s^{-1}	0.00072±0.00018 s^{-1}	0.00104±0.00002 s^{-1}

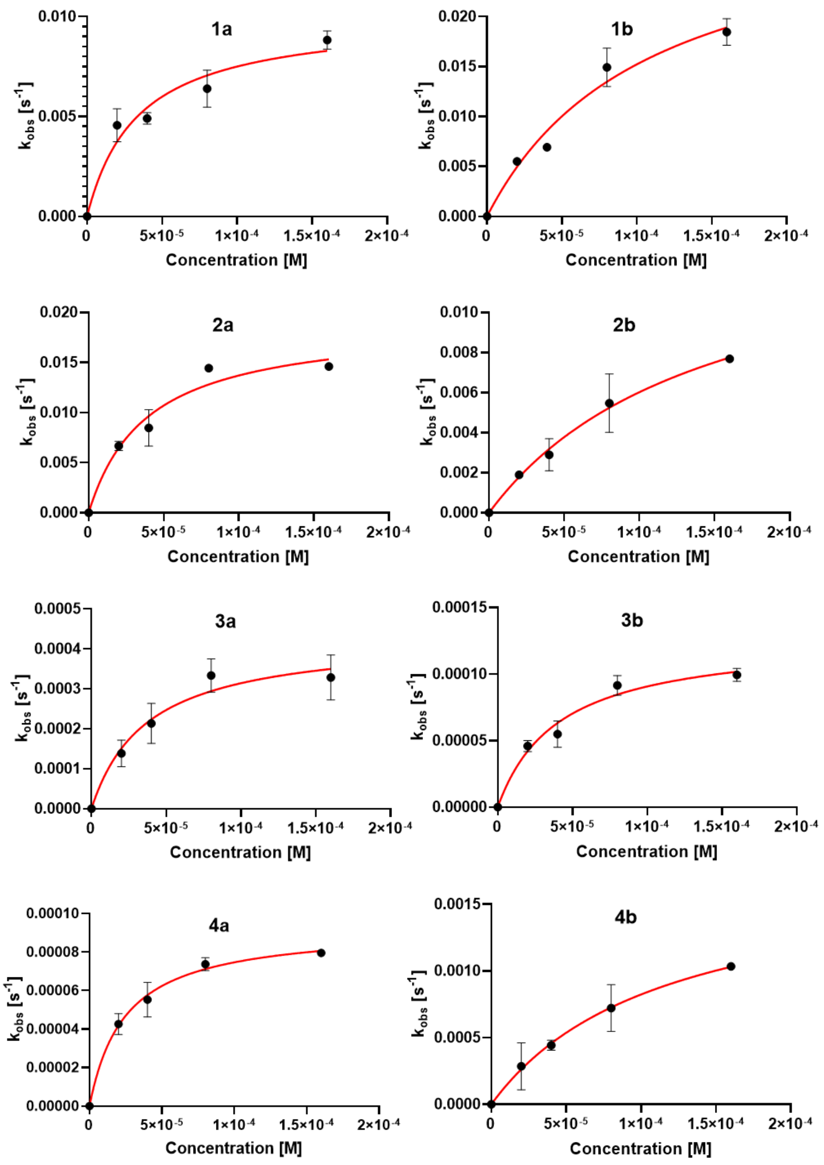

Figure S3 Determination of K_I and k_{inact} for the interaction of **1a**-**4b** covalent probes with $KRAS^{G12C}$. Calculated kobs values were plotted against the concentration of the probes and and K_I and k_{inact} were calculated directly from non-linear regression according to the k_{obs} – c function as follows: $k_{obs} = \frac{k_{inact} \cdot c}{K_I + c}$.

Computational data

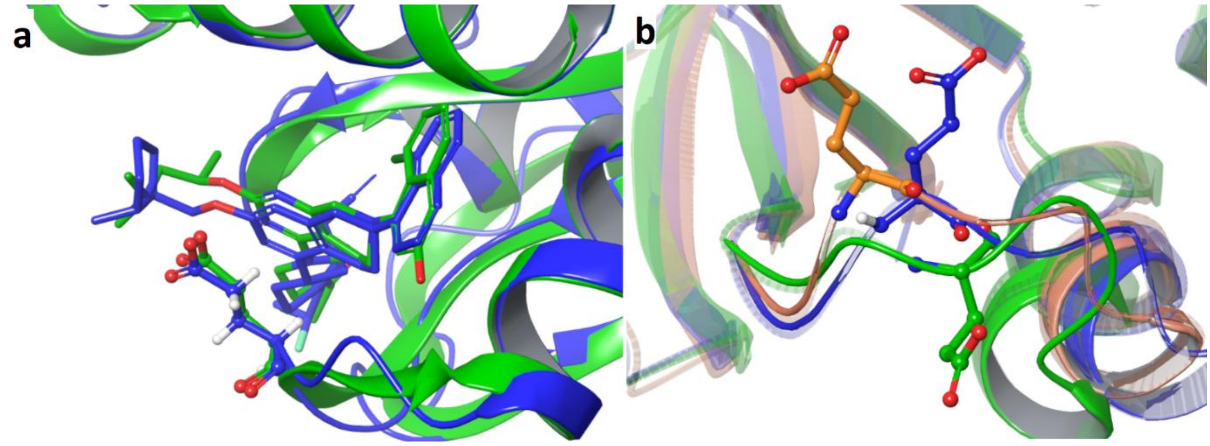

Figure S4 a) Superposition of KRASG12C complexes of ligands with basic side chains (PDB: 6UT0 blue and 6N2K green). Ligands are shown with stick and Glu62 with ball and stick representation. b) superposition of $KRAS^{G12C}$ complexes with varying loop conformations (PDB: 6UT0 blue, 6OIM green and 5V9U orange). Loops with sequences 59-70 are shown with bright colors. Glu62 are shown with ball and stick representation.

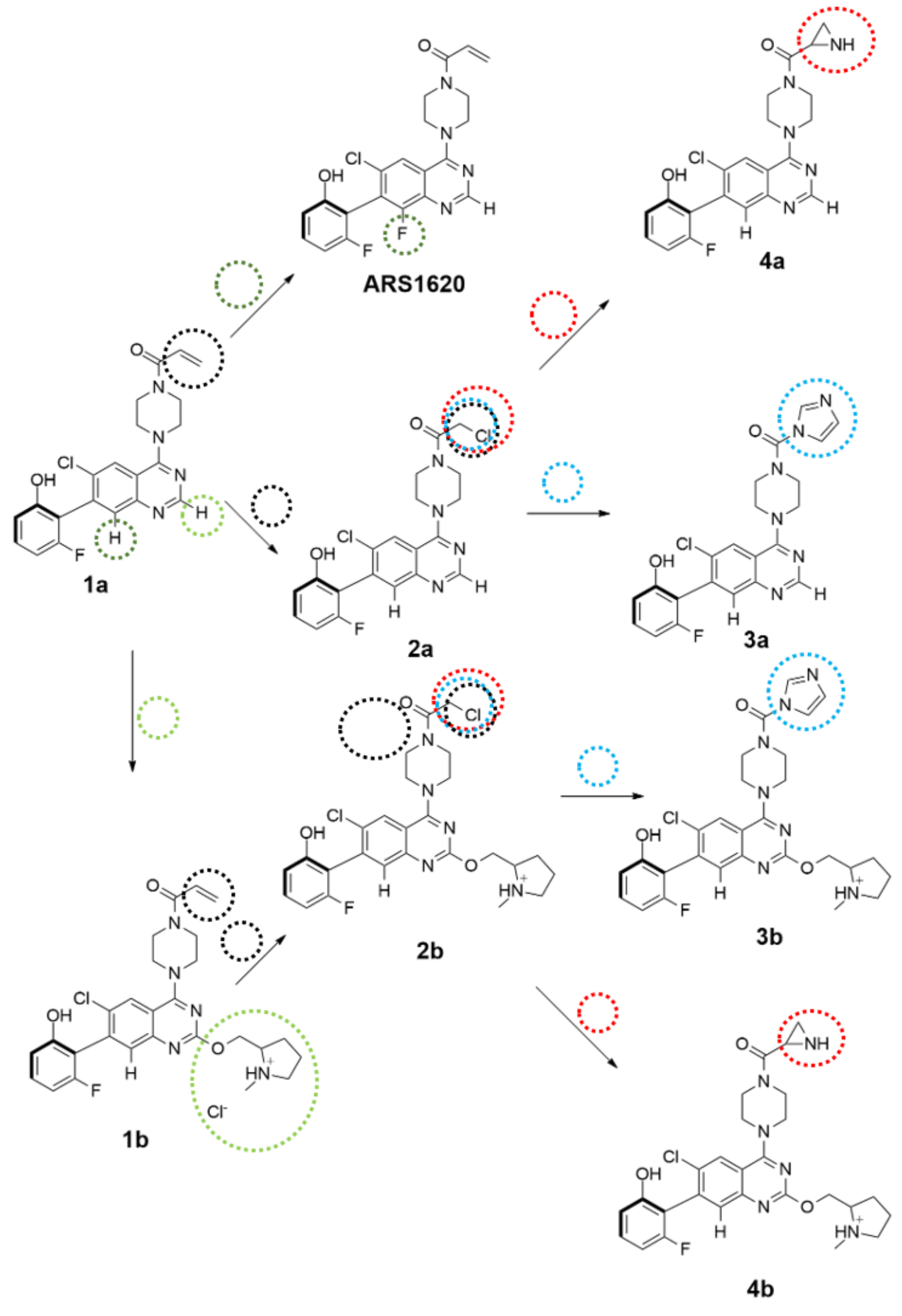

Figure S5 Transformation steps in the thermodynamic integration. The softcore atoms are shown with dashed lines with colours matching the corresponding steps.

Table S3 Calculated ΔΔG values for the parallel TI simulations along with the average values and the standard deviations of the transformation steps. The values in brackets correspond to the ΔG_{bind} values of the perturbed ligands (right side compounds in the first column) calculated by setting ΔG_{bind} = -6.1 kcal/mol for **1a**. All values are in kcal/mol.

**Transformation step**	**ΔΔG of run #1**	**ΔΔG of run #2**	**ΔΔG of run #3**	**AVG (ΔG_{bind})**	**STDev**
**1a → 1b**	-0.9	-1.7	-0.5	-1.0 (-7.1)	0.6
**1a → 2a**	-0.1	-0.2	-1.1	-0.5 (-6.6)	0.6
**1b → 2b**	-0.4	-0.5	-0.5	-0.5 (-7.6)	0.1
**2a → 3a**	0.6	2.0	2.3	1.6 (-5.0)	0.9
**2b → 3b**	3.2	2.5	1.6	2.4 (-5.1)	0.8
**2a → 4a**	0.7	1.0	1.2	1.0 (-5.6)	0.2
**2b → 4b**	0.4	0.3	0.6	0.4 (-7.1)	0.1
**1a → ARS1620**	-1.1	-1.0	-1.2	-1.1 (-7.2)	0.1

References

(1) Janes, M. R.; Zhang, J.; Li, L. S.; Hansen, R.; Peters, U.; Guo, X.; Chen, Y.; Babbar, A.; Firdaus, S. J.; Darjania, L.; Feng, J.; Chen, J. H.; Li, S.; Li, S.; Long, Y. O.; Thach, C.; Liu, Y.; Zarieh, A.; Ely, T.; Kucharski, J. M.; Kessler, L. v.; Wu, T.; Yu, K.; Wang, Y.; Yao, Y.; Deng, X.; Zarrinkar, P. P.; Brehmer, D.; Dhanak, D.; Lorenzi, M. v.; Hu-Lowe, D.; Patricelli, M. P.; Ren, P.; Liu, Y. Targeting KRAS Mutant Cancers with a Covalent G12C-Specific Inhibitor. *Cell* **2018**, *172* (3), 578-589.e17. https://doi.org/10.1016/j.cell.2018.01.006.
